# Reproducible Research: Computational Design of Personalized Clinical Treatments for Walking Impairments Using the Neuromusculoskeletal Modeling Pipeline

**DOI:** 10.64898/2026.03.02.709099

**Published:** 2026-03-04

**Authors:** Robert M. Salati, Geng Li, Spencer T. Williams, Benjamin J. Fregly

**Affiliations:** 1 Rice Computational Neuromechanics Lab, Department of Mechanical Engineering, Rice University, Houston, TX, United States

**Keywords:** Neuromusculoskeletal modeling, Musculoskeletal modeling, Predictive simulation, EMG-Driven Modeling, Computational Treatment Design, Muscle synergies

## Abstract

**Background:**

Personalized computational neuromusculoskeletal models have great potential for optimizing the design of clinical treatments for movement impairments. While many software tools address specific parts of the model personalization and treatment optimization processes, they typically require significant programming experience to use and do not cover the full breadth of these two processes. Furthermore, published neuromusculoskeletal modeling studies typically do not provide all of the minute methodological details needed for others to reproduce the work. Consequently, researchers seeking to develop skills in the model personalization and treatment optimization processes face a steep learning curve due to the lack of detailed training materials that demonstrate both processes for real-life clinical problems using real-life subject movement data.

**Methods:**

This article presents detailed training tutorials for the model personalization and treatment optimization processes using two real-life clinical problems and the Neuromusculoskeletal Modeling (NMSM) Pipeline. The first clinical problem involves the design of personalized gait modifications and high tibial osteotomy surgery for an individual with bilateral medial knee osteoarthritis, where the goal is to reduce the peak adduction moment in both knees to a specified target level. The second clinical problem involves the design of a synergy-based functional electrical stimulation prescription for an individual post-stroke with impaired walking function, where the goal is to equalize the propulsive and braking impulses between the two legs. Both tutorials were evaluated as course projects given to novice users in a combined undergraduate/graduate mechanical engineering course.

**Results:**

Both tutorials produced personalized neuromusculoskeletal models and associated dynamically consistent tracking optimizations that closely reproduced subject-specific experimental joint angles, joint moments, ground reaction forces and moments, and (if applicable) muscle activations measured during walking. Subsequent design optimizations predicted personalized treatments that achieved target values of peak knee adduction moments or propulsive and braking impulses.

**Conclusions:**

The detailed step-by-step tutorials presented with this article are the first to walk users step-by-step through the entire process of creating personalized neuromusculoskeletal models and then using them to design personalized treatments for clinical problems. These tutorials can be used to introduce new users to the NMSM Pipeline and as projects in neuromusculoskeletal modeling courses.

## Introduction

Neuromusculoskeletal (NMS) modeling has great potential for informing the clinical treatment design process. For NMS models to be used effectively to design treatments for movement impairments, they need to be personalized to each individual (i.e., a digital twin), where the aspects of an individual’s model that require personalization will depend on the clinical problem being addressed (1). For some clinical problems, personalized skeletal geometry developed from the individual’s imaging data may be necessary (2–11). Several software tools exist that can personalize skeletal geometry either with the use of imaging data (e.g., NMS Builder (12), The MAP Client (13–15), OpenSim Creator (16), Bone Deformation Tool (17), STAPLE (18), the work presented in Stansfield et al., 2025 (19)) and without the use of imaging data (e.g., The MAP Client (13), Torsion Tool (20)). For other clinical problems, personalized joint structure, muscle-tendon, neural control, and foot-ground contact model components calibrated using the individual’s movement, force, and electromyographic (EMG) data may be necessary (3,21–28). Several tools exist that can personalize some, but not all, of these model components (e.g., AddBiomechanics (29), CEINMS (30), CEINMS-RT (31), SimCP (32)). Consequently, creation of a digital twin possessing personalized properties for these model components is either not possible or is challenging due to the need to use a combination of tools.

Once individual-specific NMS models are available, they can be used to predict computationally how the individual will function following a planned clinical intervention (3,21,23,33). This computational process, termed “predictive simulation,” is itself challenging, historically requiring specific skills in computational simulation and numerical methods to perform. To date, most predictive simulations of gait using NMS models have been performed using in-house software (21,34–42), but such tools are difficult for other researchers to use for their own research purposes. Consequently, several research groups have recently developed software tools that make the predictive simulation process easier to learn, facilitating replication of research performed by others. OpenSim Moco (43–45) has made predictive simulation much more accessible within the OpenSim simulation environment, while other software tools facilitate generation of predictive simulations either partially or completely outside of the OpenSim environment (e.g., BioMAC-Sim-Toolbox (46), SCONE (47), OpenSim-Matlab Optimal Control (48), MyoSuite (49), GaitDynamics (50)). Each of these tools can be used to generate complex simulations that predict the functional outcome of a planned treatment, but none of them were designed to interface directly with model personalization software. For this reason, nearly all published predictive simulations of human movement have utilized scaled generic models and predict generic movements rather than utilizing personalized models that predict individual subject movements.

For model personalization and predictive simulation alike, training new researchers to use these valuable software tools poses a significant challenge. If a new researcher wants to generate predictive simulations of an individual’s post-treatment movement function using a personalized NMS model of the individual (henceforth called “treatment optimization”), they will need to learn a combination of tools and write code that interfaces the tools with each other. Most tools mentioned thus far have well-developed introductory tutorials that teach new users about basic and some advanced functionality (51–56). However, most of these tutorials are not “real world” but rather work through smaller problems designed to teach specific isolated functionality. The tutorials also never work through the entire clinical treatment design process starting with model personalization and ending with treatment optimization. Due to their reduced scope, tutorials typically fail to communicate the nuances of tool use, which are critical for determining if and how a particular tool could be used to address a particular research goal. Thus, to learn about how to apply these tools to real-life clinical problems, researchers need to study published research articles. Unfortunately, reproducing published research is often challenging in its own right, as most research articles report only final results with relevant data and source code. However, key implementation details are often missing that prevent other researchers from reproducing and expanding upon the work. This situation has resulted in a “reproducibility crisis” that significantly hinders progress of the field (57). If new researchers want to learn how to use existing software tools to create personalized predictive simulations that can be used for clinical treatment design, they must be resourceful, tenacious, and technically adept to assemble the necessary knowledge.

A major reason for this lack of “real-life” training materials for model personalization and treatment optimization is that existing software tools do not cover the entire process, so at best, tool developers can create tutorials that cover only isolated areas. To create real-life training materials that teach users the entire model personalization and treatment optimization process, we need a toolset that covers the full spectrum of these two processes. This problem has been addressed by the recently published Neuromusculoskeletal Modeling (NMSM) Pipeline (58). The NMSM Pipeline is a MATLAB-based open-source software package with integrated Model Personalization and Treatment Optimization toolsets. All tools within each toolset were designed to be used sequentially, with Model Personalization tools naturally interfacing with Treatment Optimization tools with minimal effort from the user. The inputs to the NMSM Pipeline are a scaled generic OpenSim (44,45) model with or without personalized skeletal geometry (as appropriate) and individual subject movement data. Users can then personalize relevant joint structure, muscle-tendon, neural control, and foot-ground contact (as appropriate) model components for an individual subject and then use the subject’s personalized model in the treatment optimization process. Users can perform the entire model personalization and treatment optimization process without writing a single line of code. Instead, they create all necessary tasks using Extensible Markup Language (XML) settings files, created either in an OpenSim graphical user interface or by manual editing of XML files, which significantly lowers the barrier to entry for these high-end NMS modeling and simulation capabilities. This low barrier to entry can significantly increase the number of potential users who can explore computational treatment design using personalized NMS models.

Although the NMSM Pipeline makes treatment design with predictive simulations easier than it was previously, the tools still require significant training to learn. Basic tutorials for the NMSM Pipeline have already been made available (59), but like other musculoskeletal modeling software packages, these tutorials do not capture the nuances and details that should be understood to use the tools effectively for addressing real-life clinical problems. There is still a need for detailed tutorials that work through the entire model personalization to treatment optimization process for real-life clinical problems. Because the NMSM Pipeline makes model personalization and treatment optimization accessible and connected by a single workflow, it is now possible to design such in-depth tutorials. These tutorials can focus not only on what users are doing and why they are doing it, but also on how the final results are obtained.

This paper presents in-depth training tutorials for the NMSM Pipeline that guide users through real-life research to design treatments for two clinical conditions that impair walking function. These tutorials are the first to take users through the entire computational treatment design process, starting with model personalization and ending with treatment optimization using the personalized model. The first tutorial explores personalized joint and foot-ground contact modeling for a single subject with bilateral medial knee osteoarthritis (OA). The tutorial guides users through the model personalization and treatment optimization processes without muscles or neural control to design either a walking modification or high tibial osteotomy (HTO) surgery to reduce the peak adduction moment in both knees. The second tutorial explores personalized muscle-tendon and neural control modeling for a single subject with stroke. The tutorial guides users through the model personalization and treatment optimization processes including muscles and neural control to design a synergy-based functional electrical stimulation (FES) prescription to correct gait propulsion asymmetries. The two tutorials serve as complementary guides to using the NMSM Pipeline for research and give extra focus to the minute details that often affect the quality of predictive simulation results. The tutorials were deployed as two projects in a combined undergraduate/graduate course to demonstrate their effectiveness at teaching personalized neuromusculoskeletal modeling and treatment optimization to novice users.

Full tutorial documents are available as supplemental materials to this article and are also available at nmsm.rice.edu. Readers are encouraged to download the tutorial documents to read them in parallel to this article.

## Methods

### 1. Tutorial Overviews

The tutorials presented in this paper walk users through the model personalization and treatment optimization processes to design personalized treatments for two clinical conditions using the NMSM Pipeline and OpenSim (44,45). Each tutorial focuses on designing a clinical treatment for a single subject with gait dysfunction. The first tutorial explores skeletal modeling for one subject with bilateral medial knee osteoarthritis (OA) (male, age 47 years, height 1.7 m, mass 72.8 kg), while the second tutorial explores neuromusculoskeletal modeling for one subject who suffered a stroke (male, age 79 years, height 1.7 m, mass 80.5 kg; right sided hemiparesis, LE Fugl-Meyer Motor Assessment 32/34 pts).

The experimental walking data used to develop both tutorials were collected from the two subjects under similar conditions. For both subjects, ground reaction data were collected using a split-belt instrumented treadmill (Bertec Corp., Columbus, OH, USA) while the subjects walked at a self-selected speed (1.2 m/s for the subject with knee OA) or fastest comfortable speed (0.8 m/s for the subject with a stroke). Also for both subjects, surface marker motion data were collected using a video-based motion capture system (Vicon Corp., Oxford, UK) with a full-body marker set that included three markers on the pelvis and on each thigh, shank, and foot. In addition, for the subject who had a stroke, 16 channels of surface and fine wire EMG data were collected using two EMG systems (Motion Lab Systems, Baton Rouge, LA, USA). Informed consent was obtained from both subjects, and experimental data collection was approved by the institutional review board of the University of Florida. Gait data collected from these two subjects has been described in previous publications (23,58,60,61).

#### a. Clinical Problems

The first tutorial involves designing a walking modification or HTO surgical procedure to treat a person with bilateral medial knee OA. The peak knee adduction moment (KAM) during stance phase has been proposed as a surrogate measure for medial knee contact force, with a value less than 2.5% BW x HT corresponding to the best long-term outcome for individuals with medial knee OA (62–64). For this subject, the target value for the peak KAM is 30.3 Nm. The peak KAM is derived entirely from external forces and skeletal kinematics and thus is a convenient measure for physics-based predictive simulations that do not calculate internal knee contact forces. Two potential treatments for medial knee OA that can reduce the peak KAM are high tibial osteotomy (HTO) surgery and walking modifications (62,64). For the first treatment, a simulated HTO will approximate the surgery by changing the knee adduction angle of both legs in a predictive simulation, and evaluating the peak KAM produced by the resulting walking motion. For the second treatment, gait modifications, referred to as *medial thrust gait* (MTG), will be predicted using a predictive simulation formulation similar to (21) that includes path constraints on foot kinematics and cost terms that lower the peak KAM to the target value.

The second tutorial involves designing a synergy-based FES prescription to treat a person with right-sided hemiparesis caused by a stroke. The subject is high functioning but has an asymmetry in their propulsive and braking impulses between their legs which leads to an inefficient gait. Synergy-based FES is a novel treatment for stroke that electrically stimulates muscles based on the subject’s neural control characteristics (65–67). Muscle synergies can be calculated that decompose a subject’s muscle EMG data into a lower dimensional set of synergy activations (representing activation timing) and synergy weight vectors (representing activation coordination) (68–71). By comparing a subject’s synergy activations and vectors with those obtained from healthy subjects, researchers have identified a subject’s “impaired” synergies and then stimulated the subject’s paretic leg muscles with patterns consistent with corresponding healthy subject synergies (65,66). For the subject in this tutorial, the calculated muscle synergies shared synergy vectors between the two legs and also made the subject’s personalized model walk the same way the subject did. Calculating bilateral muscle synergies in this unique way allowed direct comparison between the subject’s right and left leg synergies. This comparison revealed asymmetries in the subject’s synergy activation magnitudes but not shapes, allowing each paretic leg synergy activation to be placed into one of three categories: healthy (paretic ~= non-paretic), impaired (paretic < non-paretic), or compensatory (paretic > non-paretic). We hypothesize that correcting the asymmetry in synergy activations will also correct the asymmetry in propulsive and braking impulses. The second tutorial will model a synergy-based FES protocol by scaling matching synergy activations so that they have equal magnitudes between legs. Cost terms will be used to adjust the propulsive and braking impulses so that they are symmetrical as well.

#### b. Tools Descriptions

The primary software tools being used in the tutorials are the NMSM Pipeline’s Model Personalization and Treatment Optimization toolsets (58). The NMSM Pipeline is Matlab-based and interfaces with OpenSim models through OpenSim’s Matlab application programming interface (API). The NMSM Pipeline’s Model Personalization toolset works entirely with Matlab and OpenSim. The NMSM Pipeline’s Treatment Optimization toolset uses the proprietary GPOPS-II direct collocation optimal control solver (72), also in Matlab with OpenSim’s API. The tutorials both use software that is either open-source, widely available in an academic setting, or proprietary but inexpensive

The NMSM Pipeline’s Model Personalization toolset consists of four main tools: Joint Model Personalization (JMP), Ground Contact Model Personalization (GCP), Muscle-tendon Model Personalization (MTP), and Neural Control Model Personalization (NCP). JMP uses marker-motion capture data to calibrate the kinematic model joint parameters to yield more accurate joint angles and joint loads. GCP uses kinematics and six DOF force plate data to calibrate a foot-ground contact model. MTP uses muscle kinematics, EMG data, and joint loads to calibrate Hill-type muscle model parameters (3,22,23) that reproduce experimental joint loads. NCP uses muscle kinematics, muscle activations, and joint loads to fit a muscle synergy-based neural control model that reproduces both muscle activations and joint loads.

The NMSM Pipeline’s Treatment Optimization toolset consists of three main tools: Tracking Optimization (TO), Verification Optimization (VO), and Design Optimization (DO). TO seeks to solve an optimal control problem that spreads out errors in experimental quantities to produce a dynamically consistent walking motion. VO functions as a verification that the TO solution is valid and serves as a “dry run” of DO without any design elements. DO solves an optimal control problem that adjusts the walking motion produced by TO to predict a new motion that optimizes a designated set of design cost terms.

### 2. Tutorial Descriptions

The knee OA tutorial focuses entirely on skeletal modeling, and the stroke tutorial introduced muscles and neural control into the models. Each tutorial has four modules that focus on a different tool in the NMSM Pipeline and include instructions on the relevant OpenSim tools (Table 1). The first two modules of each tutorial focus on the Model Personalization toolset, and the last two modules focus on the Treatment Optimization toolset. OpenSim tools are used throughout the tutorials as relevant to the tutorial material. Each module is designed to take approximately one week to complete, and so some steps are pre-done for users in the tutorial distribution to lower workload and focus on the primary tasks of each tutorial. Detailed instructions are given that are designed to “hold the users’ hand” through the entire modeling process to facilitate active learning and focus on the primary learning objectives of each tutorial. At the end of each module, users are prompted to gather deliverables that are designed to guide them through critically evaluating their results. These deliverables include presentation of raw results, analysis and interpretation of the results, and explanation of the tools being used.

#### a. Tutorial 1 Layout

The first tutorial guides users through using the NMSM Pipeline computational design process for a situation where modeling of muscles and neural control may not be needed and skeletal modeling is sufficient. The tutorial uses the NMSM Pipeline’s JMP, GCP, and torque-driven Treatment Optimization tools, and OpenSim’s Scale Model, and Inverse Kinematics tools to design a walking modification or HTO surgery for a single person with bilateral knee OA. The tutorial consists of four modules, with two Model Personalization modules and two Treatment Optimization modules.

The first module teaches users how to perform the Joint Model Personalization Process. This process involves using the NMSM Pipeline’s JMP tool, and OpenSim’s Scale Model and Inverse Kinematics (IK) tools. The first task of the module guides users through a unique model scaling process that ensures that the foot markers are placed correctly on the feet to help with the subsequent GCP runs. The second task of the module guides users through using the JMP tool to personalize lower extremity functional axes both sequentially in multiple optimizations (i.e., one joint per optimization), and simultaneously in one optimization (i.e., all joints optimized at once). The deliverables for this task include an analysis of IK marker errors for all JMP problem formulations, and an analysis of which JMP problem formulation is optimal for this subject.

The second module teaches users how to perform the Ground Contact Model Personalization Process. This process involves calibrating the subject’s foot-ground contact model using the NMSM Pipeline’s GCP tool and OpenSim’s IK tool. The first task of the module guides users through using the IK tool to calculate joint angles, and then the joint angles are filtered in preparation for GCP. The second task of the module guides users through using the GCP tool to create a personalized foot-ground contact model that closely reproduces experimental ground reaction forces and moments. Users explore two different friction models for the foot-ground contact model: Coulomb friction and viscous friction. The deliverables for this module include an analysis of the experimental data tracking errors for both ground contact models, and a brief explanation of which friction model worked best for this subject.

The third module teaches users how to perform the Tracking Optimization Process with individual joint torque controls. This process involves creating dynamically consistent joint torque-driven walking predictions using the NMSM Pipeline’s Tracking Optimization (TO) tool and OpenSim’s Inverse Dynamics (ID) tool. The first task of the module guides users through using the ID tool to calculate joint loads with their previously calculated and filtered IK joint angles and experimental ground reaction data. The second task of the module guides users through predicting a dynamically consistent torque-driven walking motion using the TO tool. The deliverables for this module include an analysis of the tracking quality for all experimental quantities included in the TO run, and a brief explanation of what quantities have the worst error and how to potentially improve that error.

The fourth module teaches users how to design treatments for walking impairments using the Design Optimization tool with joint torque controls. This process involves using the NMSM Pipeline’s Verification Optimization (VO) and Design Optimization (DO) tools to design either a new MTG walking motion or an HTO surgical intervention that achieves a target value for the subject’s peak KAM in both knees. The first task of the module guides users through using the VO tool to verify that their TO solution from the previous module is valid and compatible with their future DO problem formulation. For the MTG problem, cost and constraint terms are added for foot marker position deviation, foot orientation deviation, and torso orientation deviation to better replicate the results of (21). These cost and constraint terms are added in VO without any design elements to verify that they are consistent with the overall problem formulation. No extra cost or constraint terms are added for the HTO problem. The second task of the module guides users through using the DO tool to design a treatment that achieves a target value for the subject’s peak KAM. This module gives users freedom to design either an MTG motion or an HTO surgery, both with the goal of reducing the subject’s peak KAM to the target value of 2.5% BWxHT for both knees. The design element for the MTG DO is a knee adduction moment minimization cost term for both legs that punished moment values greater than a specified value, where this value is iterated to achieve the target peak KAM value of 2.5% BWxHT in each knee. The design element for the HTO DO is a fixed offset of the knee adduction angle in the model, and then an identical optimization to the previous VO is run with the new model. For both the MTG and HTO problems, users try three different values for the design element and interpolate to find the value that gives the desired target peak KAM for both legs. The deliverables for this module include an analysis of the peak KAM produced by the designed treatment and a brief discussion on the limitations of the predictive simulation to achieve the desired clinical goal.

#### b. Tutorial 2 Layout

The second tutorial guides users through using the NMSM Pipeline computational design process for a situation where modeling of muscles and neural control is clearly needed. The tutorial uses the NMSM Pipeline’s MTP, NCP, and muscle synergy-driven Treatment Optimization tools, and OpenSim’s Muscle Analysis (MA) tool to design a synergy-based FES prescription for a single subject with stroke. The tutorial also consists of four modules, with two Model Personalization modules and two Treatment Optimization modules. The tools used in this tutorial generally take longer to run compared to the first tutorial, and so for some modules, users work through smaller runs on the tools and are given pre-generated results for bigger problems to facilitate data analysis.

The first module teaches users how to perform the Muscle-tendon Model Personalization process. This process involves predicting muscle forces for the subject using the NMSM Pipeline’s MTP tool and Data Preprocessing tool, and OpenSim’s MA tool. The first task of this module focuses on using OpenSim’s MA tool to calculate muscle kinematic quantities that will be used in the MTP optimization. The second task of this module has users work through the NMSM Pipeline’s Data Preprocessing tool to crop, filter, and resample data in preparation for MTP. The third task of this module has users use the NMSM Pipeline’s MTP tool to personalize muscle-tendon parameters to best predict muscle forces, guiding them through an iterative process to obtain the best MTP result. The deliverables for this module include a brief explanation of how the MTP tool works, an analysis of the four iterations students perform, and a brief discussion on why MTP is important for muscle-driven predictive simulations.

The second module teaches users how to perform the Neural Control Model Personalization process. This process involves creating and analyzing a muscle synergy neural control model for the subject using the NMSM Pipeline’s NCP tool. The first task of this module briefly shows users how to format their NCP input data directory after running MTP. The second task of this module guides users through choosing the appropriate number of muscle synergies to use in their NCP run by analyzing the variance accounted for (VAF) of the EMG data as the number of synergies increases. The third task for this module guides users through creating and running an initial NCP settings file. After users run their NCP problem, they are given premade results for data analysis with five synergies and bilaterally symmetric synergy vectors between legs. The deliverables for this module are extensive, and guide users through the full data analysis and diagnosis of the subject’s neural impairment. Users are instructed to analyze the shape of the synergy activations with bilaterally symmetric synergy vectors, identify which synergies are healthy, impaired, or compensatory, and identify the function of each of the synergies. After completing the deliverables, users should have a strong grasp on using NCP to gain insight into a subject’s neural control impairment.

The third module teaches users how to perform the Tracking Optimization process with muscle-synergy controls. This process involves creating a dynamically consistent muscle synergy-driven walking motion using the NMSM Pipeline’s Tracking Optimization and Surrogate Muscle Model tools. The first task of this module guides students through creating a surrogate muscle model using the NMSM Pipeline and OpenSim’s MA tool. The second task of this module guides students through creating and running a synergy-driven Tracking Optimization settings file. Synergy-driven Tracking Optimizations are often difficult to converge to a good solution, and so extra attention is given towards analyzing the convergence of the optimization and how to troubleshoot potential issues. Because synergy-driven TO runs are computationally expensive, the users were only instructed to run one TO problem, and premade results were used for data analysis. The deliverables for this module guides users through the analysis of their TO results and the premade TO results with a mix of quantitative analysis of experimental quantity tracking qualities, and qualitative analysis of the gait motion produced by TO.

The fourth module teaches users how to design treatments for walking impairments using the Design Optimization tool with muscle-synergy controls. This process involves using the NMSM Pipeline’s VO and DO tools to design a synergy-based FES prescription to improve walking symmetry for a person with stroke. The first task of this module guides users through using the VO tool to verify that their TO solution from the previous module is valid and ready to be used in DO. The second task of this module prompts users to plan their FES prescription. The goal for this task is to choose a set of scale factors to apply to the synergy activations such that the magnitude of each synergy activation is equal between legs. The third task of this module guides users through implementing their FES prescription in DO to equalize the propulsive and braking impulses between the subject’s feet. The target propulsive and braking impulses are calculated by averaging the experimental propulsive impulses, and cost terms are used to achieve this value. Additional cost terms are added that track a scaled version of the synergy activations such that their shape stays nearly the same but their magnitude can increase or decrease. It is assumed that users are stimulating only the impaired synergies, and that the subject will be capable of correcting any compensatory synergies in response to the stimulation. Next, users analyze their DO results, explain their rationale for the scale factors they chose, and present the potential limitations of transferring their FES prescription to a clinical setting.

## Results

Simulation results for both tutorials are presented in this section. For simulations that track experimental quantities, root mean squared error (RMSE) values are reported. Iteration counts and computation times for the simulations when run on an AMD Ryzen 9 7950× 16 core processor with 32 GB of DDR5 RAM are presented.

### a. Tutorial 1 Results

**Figure 1:**
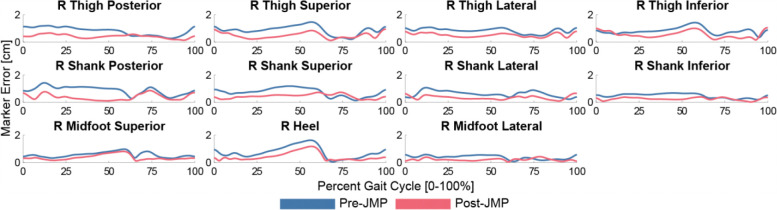
Right leg inverse kinematics marker tracking errors before JMP (blue) and after JMP (red). JMP greatly reduced the average marker tracking errors, and peak errors for all lower limb markers included in the optimization.

The JMP module yields substantially lower marker errors for both the sequential and simultaneous runs. Prior to JMP, IK marker tracking errors during gait are an average of 0.89cm with a max error of 1.8 cm. The sequential JMP run reduces the average marker tracking error to 0.65 cm and the max error to 1.6 cm. The simultaneous JMP run reduces the average marker tracking error to 0.57 cm and the max error to 1.6 cm (Figure 1). The simultaneous JMP run produces the lowest marker tracking error, and so is used for the remainder of the tutorial. This JMP run is terminated after 6 iterations and 0.37 Hours.

**Figure 2:**
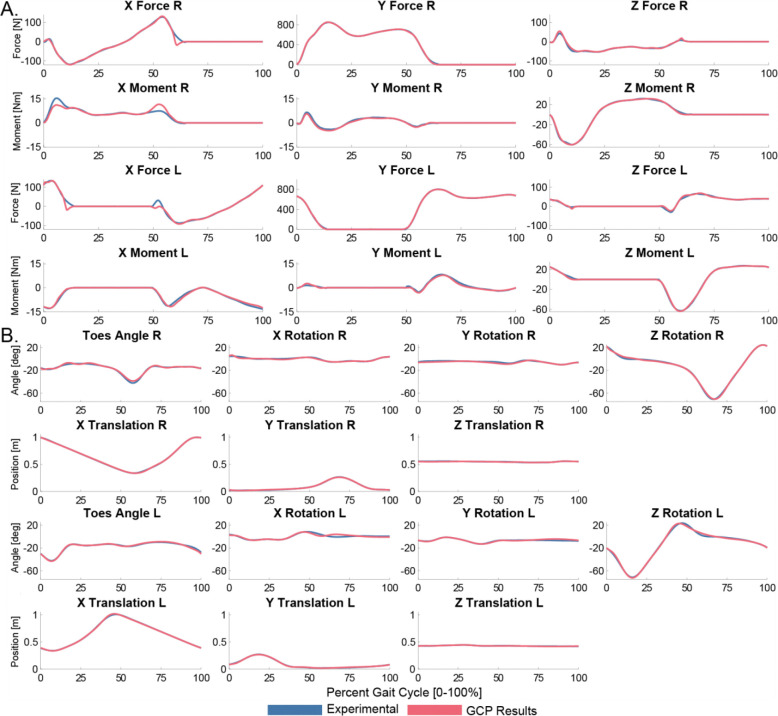
Ground reaction forces and moments (A), and kinematic tracking quality (B) tracking quality from GCP using Coulomb friction. The GCP modeled values (red) closely tracked the experimental ground reactions (blue) with minimal changes in the experimental foot kinematics, indicating a good GCP fit that is consistent with model kinematics.

The GCP module calibrates a foot-ground contact model that can accurately reproduce experimental ground reaction forces and moments with minimal changes in foot kinematics. The GCP model with viscous friction achieves an average ground reaction force tracking RMSE of 5.9 N and an average ground reaction moment tracking RMSE of 0.81 Nm with average calcaneus orientation and translation, and toes angle tracking RMSE of 1.3 degrees, 0.38 cm, and 1.1 degrees, respectively. The GCP model with Coulomb friction achieves an average ground reaction force tracking RMSE of 4.9 N and an average ground reaction moment tracking RMSE of 0.77 Nm with average calcaneus orientation and translation, and toes angle tracking RMSE of 1.2 degrees, 0.37 cm, and 1.1 degrees, respectively (Figure 2). The GCP model with Coulomb friction produces the lowest tracking errors and so is used for the subsequent Treatment Optimization. This GCP run is terminated after 87 iterations and 0.19 hours.

**Figure 3:**
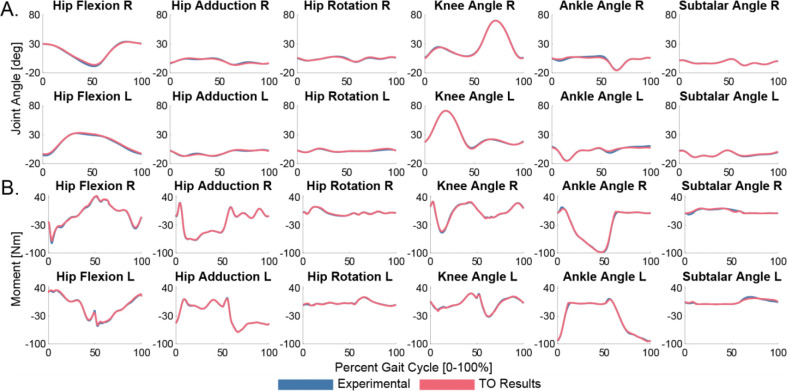
Torque-driven TO results for joint angles (A) and joint loads (B). The TO results (red) closely tracked all lower limb experimental joint angles and loads (blue).

**Figure 4:**
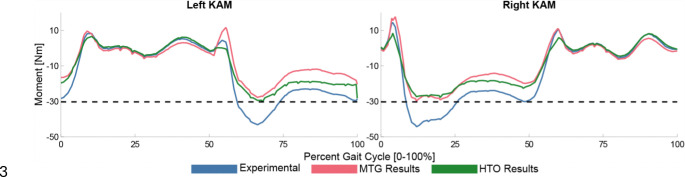
Left and right KAM plots for the experimental (blue), HTO (red), and MTG (green) motions. Both the HTO and MTG simulations produced a peak KAM below the target value for this subject of 30.3 Nm (black dotted).

The TO module produces a dynamically consistent walking motion that closely tracks all joint angles, joint loads, and ground reactions. All rotational joint angles are tracked to within 1.8 degrees RMSE, and all translational joint positions are tracked to within 0.29 cm RMSE. All lower limb joint loads are tracked to within 2.0Nm. (Figure 3) The peak KAM for the right and left legs are −44.3 Nm and −43.15 Nm, respectively (Figure 4). Ground reaction forces are all tracked to within 9 N RMSE, and ground reaction moments are all tracked to within 2.1 Nm RMSE. This TO run converges in 95 iterations and 0.19 hours. The VO results closely tracked the TO solution with negligible RMSE across all quantities, thus verifying that the TO solution is valid and ready for DO. The HTO VO run converges in 3 iterations and 0.02 hours and the MTG VO run converges in 4 iterations and 0.02 hours.

The MTG and HTO DO runs are both able to reduce the peak KAM to below the desired value of 30.3 Nm (Figure 4). For the HTO problem, corrections of 3 degrees, 6 degrees, and 9 degrees are tested, and ~6 degrees is found to be the optimal correction for this subject. For the MTG problem, max allowable errors of 10 Nm, 15 Nm, and 20 Nm are tested, and ~15 Nm is found to be the optimal value for this subject. The HTO DO run converges in 60 iterations and 0.12 hours.

### b. Tutorial 2 Results

**Figure 5:**
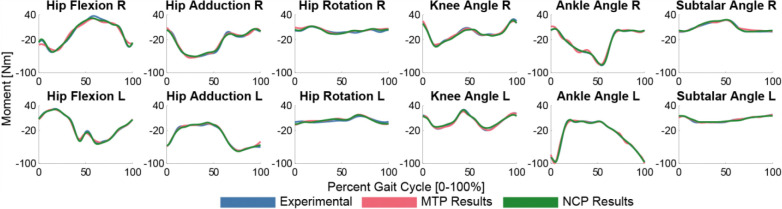
MTP (red) and NCP (green) tracking quality of experimental joint moments (blue). Both MTP and NCP closely tracked the experimental joint moments, and NCP created a smoothing effect on the joint moments that produced better experimental moment tracking.

The MTP and NCP modules yield accurate joint moment matching results for the final runs that are used in TO. After all the iterations, the MTP calibrated muscle model produces joint moment matching with RMSE within 5.1 Nm and max absolute errors within 4.5 Nm (Figure 5). This MTP run converges in 508 iterations and 0.08 hours. The NCP muscle synergy model with bilateral symmetry produces joint moment matching with RMSE within 3.2 Nm and represents the muscle activations with a total percent VAF of 90.97% with a worse individual muscle RMSE of 0.092. This NCP run converges in 641 iterations and 0.55 hours.

**Figure 6:**
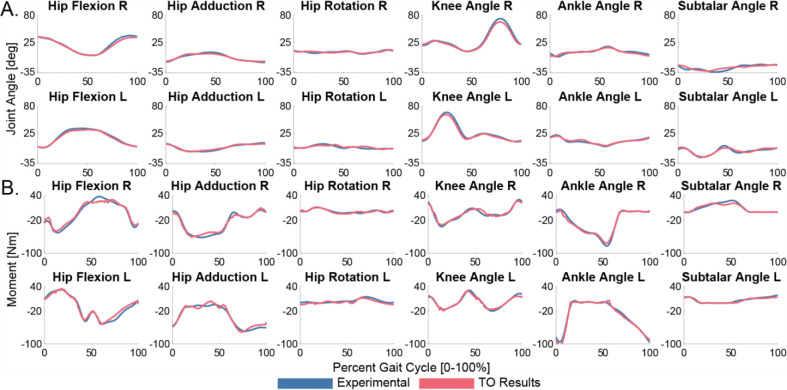
Synergy-driven TO tracking results for joint angles (A) and joint moments (B). The TO results (red) closely tracked all lower limb experimental joint angles and loads (blue).

The TO module produces a dynamically consistent synergy-driven walking motion that closely tracks all joint angles, joint loads, ground reactions, and muscle activations. All lower limb rotational joint angles are tracked to within 3.2 degrees RMSE, and all translational joint positions are tracked to within 0.57 cm RMSE. All lower limb joint loads are tracked to within 6.0 Nm (Figure 6). Horizontal ground reaction forces are all tracked to within 8.2 N RMSE, the vertical ground reaction forces are tracked to within 27 N RMSE, and ground reaction moments are all tracked to within 2.6 Nm RMSE. All muscle activations are tracked to within 0.075 RMSE. This TO run converges in 270 iterations and 6.25 hours. The VO results closely track the TO solution with negligible RMSE across all quantities, thus verifying that the TO solution is valid and ready for DO. This VO run converges in 55 iterations and 0.28 hours.

**Figure 7:**
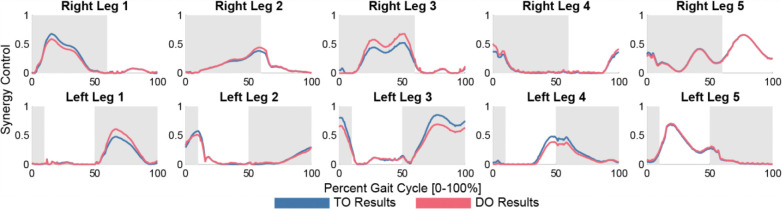
TO (blue) and scaled DO (red) synergy activations throughout a gait cycle. The stance phases for each leg are shaded. Synergy activations were scaled so that each activation had a magnitude equal to the average magnitude pre-treatment.

The synergy-driven DO run with cost terms for synergy activation scaling and propulsive and braking impulse targets can equalize the propulsive and braking impulses to within 5% error of each other (Table 2). Results are presented for a synergy activation scaling strategy in which the right and left leg synergy activations are scaled up or down to their average magnitude (Figure 7). This DO run converges in 229 iterations and 1.10 hours.

## Discussion

This paper presented a set of neuromusculoskeletal modeling tutorials using the NMSM Pipeline and OpenSim that go deep into the details of **how** to approach the computational treatment design process. Extra attention is given to “small” details that can make or break an entire research project, and yet these details are rarely presented in traditional journal publications. It is valuable to have a smaller number of methods-focused journal articles to ease the learning curve of getting into the biomechanical gait modeling field. Currently, most researchers can learn how to use tools such as OpenSim through a set of tutorials that explore the main functionality of the tools but lack explanation of the finer details that are critical for research tutorials. While extensive details are useful, including them makes tutorial development much harder and time consuming. Development of the in-depth tutorials presented here took three individuals a year and a half, which is not realistic for every software tutorial. These tutorials are based on real research first, and then the instructions were written after results were obtained. This process required working through the simulation pipeline numerous times to verify that the instructions work as intended and to test different scenarios that could impact optimization convergence. The result is a set of comprehensive tutorial instructions for how to approach computational treatment design and how to use the NMSM Pipeline for this purpose.

The knee OA tutorial walks users through successfully reproducing the validated results in (62) or in predicting an HTO surgery. Reproducing the result in (21) required a specific problem formulation in which foot position and orientation are constrained because the original paper did not use a ground contact model but rather prescribed the motion of the feet with respect to ground and applied slightly variable ground reactions to them. The HTO solution is not validated, but the optimal correction of 6 degrees falls within the typical range of correction for HTO (73).

The two knee OA treatment predictions have limitations that may affect their prediction accuracy. First, the HTO surgery was simulated by simply changing the knee adduction angle. However, in reality the surgery adjusts the angle of the tibia below the knee joint. This limitation could be addressed by adding a specific HTO joint in the tibia that anatomically represents the surgery. Next, the surgery simulation did not use muscles, which could result in the simulations predicting a motion that the subject’s neural control could not achieve. While the medial thrust gait has been validated for a single subject (21), other subjects with medial knee OA involved in a small pilot study were unable to achieve it. A potential explanation is that the current predictions do account for subject-specific neural control limitations. Therefore, it is possible that including a personalized neural control model into these predictive simulations could yield more accurate results for some subjects. Nevertheless, both the HTO and MTG simulations as performed in the tutorials produce physiologically realistic results.

The stroke tutorial successfully walks users through designing a synergy-based FES pattern to correct gait asymmetry using a Design Optimization. The treatment design in the tutorial relies on several key assumptions that are not common in the literature. First, the analysis assumed that synergy vectors are symmetrical between legs. This assumption relies on the hypothesis that synergy activations are sourced from the brain, and synergy vectors are sourced from the spinal cord (74,75). A stroke doesn’t affect the spinal cord; therefore this hypothesis would imply that synergy vectors should be symmetrical even after a stroke. However, there are also claims that stroke can cause synergies to “merge” which would change synergy vectors (76). By imposing that the synergy vectors are symmetrical, we can compare corresponding synergy activations and observe that the shapes of the synergy activations were similar between legs, but with different magnitudes. We assume that the synergies can be classified as impaired, compensatory, or healthy based only on their magnitudes (65,66). Furthermore, we assume that when the subject has their impaired synergies stimulated, they are able to correct their compensatory synergies back to healthy levels, which might not be possible depending on how severe the neural control impairment is. Another limitation with the method for simulating FES is that most clinical setups can only programmatically stimulate up to eight total muscles (65,66,77) which makes stimulating an entire synergy in the way that is done in the tutorial unrealistic. In a clinical setting, the primary muscles in the impaired synergies would need to be picked out the synergies and stimulated. These limitations are significant and highlight that the synergy-based FES treatments designed with this computational method need to be validated experimentally validated.

The stroke tutorial worked through the model personalization and treatment optimization process with the same dataset that was used in the NMSM Pipeline article but with substantial differences (58). Firstly, the OpenSim model used in the present tutorial was updated from the original article, featuring an updated subtalar joint, knee joint kinematics, knee muscle geometry paths and model muscle model parameters (78), and muscle moment arms for hip adductor muscles (79). The JMP tool for this article personalized the knee, ankle, and subtalar joints simultaneously using functional joint trials and a gait trial, as opposed to (58) which only personalized the knee and ankle joints using a single gait trial. The GCP tool was re-run using a Coulomb friction model and corrected the electrical center of the force plates relative to the lab coordinate system, neither of which were done in the original article. The MTP tool was re-run with the new JMP results, and new OpenSim model. The iterative process to achieve good MTP results was documented as well, providing more insight than the NMSM Pipeline article did into how to get the best MTP results. The NCP tool was re-run with 5 muscle synergies per leg instead of 6 like in the NMSM Pipeline article. Finally, the Design Optimization process was formulated significantly differently. The NMSM Pipeline article used the DO tool to achieve a target value of metabolic cost and equalized muscle synergies using a complex user defined cost term. The present tutorial aimed to equalize the propulsive and braking impulses between the subject’s feet to achieve a more symmetric gait by simply scaling synergy activation magnitudes to the average value between legs. When users work through the tutorial, they are also given complete freedom for how to scale the synergies so that they can compare the differences between equalization methods.

The methods in these tutorials are formulated to work for these specific research problems with these specific subjects. They are not guaranteed to work for general problems and should not be taken as such. Rather, the goal of the tutorials is to teach users how to use the full breadth of NMSM Pipeline functionality for advanced problems that will be encountered in research. Attention is given to **how** settings files are created, with details on each individual setting, and **why** settings files options are used so that users can extrapolate that reasoning to their tutorials. However, the specific analyses used are subjective and formulated specifically for these subjects. These tutorials give examples of using neuromusculoskeletal models to diagnose impairments and design treatments, but as with most clinical problems, subjective decision making is still an important part of the treatment design process.

To test these tutorials in a real-world environment, we had students complete them for a combined undergraduate/graduate mechanical engineering course. No prerequisite biomechanics, numerical methods, or coding knowledge was required or expected. The in-class lectures were structured around basic musculoskeletal modeling concepts and these tutorials, with each module having two corresponding lectures: a theoretical lecture covering how the relevant tools and modeling techniques work, and a hands-on lecture that worked through simplified tutorials with students to demonstrate how to use the relevant OpenSim and NMSM Pipeline tools. The theoretical lectures were designed to teach the “what” and the “why” of neuromusculoskeletal modeling. The lectures focused on the history of modeling techniques, how the current tools improve on older tools, and why the modeling techniques being discussed are important to the field of neuromusculoskeletal modeling. The hands-on lectures were designed to teach the “how” of neuromusculoskeletal modeling and interactively worked through basic tutorials for the NMSM Pipeline tools and OpenSim tools. Students were encouraged to work interactively through the tutorials during the lectures. All lectures were recorded and uploaded to the course website for students to review later. All modules were assigned to students on the day of the hands-on lecture. To ensure that students struggling with previous modules could complete future modules, premade results from previous modules were released to students each time a new module was assigned. Numerous resources were given outside of class to support students working through the tutorials on their own time, including frequently asked questions (FAQ) boards, a computer lab to run the software, and regular office hours. Every time a student emailed the instructor with questions, the questions and answers were anonymized and recorded on the course website. Students were encouraged to check this page whenever they had a problem. Students were given 24/7 access to an on-campus computer lab with all of the necessary software pre-installed on all computers. The Windows computers were well equipped with 20 core CPUs and 32 GB of RAM and so could run all NMSM Pipeline tools reasonably fast.

Students were able to work through both tutorials successfully and achieve the desired final results. While the course projects were successful and achieved overall good feedback from students, several important limitations were revealed from troubles that students had. First and foremost, even with the level of detailed instructions, students often struggled to get the NMSM Pipeline tools running because they missed one of the steps. Often times, these user errors resulted in Matlab errors that were difficult for students to decipher. Students’ only option was to talk to the course instructor or teaching assistant that had extensive experience with the NMSM Pipeline. This limitation can be addressed by adding more robust error catching code to the NMSM Pipeline so that errors are caught before runtime and give actionable feedback to users. Another limitation concerns the computational load of the tutorials. The JMP, NCP, and Treatment Optimization tools all have the potential to take over an hour of CPU time to converge. The students were given access to a computer lab that could run these optimizations, but the optimization runtime was still a concern for students. Future improvements to the NMSM Pipeline could speed up the runtimes, but until then, pre-made results need to be given to students for some modules to alleviate the time required to complete the tutorials.

One potential area for improvement that students recommended was to add more exploration to the tutorials. The goal of the instruction documents is that if users follow the steps in the document, they will get the exact same results as we did when developing the tutorials. This goal requires exceptionally detailed instructions with little room for interpretation. For every tool, the exact settings that users need to fill out are explicitly given in the instructions. Users are never required to deduce what the correct settings should be to get the correct results. All areas for potential exploration are thus planned and designed in a way that users are not likely to struggle with simply getting the tools to run. These areas for exploration are presented at the end of the tutorials once users should already have a grasp of how to make settings files and run the tools. There is a fine balance between providing adequate detail so that users do not struggle to get the tools to work properly and giving enough opportunities for exploration so that users feel that they have agency when working through the tutorials. A potential future area of improvement for these tutorials is to increase the opportunities user exploration, but it is important to make the NMSM Pipeline easier to use before users are expected to explore on their own accord.

## Conclusions

The detailed tutorials presented here serve as an introduction to using personalized predictive gait simulations to design subject-specific treatments for movement impairments using the NMSM Pipeline. The NMSM Pipeline has made predictive simulations and computational treatment design using personalized neuromusculoskeletal models more accessible than was previously possible, enabling these tutorials to be developed. The key takeaways from these tutorials are a working knowledge of using personalized neuromusculoskeletal modeling techniques within the NMSM Pipeline and OpenSim to design subject-specific treatments for gait dysfunction. These tutorials are accessible to beginning users of the NMSM Pipeline and do not require any coding knowledge, making them valuable for both clinicians and newcomers to the biomechanics field. We hope that by working through these tutorials, users will develop an enhanced understanding of the NMSM Pipeline and will be able to apply the NMSM Pipeline’s tools to their own research and clinical problems. The full tutorial distributions can be found in the supplemental materials of this publication and on the NMSM Pipeline website nmsm.rice.edu. Tutorial materials on nmsm.rice.edu will be updated periodically to keep up with new functionality as new features for the NMSM Pipeline are released.

## Supplementary Material

Supplement 1

Supplement 2

Supplementary Information

The tutorials described in this article can be viewed at nmsm.rice.edu, and in the online version of this article.

## Figures and Tables

**Table 1:** List of NMSM Pipeline and OpenSim tools that are used in each tutorial.

Toolset	Tool	Tutorial 1	Tutorial 2

NMSM Pipeline Model Personalization	Joint Model Personalization	Performed	Provided
	Ground Contact Personalization	Performed	Provided
	Muscle-tendon Personalization	N/A	Performed
	Neural Control Personalization	N/A	Performed

NMSM Pipeline Treatment Optimization	Tracking Optimization	Performed	Performed
	Verification Optimization	Performed	Performed
	Design Optimization	Performed	Performed

OpenSim	Scale Model	Performed	Provided
	Inverse Kinematics	Performed	Provided
	Inverse Dynamics	Performed	Provided
	Muscle Analysis	N/A	Performed

For tools labeled Performed, users specifically work through that tool in the tutorial. For tools labeled Provided, pre-generated results are provided to users.

**Table 2:** Propulsive and braking impulses before and after DO.

		Propulsive Impulse	Braking Impulse

TO Results	Right Leg	5.92	15.64
	Left Leg	18.42	5.35

DO Results	Right Leg	10.77	10.80
	Left Leg	10.84	10.69

The DO problem could equalize the propulsive and braking impulses to within 0.8 Ns of the target value of 10.77 Ns.

## Data Availability

The NMSM Pipeline software, along with the tutorial documents, data, models, and settings files described in this article, are available from the “Neuromusculoskeletal Modeling (NMSM) Pipeline” project on SimTK.org (https://simtk.org/projects/nmsm). The original tutorials corresponding to this article are available under the “JNER Reproducible Research Using the NMSM Pipeline” download on SimTK. The tutorials will be updated over time and can be downloaded under the “NMSM Advanced Tutorials” download on SimTK, and on nmsm.rice.edu. Users are welcome to ask questions, share feedback, and request new functionality through this project’s forum. Documentation for the NMSM Pipeline including implementation details, examples, and tutorials is available at nmsm.rice.edu.

## List of abbreviations

NMSM: Neuromusculoskeletal modeling
NMS: Neuromusculoskeletal
JMP: Joint Model Personalization
MTP: Muscle-tendon Model Personalization
NCP: Neural Control Model Personalization
GCP: Ground Contact Model Personalization
TO: Tracking Optimization
VO: Verification Optimization
DO: Design Optimization
IK: Inverse Kinematics
ID: Inverse Dynamics
MA: Muscle Analysis
OA: Osteoarthritis
HTO: High Tibial Osteotomy
MTG: Medial Thrust Gait
KAM: Knee Adduction Moment
EMG: Electromyography
FES: Functional Electrical Stimulation
API: Application Programming Interface
VAF: Variance Accounted For
RMSE: Root Mean Squared Error
