# Supplementary material for: Reproducible Research: Computational Design of Personalized Clinical Treatments for Walking Impairments Using the Neuromusculoskeletal Modeling Pipeline
Source: bioRxiv. 2026 Mar 4:2026.03.02.709099. Preprint. [Version 1] doi: 10.64898/2026.03.02.709099 (PMC12991138; doi:10.64898/2026.03.02.709099)
Supplement: Supplement 1 [file media-1.pdf]

TORQUE-DRIVEN SKELETAL MODEL TREATMENT OPTIMIZATION
NMSM Pipeline Advanced Tutorial 1

Tutorial Developer: B.J. Fregly, Rice Computational Neuromechanics Lab, Rice University

Simulation Project Materials

The materials for this tutorial can be downloaded on SimTK at https://simtk.org/projects/nmsm under the "NMSM Advanced Tutorials" download.

Simulation Project Overview

The goal of this simulation project is to teach you the Neuromusculoskeletal Modeling (NMSM) Pipeline's computational treatment design process for clinical applications (Fregly, 2021; Hammond *et al*., 2025) where use of a personalized torque-driven skeletal model would be sufficient and modeling of muscles or neural control would not be needed. Specifically for this project, you will a) develop a personalized torque-driven three-dimensional skeletal model and then b) use the personalized model to design a modified walking motion as well as a high tibial osteotomy surgical plan to reduce both peak adduction moment peaks for a subject with bilateral medial compartment knee osteoarthritis. This project will involve similar tasks to those performed by Prof. Fregly in his 2007 journal article "Design of Patient-specific Gait Modifications for Knee Osteoarthritis Rehabilitation" (Fregly *et al*., 2007), where the peak knee adduction moment was used as a surrogate measure for medial knee contact force. The specific treatment design goals for the modified walking motion and high tibial osteotomy surgery are provided in the project details below.

The five tools that you will use within the NMSM Pipeline are indicated in the table below, along with the abbreviations used to reference each tool and required supporting OpenSim tools:

NMSM Pipeline Toolset	NMSM Pipeline Tool	Used
Model Personalization	Joint Model Personalization (JMP)<br>(with OpenSim Scale Model tool)	✓
	Muscle-tendon Model Personalization (MTP)	
	Neural Control Model Personalization (NCP)	
	Ground Contact Model Personalization (GCP)<br>(with OpenSim Inverse Kinematics tool)	✓
Treatment Optimization	Tracking Optimization (TO)<br>(with OpenSim Inverse Dynamics tool)	✓
	Verification Optimization (VO)	✓
	Design Optimization (DO)	✓

For the **Model Personalization** toolset, since this simulation lab will require only a personalized skeletal model without including personalized models of muscle-tendon actuators or neural control, you will need only the **Joint Model Personalization** tool and the **Ground Contact Model**

Personalization tool. In contrast, for the **Treatment Optimization** toolset, you will still need all three available tools, where your personalized model will be controlled by torque actuators due to the omission of muscles.
This simulation project is broken down into three modules, one for each **Model Personalization** tool and a third for all three **Treatment Optimization** tools. For each module, detailed instructions are provided below to walk you through all the necessary steps. To ensure that poor results for one module do not affect your ability to complete subsequent modules, final results for each module are provided to use as the starting point for the next module if necessary.

To run each required OpenSim or NMSM Pipeline tool, you will generate an initial `xml` settings file using the appropriate tool selection within the OpenSim GUI Tools menu. Once you have generated an initial tool settings file in the OpenSim GUI, you can edit the settings file for subsequent tool runs either within the OpenSim GUI or using a text editor. Runs for OpenSim tools will be performed through the OpenSim GUI, while runs for NMSM Pipeline tools will be performed in Matlab.

The full-body OpenSim model `Full_Body_Walking_Model.osim` that you will use for this project contains slightly modified knee joint models. Instead of having the knee joint connect the femur body to the tibia body, the knee joint in the slightly modified model connects the femur body to a non-standard proximal tibia body. The proximal tibia body is then connected to the tibia body via a custom joint that allows only X axis rotation, which is locked. OpenSim can calculate inverse dynamics loads only about joint axes present in the model. Since the knee adduction moment should be calculated about the X axis of the tibia body, adding this locked custom joint provides the correct joint axis direction for calculating the adduction moment for each knee.

All experimental marker motion and ground reaction data needed to complete this simulation project have been pre-processed for you and are ready to use without further modification. The experimental data that you will need for this project come from the following trials summarized below:
- Trial04_Static – standing static trial
- Trial06_AnkleR – isolated right ankle motion trial
- Trial07_KneeR – isolated right knee motion trial
- Trial08_HipR – isolated right hip motion trial
- Trial09_AnkleL – isolated left ankle motion trial
- Trial10_KneeL – isolated left knee motion trial
- Trial11_HipL – isolated left hip motion trial
- Trial12_Gait – walking trial collected at self-selected speed of 1.2 m/s

Marker motion data were collected using a Vicon video-based motion capture system (Vicon Corporation, Oxford, United Kingdom), while ground reaction data were collected using a Bertec split-belt instrumented treadmill (Bertec Corporation, Columbus, OH, United States) with belts tied to the same speed. The experimental data from each trial above have already been converted so that all length data are in units of meters, all moment data are in units of Newton-meters, and all data are reported using coordinate axes consistent with OpenSim model conventions (i.e., +X is directed anteriorly, +Y is directed superiorly, and +Z is directed out to the right).

The specific datafiles that you need to complete the project are organized for you in the `Data`

folder. For each OpenSim or NMSM Pipeline tool run that you need to perform, a sub-directory within the `Data` folder is provided containing all of the pre-processed data that you will need. For example, for the OpenSim **Scale Model** tool run described below, all necessary data are provided in the `Scale Model` folder.

MODULE 1: JOINT MODEL PERSONALIZTION

In this module, you will used the OpenSim Scale Model tool and the NMSM Pipeline **Joint Model Personalization** (JMP) tool to personalize lower body joint functional axis positions and orientations in a scaled generic full-body OpenSim model. The personalization process will be performed using marker motion data obtained from the isolated joint motion trials as well as the gait trial.

Module Task 1: Model Scaling

The starting point for **Joint Model Personalization** is always a scaled generic OpenSim model (or, when available, an OpenSim model possessing subject-specific bone models obtained from the subject's imaging data). For the present project, you will scale a generic full-body OpenSim model (Ragagopal *et al*., 2016) modified to improve kinematic modeling of the ankles and knees (van den Bogert *et al*., 1994; Hammond *et al*., 2025) and musculoskeletal geometry modeling of the knees and hips (Lai *et al*., 2017; Ulrich *et al*., 2022). However, to improve the subsequent **Ground Contact Model Personalization** process, you will follow a non-standard model scaling process as described below. The interaction between tool settings, data, and models required to perform this module task is shown in the figure below:

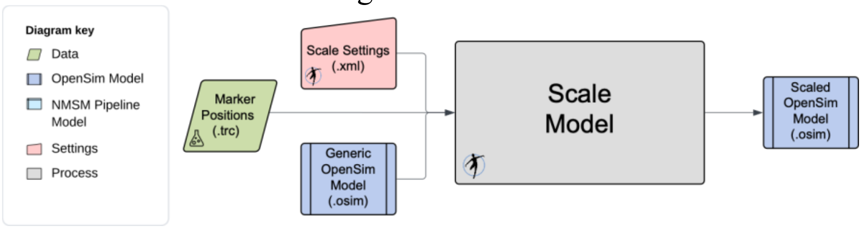

To perform the non-standard model scaling process, you will work with two generic OpenSim models:

1) `Full_Body_Walking_Model.osim` – This model is the generic full-body OpenSim model described above.

   To facilitate the model scaling process, some joints are already locked in the model that are typically unlocked and unlocked other joints in the model that are typically locked, as summarized in the tables below.

   Notable locked joints in the model include the following:

Joint	To Be Unlocked Later	To Remain Locked
`pelvis_list`	✓*	
`knee_adduction_r`		✓
`Knee_adduction_l`		✓
`mtp_angle_r`	✓	
`mtp_angle_l`	✓	
`lumbar_bending`	✓*	
`lumbar_rotation`	✓*	
`pro_sup_r`		✓
`pro_sup_l`		✓

Whether or not the starred (*) joints should be locked depends on the subject being modeled. Locking these joints during the model scaling process helps ensure that the final model pose in the standing static trial is physically realistic for this particular subject (e.g., the pelvis is level when viewed from the front, the torso is not tilted to the side or twisted about a vertical axis).

Notable unlocked joints in the model include the following:

Joint	To Be Locked Later	To Remain Unlocked
`knee_frontal_r`	✓	
`knee_frontal_l`	✓	

The knee frontal angles and the knee adduction angles are similar to each other but serve two different purposes. The knee frontal angles are used in the definition of the knee joints. By unlocking these angles during the model scaling process, we ensure that the static frontal plane alignment of both knees in the model is realistic for the subject (i.e., both knees should end up with a slight varus, or bow-legged, alignment) when the model is in the static pose. Having the correct frontal plane knee alignment in the static pose is important since this pose is used to "glue" the experimental markers onto the model's body segments. If the frontal plane knee alignment is incorrect in the static pose, then the tibial markers will not be placed on the model correctly at the end of the model scaling process. In contrast, the knee adduction angles are used for calculating the knee adduction moments via inverse dynamics. By keeping these angles locked, we create a joint axis that is directed along the X axis of each tibia and that OpenSim can use for calculating a knee adduction moment.

2) `Feet_Only_Walking_Model.osim` – This model contains only the feet from the full-body OpenSim model. This model is needed so that the feet in the model can be properly aligned with the experimental foot marker locations in the static standing trial. If a foot is misaligned with respect to its experimental foot marker locations, then the personalized ground contact model to be developed later for that foot will not work properly.

To construct this model, the following entities are removed from the full-body model:

- All bodies except the right and left calcaneus and toes bodies.
- All joints except the right and left mtp joints.
- All constraints and forces.
- All markers except for markers attached to the calcaneus and toes bodies of each foot.

In addition, following entities are added to the feet-only model:

- Two 6 DOF **Custom Joints** to connect the calcaneus body of each foot to the ground. The rotation sequence for the right calcaneus with respect to ground is defined as (-X, -Y, Z), while the rotation sequence for the left calcaneus with respect to ground is defined as (X, Y, Z). In this way, the same rotations applied to both feet produce anatomically consistent poses for the two feet (e.g., both feet toed out by the same amount). Both X rotations and both Z rotations are locked to zero to prevent each foot from tilting to one side or the other and frontward or backward.
- A **Coordinate Coupler Constraint** to make the Y translation of the left calcaneus the same as that of the right calcaneus. This constraints ensures that both feet are the same

height above the ground in the standing static pose.

The only experimental data needed for the model scaling process is marker data contained in the static trial datafile `Trial04_Static_markers_reordered.trc`. In addition, you will need to know the mass of the subject, which was 72.8 kg based on force plate data collected during the static standing trial.

When iterating the model scaling steps outlined below, you should always select the **Preview static pose (no marker movement)** checkbox at the bottom of the **Scale Tool** menu. This selection will cause the model scaling process to be performed but without replacing markers on the scaled model with the experimental marker locations. You should then select **File** ⇨ **Preview Experimental Data** in the OpenSim GUI to visualize the experimental marker data from the static trial. You can then compare how far the markers on the scaled model are away from the corresponding experimental marker locations.

Step 1: Perform model scaling for the full-body model

- Load the full-body model `Full_Body_Walking_Model.osim` into the OpenSim GUI.
- Select the **Scale Model** tool to scale the full-body model using the static trial datafile `Trial04_Static_markers_reordered.trc`.
- On the main **Scale Tool** menu, make the output model name **Full_Body_Walking_Model-Scaled**, select **Scale Model** and **Adjust Model Markers**, and pick a small time window of roughly 0.1 sec for averaging the static trial marker data. Set the scaled model mass to 72.8 kg, and check **Preserve mass distribution during scale**.
- On the **Scale Factors** tab, create measurements called **PelvisWidth** (for scaling the pelvis body) using the R_ASIS and L_ASIS markers, **FemurLengthR** (for scaling the right femur and patella bodies) using the R_ASIS and R_Knee_Lateral markers, **TibiaLengthR** (for scaling the right tibia body) using the R_Knee_Lateral and R_Ankle_Lateral markers, **FemurLengthL** (for scaling the left femur and patella bodies) using the L_ASIS and L_Knee_Lateral markers, **TibiaLengthL** (for scaling the left tibia body) using the L_Knee_Lateral and L_Ankle_Lateral markers, **FootLength** (for scaling the talus, calcaneus, and toes bodies on both legs) using heel and toe markers on both feet, and **ForearmLength** (for scaling the radius, ulna, and hands on both arms) using the elbow and wrist markers on both arms. You will use different femur and tibia lengths for the two legs since the subject had a slight leg length discrepancy. Do not scale the pretalus body in either leg, since it is a massless intermediate reference frame that only adds a rotational offset as needed to implement the van den Bogert *et al*. (1994) ankle model. Since the shoulder markers where not placed on the shoulder but rather on the straps of a safety harness behind the shoulders, do not use the shoulder markers to create a measurement for estimating torso height or upper arm length. Instead, input manual scale factors for torso body and the humerus bodies, and try different values until your scaled model matches the elbow and wrist marker positions well. You will probably need to make the scale factor for the torso body less than 1 and the scale factor for the two humerus bodies greater than 1.
- On the **Static Pose Weights** tab under **Marker Name**, select markers and associated weights as follows:
  - The heel and toe markers on both feet using a weight of 10 to cause experimental foot marker positions to be matched closely. Do not pick any other foot markers. Note that the

feet will not invert or evert during the scaling process since the subtalar joint axis on both feet has been temporarily locked to a value of 0.
  - All medial and lateral ankle and knee markers using a weight of 1.
  - The two ASIS markers and the central sacral marker using a weight of 10 to cause experimental pelvis marker positions to be matched closely.
  - The chest marker and the two elbow and wrist markers using a weight of 1.

  By matching the foot and pelvis markers closely, we will ensure that the lower body joint angles represent the subject's experimental leg position well. Since the shoulder marker positions are not closely related to the anatomical shoulder locations, do not select the shoulder markers on this tab.
- Run the **Scale Model** tool to create a scaled full-body model. Note that this scaled model will have adjusted mass properties.
- Click on the **Navigator** tab and save the scaled model by right-clicking on it and selecting **Save As**. Use `Full_Body_Walking_Model-Scaled.osim` as the saved model name.

Step 2: Perform model scaling for the feet-only model
- Load the feet-only model `Feet_Only_Walking_Model.osim` into the OpenSim GUI.
- Select the **Scale Model** tool to scale both feet using the static trial datafile `Trial04_Static_markers_reordered.trc`.
- On the main **Scale Tool** menu, make the output model name **Feet_Only_Walking_Model-Scaled**, select **Scale Model** and **Adjust Model Markers**, and pick the same small time window of 0.1 sec for averaging the static trial marker data. Don't worry about the scaled model mass at this point.
- On the **Scale Factors** tab, create a **FootLength** scale factor using the heel and toe markers for both feet, just like for the full-body model.
- On the **Static Pose Weights** tab, select only the heel and toe markers on each foot, use a weight of 1 for the heel markers, and use a weight of 10 for the toe markers. Give the toe markers more weight since we trust their height above the floor more than we trust the height of the heel markers above the floor.
- Run the **Scale Model** tool to create the scaled model with the experimental markers properly attached to each foot.
- Save the scaled model by right-clicking on it and selecting **Save As**. Use `Feet_Only_Walking_Model-Scaled.osim` as the saved model name.

Step 3: Adjust the scaled full-body model for subsequent tasks

Several remaining adjustments must be made to the scaled full-body OpenSim model `Full_Body_Walking_Model-Scaled.osim` before Model Personalization and Treatment Optimization tasks can be performed:
- Copy the scaled OpenSim model `Full_Body_Walking_Model-Scaled.osim` to a new file called `Full_Body_Walking_Model-Scaled_Adjusted.osim`.
- Open model `Full_Body_Walking_Model-Scaled_Adjusted.osim` in a text editor and make the following three changes:
  - Change the model name at the top of the file to `Full_Body_Walking_Model-Scaled_Adjusted`.

  - Correct an OpenSim bug where the `<scale>` field in some `<TransformAxis>` blocks contains a negative number. Search for `<scale>-` in your model and replace with `<scale>` (i.e., minus sign deleted) to eliminate non-physical negative scale factors. This OpenSim bug should be fixed in version 4.6 whenever it is released.
  - Copy the foot marker locations from OpenSim model `Feet_Only_Walking_Model-Scaled.osim` and replace the corresponding foot marker locations in OpenSim model `Full_Body_Walking_Model-Scaled_Adjusted.osim`, which ensures that the foot marker locations in the full-body model are as consistent as possible with foot marker locations when both feet were flat on the ground.
- Load model `Full_Body_Walking_Model-Scaled_Adjusted.osim` into the OpenSim GUI.
- On the **Coordinates** tab, unlock the following locked coordinates in the model:
  - `pelvis_list`
  - `mtp_angle_r`
  - `mtp_angle_l`
  - `lumbar_bending`
  - `lumbar_rotation`
- On the **Coordinates** tab, set the value of the following unlocked coordinates to 0 and then lock them:
  - `knee_frontal_r`
  - `knee_frontal_l`
- Re-save the modified model as `Full_Body_Walking_Model-Scaled_Adjusted.osim`.

Module Task 2: Joint Model Personalization

Now that you have an appropriately scaled full-body walking model with correctly place markers on the body segments (especially the feet), you are ready to personalize the lower body kinematic structure of your model using the NMSM Pipeline's **Joint Model Personalization** tool. The interaction between tool settings, data, and models required to perform this module task is shown in the figure below:

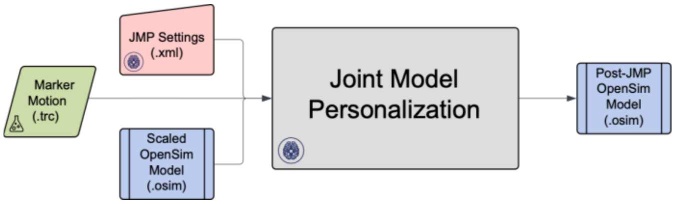

The experimental data needed for the **Joint Model Personalization** process is marker data contained in the isolated joint motion, gait, and combined datafiles located in the `JMP` directory and listed below:

- `Trial06_AnkleR_markers_cropped.trc` – 101 time points of marker data for one cycle of isolated right ankle joint motion
- `Trial07_KneeR_markers_cropped.trc` – 101 time points of marker data for one cycle of isolated right knee motion
- `Trial08_HipR_markers_cropped.trc` – 101 time points of marker data for one cycle of isolated right hip motion
- `Trial09_AnkleL_markers_cropped.trc` – 101 time points of marker data for one cycle of isolated left ankle motion

- `Trial10_KneeL_markers_cropped.trc` – 101 time points of marker data for one cycle of isolated left knee motion
- `Trial11_HipL_markers_cropped.trc` – 101 time points of marker data for one cycle of isolated left hip motion
- `Trial12_Gait_LowerBody_markers_cropped.trc` – 101 time points of marker data for one cycle of reference walking motion
- `Trial04_Static_markers_cropped.trc` – 5 time frames of marker data from the static standing trial
- `Trials06_07_08_LegR_markers_cropped.trc` – 303 time points of concatenated marker data from all isolated joint motion trials for the right leg only
- `Trials09_10_11_LegL_markers_cropped.trc` – 303 time points of concatenated marker data from all isolated joint motion trials for the left leg only
- `Trials06_07_08_09_10_11_BothLegs_markers_cropped.trc` – 606 time points of concatenated marker data from all isolated joint motion trials for both legs
- `Trials08_11_BothHips_markers_cropped.trc` – 202 time points of concatenated marker data from the isolated hip motion trials for both legs
- `Trials_Joints_Gait_markers_cropped.trc` – 707 time points of concatenated marker data from all isolated joint motion trials plus the gait trial

Each datafile contains only lower body markers from the pelvis down, as these markers are the only ones needed to personalize lower body joint functional axes and joint centers. The marker datafile for each type of motion has been splined and cropped to 101 time points representing one complete motion cycle. In this way, each type of motion contributes the same number of marker distance errors to the JMP optimization process when datafiles from multiple types of motions are concatenated into a single combined datafile.

For this module task, you will perform the **Joint Model Personalization** process three times for all lower body joints in the scaled adjusted model by following a three-step process:

Step 1: You will personalize the lower body joints *individually*. In this step, you will perform the personalization process for one (ankle and knee) or two (hips) joints at a time using marker data from the isolated joint motion trials, always running a new JMP task manually starting from the original scaled adjusted OpenSim model. This module step is where you will explore how to formulate an appropriate JMP problem for each type of lower body joint. You will use the knowledge gained in this step to formulate the JMP problems that you will solve in the subsequent two steps.

Step 2: You will personalize the lower body joints *sequentially*. In this step, you will perform the personalization process one joint at time using marker data from the isolated joint motion trials, automatically running a sequence of JMP tasks that always starts from the updated OpenSim model produced by the previous JMP task. The last JMP task in the sequence will utilize the gait trial marker data to personalize all joints together.

Step 3: You will personalize the lower body joints *simultaneously*. In this step, you will perform the personalization process for all joints together using a concatenated datafile containing marker data from all isolated joint motion trials plus the gait trial. Only a single JMP task will be used for this step.

With the sequential approach, improving marker tracking accuracy for one joint (e.g., the knee) could potentially worsen marker tracking accuracy for another joint (e.g., the ankle), but the benefit is reduced computation time compared to the simultaneous approach. Thus, for this module task, you will be investigating the accuracy-speed tradeoff between the sequential and the simultaneous approaches.

For all three steps, you will create a JMP settings file using the **Joint Model Personalization** tool in the OpenSim GUI. For each JMP settings file, you should use the following general guidelines when creating a JMP task:

- Select the marker motion datafile that corresponds to the single joint (ankle or knee), two joint (hips), or multiple joint (gait, or all joints + gait) optimization problem you are trying to solve.
- Accept the default time range so that you will also get one complete motion cycle of marker data possessing 101 time points for each type of motion included in the datafile.
- Select markers from the following list of "dynamic" markers so that markers are present on the parent and child bodies of all joints included in a JMP task (e.g., when personalizing the right knee functional axis, select all markers on the right femur and right tibia):
    - R_ASIS, L_ASIS, and Sacral
    - R_Thigh_Superior, R_Thigh_Inferior, R_Thigh_Lateral, and R_Thigh_Posterior
    - R_Shank_Superior, R_Shank_Inferior, R_Shank_Lateral, and R_Shank_Posterior
    - R_Heel, R_Midfoot_Superior, and R_Midfoot_Lateral
    - L_Thigh_Superior, L_Thigh_Inferior, L_Thigh_Lateral, and L_Thigh_Posterior
    - L_Shank_Superior, L_Shank_Inferior, L_Shank_Lateral, and L_Shank_Posterior
    - L_Heel, L_Midfoot_Superior, and L_Midfoot_Lateral
- Select joints for which parent and/or child frame rotations are to be adjusted, and select bodies for which uniform scaling is to be applied and/or markers are to be moved along selected body axes.
- For each selected joint, do not select any parent or child frame translations. This decision will prevent joints from disarticulating (e.g., the head of the femur will not pull out of the socket in the pelvis).
- For each selected joint, do not select any parent or child frame rotation about the primary axis of rotation for the joint (e.g., the Z axis for the knee). This decision will prevent the optimization from trying to change a joint orientation that is redundant with motion about the primary joint functional axis.
- For each selected joint, keep the default rotation bounds at 0.5, noting that this value is actually in radians and not degrees.
- To personalize an ankle joint, select the ankle joint and corresponding subtalar joint on the desired side, and do not select any bodies. To personalize a knee joint, select the knee joint on the desired side, and do not select any bodies. To personalize both hip joints together, select the pelvis body and allow it to be scaled, and do not select any joints. When personalizing all joints together, consider selecting the tibia and/or femur bodies and allowing both bodies to be scaled and/or markers to be moved along the body segment X and Y directions.
- Never allow markers to be moved on the pelvis, calcaneus, or toes bodies. We trust the marker locations on these bodies (especially on the feet), so allowing them to move is likely to result in an anatomically unrealistic solution.
- To decide which parent and child frame rotations should be changed for which joints, use the

information provided in the table below:

Joint	Functional Axis	Parent Frame Rotation X	Parent Frame Rotation Y	Parent Frame Rotation Z	Child Frame Rotation X	Child Frame Rotation Y	Child Frame Rotation Z
Subtalar	X		✓			✓	✓
Ankle	Z	✓	✓				
Knee	Z	✓	✓		✓	✓	
Hip	N/A						

These parent and child frame rotation changes ensure that the final joint structure remains consistent with the joint's physical anatomy. Feel free to modify these choices if you think that some alternate settings might work better!

- Personalize the orientation of a joint functional axis only if at least ~30 deg of rotation occurs about that axis in the marker data being used for the JMP run (e.g., in the gait trial marker data, the subtalar joint experiences a rotation of < 30 deg, so it's functional axis should not be personalized using gait trial data alone) (Chèze *et al*., 1998).
- For each JMP settings file that you create through the OpenSim GUI, open the settings file in a text editor, increase the value of <function_tolerance> to 1.0e-04, and increase the value of <max_function_evaluations> to 1000. These changes will make your JMP runs converge faster while also allowing enough iterations for tasks that require a large number of function evaluations per iteration.

Minor Bug Note: If you want to read a previously-created JMP settings file back into the OpenSim GUI to modify it, you will need to re-save your settings file with a new name. If you re-save with the same file name, your changes will not be saved in our original settings file.

Once you have created and saved a JMP settings file, you will run the file and generate results by following the instructions below:

- Open Matlab, load the NMSM Pipeline project if necessary, and change directories to where your JMP settings file is located.
- Run the JMP tool in Matlab using the settings file you just saved by inputting the following commands into Matlab:

```
>> parpool
>> tic
>> JointModelPersonalizationTool('JMP_Settings.xml')
>> toc
```

  In this example, the name of the JMP settings file is `JMP_Settings.xml`. The `parpool` command will start a parallel pool of workers (which can take a minute or so) if your computer has multiple cores. The `tic` and `toc` commands will tell you how much wall clock time elapsed between when you started your JMP run and when it finished.
- After completing a JMP run, ALWAYS VISUALIZE YOUR POST-JMP MODEL IN THE OPENSIM GUI!!! When visualizing a post-JMP model, you should not only look at how the model looks in the default pose but also run an OpenSim **Inverse Kinematics** analysis on the post-JMP model using the marker motion `.trc` file used for your JMP optimization. If your settings file allows unrealistic changes in joint positions or orientations, body scaling, or marker locations on the body segments, you may get significantly reduced marker distance errors but an anatomically unrealistic model.

- Plot your pre- and post-JMP marker distance errors using the Matlab function `plotJmpResultsFromSettingsFile.m` as shown below:
  `>> plotJmpResultsFromSettingsFile('JMPSettings.xml')`
  This function will also output the average and maximum marker distance errors for your pre- and post-JMP OpenSim models.

Before performing the three **Joint Model Personalization** steps below, you should review the section describing the **Joint Model Personalization** process in our recently published journal article describing the design and functionality of the NMSM Pipeline (Hammond *et al*., 2025). That section provides helpful information on how to formulate **Joint Model Personalization** problems that reduce marker tracking errors while respecting the anatomic structure of the joints (e.g., ensuring that the "ball" at the head of the femur does not dislocate from the "cup" in the pelvis).

Step 1: Perform joint model personalization for lower body joints individually

- Load the scaled adjusted model `Full_Body_Walking_Model-Scaled_Adjusted.osim` into the OpenSim GUI. NMSM Pipelilne tools will not be accessible in the OpenSim GUI **Tools** menu unless a model to personalize is loaded first.
- Select **Tools** ⇨ **User Plugins** ⇨ **rcnlPlugin.dll** to load the NMSM Pipeline tools into the OpenSim GUI **Tools** menu.
- Select the **Joint Model Personalization** tool to set up one JMP task to be saved as one JMP settings file to personalize one joint (or two joints for the hips) at a time. Repeat this process for the remaining joints, always using the same scaled adjusted OpenSim model as the initial model, and always using isolated joint motion data as the input marker motion data. First, personalize the right ankle by creating a JMP settings file just for the right ankle using the isolated joint motion datafile for the right ankle. Next, personalize the right knee by creating a JMP settings file just for the right knee using the isolated joint motion datafile for the right knee. Finally, personalize both hips together by creating a JMP settings file for both hips using the isolated joint motion datafile for both hips. You do not need to personalize the ankle and knee joints on the left side, since performing the process for just the right side will provide the knowledge you need to perform the next two steps.
- Once a JMP settings file is completed, save it to your hard disk using the **Save** command at the bottom of the tool menu. Name these three JMP settings files `JMP_Settings_RAnkle.xml`, `JMP_SettingsRKnee.xml`, and `JMP_Settings_BothHips.xml`.

Step 2: Perform joint model personalization for lower body joints sequentially

- Load the scaled adjusted model `Full_Body_Walking_Model-Scaled_Adjusted.osim` into the OpenSim GUI. NMSM Pipelilne tools will not be accessible in the OpenSim GUI **Tools** menu unless a model to personalize is loaded first.
- Select **Tools** ⇨ **User Plugins** ⇨ **rcnlPlugin.dll** to load the NMSM Pipeline tools into the OpenSim GUI **Tools** menu (if you have not done so already).
- Select the **Joint Model Personalization** tool to set up a sequence of 6 JMP tasks to be saved as one JMP settings file that personalizes lower body joints one at a time. Each JMP task in the sequence should use the appropriate marker motion data for that task (e.g., the JMP task that personalizes the right ankle should use marker motion data from the isolated right ankle motion trial). As each task in the sequence is completed, the next task will use as its starting

point an updated OpenSim model produced by the previous task. It is up to you to define the JMP task sequence. The only limitation is that the gait trial must be used alone as the final task in the sequence. Possible task sequences from which to choose are listed below:

- AnkleR ⇨ AnkleL ⇨ KneeR ⇨ KneeL ⇨ BothHips ⇨ Gait
- AnkleR ⇨ AnkleL ⇨ BothHips ⇨ KneeR ⇨ KneeL ⇨ Gait
- KneeR ⇨ KneeL ⇨ BothHips ⇨ AnkleR ⇨ AnkleL ⇨ Gait
- KneeR ⇨ KneeL ⇨ AnkleR ⇨ AnkleL ⇨ BothHips ⇨ Gait
- BothHips ⇨ AnkleR ⇨ AnkleL ⇨ KneeR ⇨ KneeL ⇨ Gait
- BothHips ⇨ KneeR ⇨ KneeL ⇨ AnkleR ⇨ AnkleL ⇨ Gait

Select the sequence that you believe has the best chance of producing the lowest marker distance errors. You need to perform the sequential approach for only one sequence!

- Name your output model file `Full_Body_Walking_Model-JMP_Sequential.osim`
- Once your JMP settings file is completed, save it to your hard disk using the **Save** command at the bottom of the tool menu and name it `JMP_Settings_Sequential.xml`.
- Run **Joint Model Personalization** using the instructions provided above.

Step 3: Perform joint model personalization for lower body joints simultaneously

- Load the scaled adjusted model `Full_Body_Walking_Model-Scaled_Adjusted.osim` into the OpenSim GUI.
- Select **Tools** ⇨ **User Plugins** ⇨ **rcnlPlugin.dll** to load the NMSM Pipeline tools into the OpenSim GUI **Tools** menu (if you have not done so already).
- Select the **Joint Model Personalization** tool to set up a single JMP task to be saved as one JMP settings file that personalizes all lower body joints together. The one JMP task should use the marker motion data from each isolated joint motion trial plus the gait trial. In this way, all functional axes of the lower body joints will be exercised within a single datafile. The fact that the motions are discontinuous between one motion and the next is irrelevant, since internally JMP performs repeated OpenSim **Inverse Kinematics** analyses, and these analyses do not require continuity between time frames of marker data.
- Name your output model file `Full_Body_Walking_Model-JMP_Simultaneous.osim`
- Once your JMP settings file is completed, save it to your hard disk using the **Save** command at the bottom of the tool menu and name it `JMP_Settings_Simultaneous.xml`.
- Run **Joint Model Personalization** using the instructions provided above.

Step 4: Select the model with the best personalized joints

- Compare the average and maximum marker distance errors for the gait trial produced by the sequential and simultaneous approaches.
- Identify the approach that produced the lowest errors for the gait trial marker data and that is also the most realistic physically (as determined by visual inspection in the OpenSim GUI – e.g., look for approximate bilateral symmetry in joint coordinate system orientations in the body segments, approximate bilateral symmetry in joint angles in the static pose, reasonable body scale factors, reasonable marker location changes).
- Copy the OpenSim model produced by the "best" approach and give it the new name `Full_Body_Walking_Model-Post_JMP.osim`.
- In a text editor, open the model and change the model name at the top of the file to `Full_Body_Walking_Model-Post_JMP`.

Step 5: Redefine the default pose in preparation for Ground Contact Model Personalization

- Load model `Full_Body_Walking_Model-Post_JMP.osim` into the OpenSim GUI.
- Select the **Inverse Kinematics** tool and run an inverse kinematics analysis using the static trial marker data with the following marker/coordinate selections and weights:
  - Deselect all medial and lateral ankle and knee markers
  - Deselect the non-central sacral markers.
  - Deselect both shoulder markers and the back marker.
  - Select all foot and remaining pelvis markers using a weight of 10.
  - Select all tibia, arm, and chest markers using a weight of 1.
  - Select all thigh markers using a weight of 0.1.
  - Select the `lumbar_rotation` coordinate, leave the **Default Value** as zero, and set the weight to 0.1.

  Note that these weights represent good choices when performing an inverse kinematics analysis for a motion trial. Lightly tracking a `lumbar_rotation` coordinate value of 0 will prevent the torso from twisting unrealistically since a single marker near the midline of the torso is not sufficient to make the `lumbar_rotation` unique.
- Make the static pose the new default pose by selecting the **Coordinates** tab, then **Poses>**, and finally **Set Default**. Making this final static pose the new default pose will place the feet in the correct pose and at the correct height above the floor for contact element placement on the bottom of each foot during the Ground Contact Model Personalization process in the next module.
- Save the model in this final pose by right-clicking on it and selecting **Save**.
- Take a screen shot of the model in the final static pose with the experimental markers shown as well.

Deliverables

1. OpenSim **Scale Model** tool settings file for scaling `Feet_Only_Walking_Model.osim` along with scaled model file `Feet_Only_Walking_Model-Scaled.osim`.
2. OpenSim **Scale Model** tool settings file for scaling `Full_Body_Walking_Model.osim` along with scaled model file `Full_Body_Walking_Model-Scaled.osim`.
3. Scaled adjusted model file `Full_Body_Walking_Model-Scaled_Adjusted.osim`.
4. Screen shot of model `Full_Body_Walking_Model-Scaled_Adjusted.osim` in the static pose found by inverse kinematics with the experimental markers shown as well (screenshot shown below):

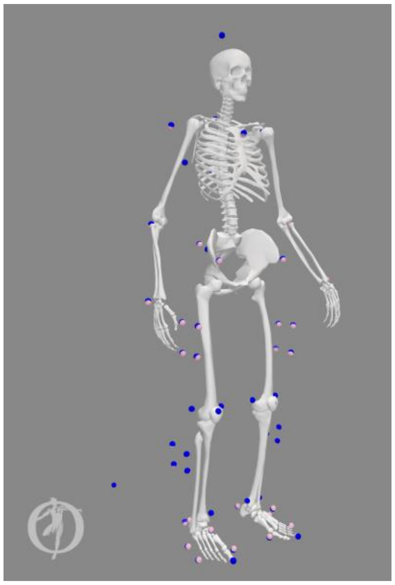

5. Wall clock time along with pre- and post-JMP marker distance errors for each JMP run performed in the three steps above (complete the table below):

JMP Run	Wall Clock Time (hrs)	Pre-JMP Model Avg Error (m)	Pre-JMP Model Max Error (m)	Post-JMP Model Avg Error (m)	Post-JMP Model Max Error (m)
1. Right Ankle					
1. Right Knee					
1. Both Hips					
2. Sequential					
3. Simultaneous					

6. A plot comparing pre- and post-JMP marker distance errors across all time frames for each of the 5 JMP runs shown in the table above.
7. A JMP settings file for each of the 5 JMP runs shown in the table above.
8. Post-JMP OpenSim model `Full_Body_Walking_Model-JMP_Sequential.osim` produced by the sequential approach and `Full_Body_Walking_Model-JMP_Simultaneous.osim` produced by the simultaneous approach.
9. A description of the task sequence that you chose to use for the sequential approach along with a brief argument for why you believe this sequence might produce lower marker distance errors than would other sequences.
10. A brief paragraph explaining when researchers should use the sequential approach and when they should use the simultaneous approach for **Joint Model Personalization**.

MODULE 2: GROUND CONTACT MODEL PERSONALIZTION

In this module, you will use the OpenSim **Inverse Kinematics** tool and the NMSM Pipeline **Ground Contact Model Personalization** (GCP) tool to personalize foot-ground contact model properties in a new NMSM Pipeline model to be associated with your post-JMP OpenSim model. The personalization process will be performed using marker motion and ground reaction data obtained from the gait trial.

Module Task 1: Inverse Kinematics for Gait Motion

The starting point for **Ground Contact Model Personalization** is always a post-JMP OpenSim model whose joint positions/orientations in the body segments, body scale factors, and/or marker locations on the body segments have been calibrated using the **Joint Model Personalization** tool. As indicated above, you will use model `Full_Body_Walking_Model-Post_JMP.osim` as your starting point. You will then perform an OpenSim **Inverse Kinematics** analysis on this model using one cycle of gait trial marker data to generate the input joint motions needed for performing **Ground Contact Model Personalization**. The interaction between tool settings, data, and models required to perform this module task is shown in the figure below:

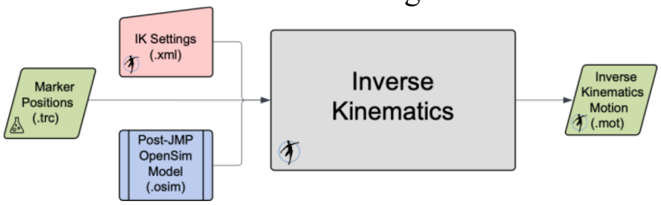

The only experimental data needed for the **Inverse Kinematics** analysis is marker data contained in the gait trial datafile `Trial12_Gait_markers.trc`. This file contains data for all experimental markers, including those on the torso and arms.

Step 1: Perform inverse kinematics using the gait trial marker data.

- Load model `Full_Body_Walking_Model-Post_JMP.osim` into the OpenSim GUI and select the **Inverse Kinematics** tool.
- Under **IK Trial**, select the marker data from gait trial `Trial12_Gait_markers.trc` and keep the default time range from 0 to 4 seconds.
- On the **Weights** tab, select following marker selections and use the following weights:
  - Deselect all medial and lateral toe, ankle, and knee markers
  - Deselect the non-central sacral markers.
  - Deselect both shoulder markers and the back marker.
  - Select all remaining foot and pelvis markers using a weight of 10.
  - Select all tibia, arm, and chest markers using a weight of 1.
  - Select all thigh markers using a weight of 0.1.
  - Select the `lumbar_rotation` coordinate, set a **Manual value** of zero, and set the weight to 0.1.
- Name your **Output Motion File** `Trial12_Gait_IK_results.mot`.
- Once your **Inverse Kinematics** settings file is completed, save it to your hard disk using the **Save** command at the bottom of the tool menu and name it `IK_Settings_Gait.xml`.

- Run your **Inverse Kinematics** analysis and verify that your results file was written to your hard disk. If not, right click on IK Results in the GUI **Navigator** pane and select **Save As** to save the motion file to your hard disk.
- The inverse kinematics results generated in this step cover more than the gait cycle of interest, since the filtering process to be performed in the next step introduces end effects. These effects distort each filtered curve near the start time and end time, causing significant errors in not only the filtered joint position data but also the joint velocity data to be calculated from it. Taking filtered inverse kinematics data from a time window that is not close to the start time or end time eliminates the presence of end effects caused by the filtering process.

Step 2: Filter your inverse kinematics motion file

- Open Matlab and change directories to your `Inverse Kinematics` data folder.
- Run the provided Matlab program `filterIKResults.m` without providing any inputs. The correct default inputs will be used automatically.
- After the program finishes, verify that a new inverse kinematics motion file called `Trial12_Gait_IK_results_filtered.mot` is now present in your `Inverse Kinematics` data folder, and copy this file to your `GCP` data folder.
- Filtered inverse kinematics results are needed since each GCP run will need to differentiate the IK joint position results to generate joint velocity results. These joint velocity estimates are in turn needed to calculate point velocities relative to ground for the contact elements placed on the bottom of each foot by the **Ground Contact Model Personalization** process. Contact element velocities are inputs to vertical contact force nonlinear damping and horizontal contact force friction calculations.
- After generating your filtered inverse kinematics results file, open two versions of model `Full_Body_Walking_Model-Post_JMP.osim` in the OpenSim GUI, load the unfiltered motion into the first model, and load the filtered motion into the second model (it can help to make the second a model a different color). Then sync both motions and animate them together to ensure that the motion of the feet produced by the filtered IK results closely follow the motion of the feet produced by the unfiltered IK results.

Module Task 2: Ground Contact Model Personalization

Now that you have inverse kinematics data available for your walking trial, you are ready to personalize the ground contact model properties of both feet in your model using the NMSM Pipeline's **Ground Contact Model Personalization** tool. You will also need to use filtered experimental ground reaction data for this task. The interaction between tool settings, data, and models required to perform this module task is shown in the figure below:

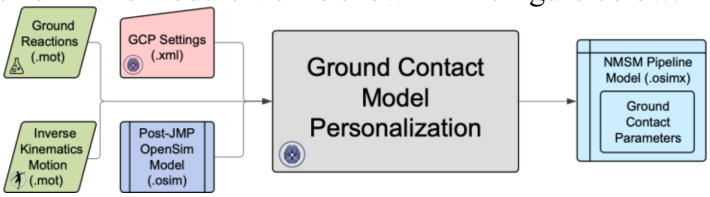

The experimental data needed for the **Ground Contact Model Personalization** process is filtered inverse kinematic joint motion data and filtered ground reaction data contained in the datafiles located in the `GCP` folder and listed below:

- Trial12_Gait_IK_results_filtered.mot – filtered joint motion data for multiple walking cycles.
- Trial12_Gait_forces_filtered.mot – filtered ground reaction data for multiple walking cycles. Note that the sampling frequency for the ground reaction data has been decreased to match the sampling frequency of the marker and inverse kinematics data, which is a requirement for running the GCP tool.

Each datafile contains data for 4 seconds of walking. You will use only a single cycle of walking data, defined by a specified start time and end time, to personalize foot-ground contact models for both feet. Furthermore, the foot-ground contact model personalization process will be performed such that both feet possess the same contact model stiffness, damping, and friction properties.

Note that experimental marker data are not an input to this tool. Instead, your inverse kinematics results generated with tight tracking of foot markers are used as an input. Consequently, GCP tool runs actually track model (rather than experimental) marker trajectories generated by applying your filtered inverse kinematics motion to your OpenSim model.

For this module task, you will perform the **Ground Contact Model Personalization** process two times for both feet together by following a two-step process:

Step 1: You will personalize both feet together assuming Coulomb friction acts on each contact element.

Step 2: You will personalize both feet together assuming viscous friction acts on each contact element.

For both steps, you will create a GCP settings file using the **Ground Contact Model Personalization** tool in the OpenSim GUI. For each GCP settings file, you should use the following general guidelines:

- Load the post-JMP model Full_Body_Walking_Model-Post_JMP.osim into the OpenSim GUI. NMSM Pipelilne tools will not be accessible in the OpenSim GUI **Tools** menu unless a model to personalize is loaded first.
- Select **Tools** ⇨ **User Plugins** ⇨ **rcnlPlugin.dll** to load the NMSM Pipeline tools into the OpenSim GUI **Tools** menu.
- Select the **Ground Contact Model Personalization** tool to set up a sequence of three GCP tasks to be saved as one GCP settings file that personalizes both feet together. Note that you will not create the three GCP tasks in the GUI. A sequence of three default tasks will be created for you automatically when you save your GCP settings file.
- For the **Input Model**, choose your post-JMP model Full_Body_Walking_Model-Post_JMP.osim.
- Leave **Osimx File** blank, since you will be creating a new .osimx file rather than appending to an existing one.
- For **Input Dir**, pick your GCP data folder.
- For **Motion File**, pick Trial12_Gait_IK_results_filtered.mot.
- For **Ground Reaction File**, pick Trial12_Gait_forces_filtered.trc.
- For **Results Dir**, create a folder called gcpResults within your GCP data folder and pick that folder.

- Add two **Ground Contact Personalization Surfaces**, one for the right foot and one for the left foot. For both, use a **Time Range** of 2.235 to 3.280 seconds and a **Belt Speed** of 1.2 m/s. For **Force Columns** (_vx,y,z), **Moment Columns** (_mx,y,z), and **Electrical Center** (_px,y,z), use data from force plate 2 for the right foot and from force plate 1 for the left foot. For **Hindfoot Body**, pick the calcaneus calcn body for the appropriate foot. For various markers, pick the appropriate markers on each foot, noting that **Medial Marker** and **Lateral Marker** represent model markers placed on the toes axis of each foot. For the left foot, make sure to check the **Left Foot** checkbox.
- The midfoot_superior model marker on each foot is a required input for this settings file since the point used for calculating ground reaction moments is the current location of the midfoot superior marker projected onto the floor. Ground reaction moments can be calculated about any desired point, and this choice of point keeps all three components of ground reaction moment "small" for the GCP calibration process. Choice of a point far from the center of the foot would produce large ground reaction moment components, and errors in those components would then swamp matching ground reaction force components during a GCP run.
- Once you have completed these steps in the OpenSim GUI, **Save** your setting file in your GCP folder and name it GCP_Settings.xml. Note that your saved settings file will already have three pre-defined tasks included in it – a first task that focuses on reproducing just the vertical component of ground reaction force for both feet, a second that focuses on reproducing all three components of ground reaction force for both feet, and a third that focuses on reproducing all three components of ground reaction force and all three components of ground reaction moment for both feet. Each of these tasks contains default parameter values that will work well in most cases, and thus users do not have to create the three individual tasks themselves in the OpenSim GUI.
- To finished configuring settings file GCP_Settings.xml that you created in the OpenSim GUI, open it in a text editor (ideally Notepad++ on Windows or BBEdit on Mac) and make the following changes to each <GCPTask>:
    - At the top of the settings file, change the value of <kinematics_filter_cutoff> to 6.
    - For the first task, change the value of <neighborStandardDeviation> to 0.3. For <RCNLCostTerm> items, change <max_allowable_error> for rotation to 0.0175, for ground_reaction_moment to 20, and for neighbor_spring_constant to 1000. Also add a new cost term called kinematic_periodicity and set its <max_allowable_error> to 3, which is a multiple of the ratio between corresponding original initial and final joint positions.
    - For the second task, make the same changes as for the first task plus change <max_allowable_error> for horizontal_grf to 5. Keep <restingSpringLength> set to the default selection of false.
    - For the third task, make the same changes as for the second task and leave <max_allowable_error> for ground_reaction_moment set to 0.5. In addition, set <electricalCenterX> and <electricalCenterZ> to true so that GCP will also calibrate the electrical center location of each force plate in the plane of the force plate.
    - At the bottom of the settings file, where settings are provided that apply to all tasks, change <initial_resting_spring_length> to 0.01, <initial_spring_constant> to 6000, <initial_damping_factor> to 0.5, and <initial_dynamic_friction_coefficient> to 0.3, <diff_min_change> to 1e-4, <step_tolerance> to 1e-5, and <max_iterations> to 25.

Minor Bug Note: If you want to read a previously-created GCP settings file back into the OpenSim GUI to modify it, it will not work. Instead, you will need to modify existing GCP settings files directly using a text editor.

Once you have created and edited a GCP settings file, you will run the file and generate results by following the instructions below:

- Open Matlab, load the NMSM Pipeline project if necessary, and change directories to where your GCP settings file is located.
- Run the GCP tool in Matlab using the settings file you just saved by inputting the following commands into Matlab:
```
>> parpool
>> tic
>> GroundContactPersonalizationTool('GCP_Settings.xml')
>> toc
```
  In this example, the name of the GCP settings file is `GCP_Settings.xml`. The `parpool` command will start a parallel pool of workers (which can take a minute or so) if your computer has multiple cores. The `tic` and `toc` commands will tell you how much wall clock time elapsed between when you started your GCP run and when it finished.
- Plot your GCP results using the Matlab function `plotGcpResultsFromSettingsFile.m` as shown below:
```
>> plotGcpResultsFromSettingsFile('GCP_Settings.xml')
```
  This function will output root-mean-square errors in matching ground reaction forces, ground reaction moments, hindfoot joint translations, hindfoot joint rotations, and toes joint rotations for your two personalized foot-ground contact models, along with a color plot showing the distribution of spring stiffness values over the bottom surface of the foot.

Before performing the two **Ground Contact Model Personalization** steps below, you should review the section describing the **Ground Contact Model Personalization** process in our recently published journal article describing the design and functionality of the NMSM Pipeline (Hammond *et al*., 2025). That section provides helpful information on how to formulate **Ground Contact Model Personalization** problems that reduce match ground reaction forces and moments closely without changing the motion of each foot substantially.

Step 1: Perform ground contact model personalization using Coulomb friction

- Copy the GCP settings file created above and name it `GCP_Settings_Coulomb.xml`.
- Open this file in a text editor.
- Change the name of the `<results_directory>` to `gcpResultsCoulomb`.
- Run **Ground Contact Model Personalization** with this settings file using the instructions provided above.

Step 2: Perform ground contact model personalization using viscous friction

- Copy the original GCP settings file created above and name it `GCP_Settings_Viscous.xml`.
- Open this file in a text editor.
- Change the name of the `<results_directory>` to `gcpResultsViscous`.
- Change `<dynamicFrictionCoefficient>` to `false` everywhere it appears in the file.

- For the second and third tasks, change <viscousFrictionCoefficient> to true.
- Change the value of <initial_dynamic_friction_coefficient> to 0.
- Change the value of <initial_viscous_friction_coefficient> to 0.3.
- Run **Ground Contact Model Personalization** with this settings file using the instructions provided above

Optional Exploration: For whichever friction model you decide is better, explore changing the following settings to see if they improve the ability of your personalized foot-ground contact models to reproduce the experimental ground reaction force/moment and foot motion data:

- Run GCP without filtering the input inverse kinematics data (i.e., use the original inverse kinematics results file Trial12_Gait_IK_results.mot).
- Double the grid density by changing the value of <grid_width> to 10 and <grid_height> to 22.
- Reduce the value of <neighborStandardDeviation> to 0.2.
- Reduce the <max_allowable_error> for neighbor_spring_constant to 500.
- Reduce the <latching_velocity> to 0.05.

Deliverables

1. Wall clock time (sec) for your GCP runs with Coulomb friction and viscous friction:
   Coulomb friction wall clock time: ____________ min
   Viscous friction wall clock time: ____________ min
2. RMS errors for both feet in matching experimental ground reaction forces (N), ground reaction moments (Nm), toes and hindfoot rotations (deg), and hindfoot translations (m) when using Coulomb friction and viscous friction (complete the table below):

		**Coulomb Friction**		**Viscous Friction**	
**Quantity**	**Direction**	**Right Foot**	**Left Foot**	**Right Foot**	**Left Foot**
RMS Force Error (N)	Anterior				
	Vertical				
	Lateral				
RMS Moment Error (Nm)	X				
	Y				
	Z				
RMS Rotation Error (deg)	Toes				
	Y				
	X				
	Z				
RMS Translation Error (m)	X				
	Y				
	Z				

2. For each friction model and each foot, a plot comparing post-GCP ground reaction forces and moments with experimental ground reaction forces and moments.
3. For each friction model and one foot, a color plot showing the distribution of spring stiffness values over the bottom surface of the foot.
4. The GCP settings files for the two GCP runs whose results are shown in the table above.
5. Post-GCP NMSM Pipeline models Full_Body_Walking_Model-GCP_Coulomb.osimx

produced by GCP using Coulomb friction and `Full_Body_Walking_Model-GCP_Viscous.osimx` produced by GCP using viscous friction.

6. A brief paragraph explaining which type of friction model – Coulomb or viscous – you think is more physically realistic based on the results in your table above and the various plots produced by the function `plotGcpResultsFromSettingsFile`.

MODULE 3: TRACKING OPTIMIZATION

In this module, you will use the NMSM Pipeline **Tracking Optimization** (TO) tool with your personalized skeletal model to create a dynamically consistent walking simulation that reproduces experimental joint motion, joint moment, and ground reaction data as closely as possible for a single gait cycle. **Tracking Optimization** results always provide the starting point for the NMSM Pipeline **Treatment Optimization** process. In the subsequent module, you will perform a **Verification Optimization** (VO) starting from your TO results to confirm that your TO results are reliable, followed by a **Design Optimization** (DO) starting from your VO results to design either a high tibial osteotomy surgery or a modified walking motion that reduces the peak adduction moment in both knees of a subject with bilateral medial knee osteoarthritis.

In preparation for this module, compare the root-mean-square translation, rotation, ground reaction force, and ground reaction moment errors produced using Coulomb and viscous friction in the two GCP runs you performed for the previous module. Select the NMSM Pipeline foot-ground contact model with the most physically realistic results and ideally the lowest errors. For "physically realistic," look for ground reaction force and moment results that make sense physically (e.g., the ground reaction forces and moments should be zero during swing phase when the foot is off the ground and zero at the transitions into and out of contact). Copy this NMSM Pipeline model and give it the new name `Full_Body_Walking_Model-Post_GCP.osimx`.

The TO run for this module will be performed using the following model and data files:
- OpenSim model file: `Full_Body_Walking_Model-Post_JMP.osim`
- NMSM Pipeline model file: `Full_Body_Walking_Model-Post_GCP.osimx`
- Inverse kinematics data file: `Trial12_Gait_IK_results_filtered.mot` (to be cropped – this data file was an input to, not an output from, **Ground Contact Model Personalization**)
- Ground reaction data file: `updated_Trial12_Gait_forces_filtered.mot` (to be cropped)
- Inverse dynamics data file: `Trial12_Gait_ID_results.sto` (to be created and cropped)

Once all necessary input data files are available, they will be cropped to a single gait cycle between 2.235 to 3.280 seconds, which is the same gait cycle used for the **Ground Contact Model Personalization** process. The cropping process will be performed using Matlab program `cropGaitData.m` provided with the project.

Note that this step uses an updated rather than the original ground reaction data file. This updated file was produced by **Ground Contact Model Personalization** and contains a small shift in the electrical center location of each force plate. The electrical center is the point about which moments are summed for a force plate. The location of this point often contains small errors (especially for split-belt instrumented treadmills) that make the ground reaction data inconsistent with the video motion capture data. **Ground Contact Model Personalization** corrected for this inconsistency during the third GCP task that matched all three components of ground reaction moments as well as all three components of ground reaction forces.

Module Task 1: Inverse Dynamics

The interaction between tool settings, data, and models required to perform this module task is shown in the figure below:

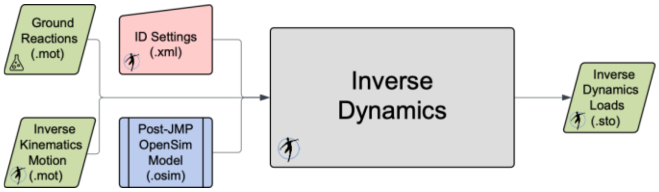

Step 1: Perform inverse dynamics using the gait trial experimental data.

- Copy the ground reactions data file `updated_Trial12_Gait_forces_filtered.mot` from the `GCP\gcpResults\GRFData` folder for your selected friction model to your `Inverse Dynamics` folder and rename it `Trial12_Gait_forces_filtered_updated.mot`.
- Load model `Full_Body_Walking_Model-Post_JMP.osim` into the OpenSim GUI and select the **Inverse Dynamics** tool.
- On the **Main Settings** tab, make the following selections:
  - Under **Input**, for the **From …** option, select your filtered inverse kinematics motion data file `Trial12_Gait_IK_results_filtered.mot`, check the **Filter coordinates** checkbox, and enter 6 Hz.
  - Under **Time**, keep the default **Time range to process** as 0 to 4 seconds, which was populated when you selected your inverse kinematics motion file.
  - Under **Output**, make the **Directory** your `Inverse Dynamics` data folder.
- On the **External Loads** tab, select the **External Loads** checkbox and click the pencil icon to bring up the **External Forces** set up window. In this window, select the ground reaction data to use as follows:
  - Click the folder icon next to **Force data file** and select your ground reactions data file `Trial12_Gait_forces_filtered_updated.mot`.
  - Click **Add** to add ground reaction data to the left and right feet. Use force plate 2 data for the right foot and force plate 1 data for the left foot. Make sure the force and torque are applied to the calcaneus body and that the force and point data are expressed in the ground coordinate system.
  - Save your ground reactions **External Forces** settings file (a sub-settings file within inverse dynamics) as `External_Forces_Settings_Gait.xml` using he **Save…** button at the bottom of the **External Forces** window.
- Once your **Inverse Dynamics** settings file is completed, save it to your hard disk using the **Save…** command at the bottom of the tool window and name it `ID_Settings_Gait.xml`.
- Run your **Inverse Dynamics** analysis and verify that the default inverse dynamics results file `invese_dynamics.sto` was saved to your `Inverse Dynamics` data folder. Rename this file `Trial12_Gait_ID_results.sto`.
- The inverse dynamics results generated in this step cover more than the gait cycle of interest, since the spline fitting process used to calculate joint velocities and accelerations has end effects that distort the values of the derivatives near the start time and end time. Performing inverse dynamics over a larger time window than needed and then cropping the results to the desired time window afterward eliminates these end effects.

Step 2: Crop all of your input data files to one gait cycle

- Open Matlab and change directories to your `Inverse Dynamics` data folder.
- Run the provided Matlab program `cropGaitData.m` with no inputs. The program will automatically pick the correct gait trial data and crop it using a start time of 2.235 second and

an end time of 3.280 seconds.

- After the program finishes, verify that new data files with the following names are present in your Inverse Dynamics data folder:
  - Trial12_Gait_IK_results_filtered_cropped.sto
  - Trial12_Gait_ID_results_cropped.sto
  - Trial12_Gait_forces_filtered_updated_cropped.sto

  Note that all three data files now have a .sto extension. OpenSim is agnostic between .mot and .sto file extensions but prefers the .sto extension. Consequently, from here forward, .sto files will be used throughout the **Treatment Optimization** process.

Module Task 2: Tracking Optimization

For this module task, you will used the NMSM Pipeline **Tracking Optimization** tool to produce a one-cycle dynamically consistent walking simulation based on your personalized post-JMP OpenSim model and your associated personalized post-GCP NMSM Pipeline foot-ground contact model. The optimization will seek to spread out errors in matching experimental joint motion from inverse kinematics, ground reaction forces and moments, and joint moments from inverse dynamics while driving the residual forces and torques acting on the pelvis segment to near zero. The personalized OpenSim/NMSM Pipeline skeletal model created by the **Model Personalization** process will provide the starting point for this module. The interaction between tool settings, data, and models required to perform this module task is shown in the figure below:

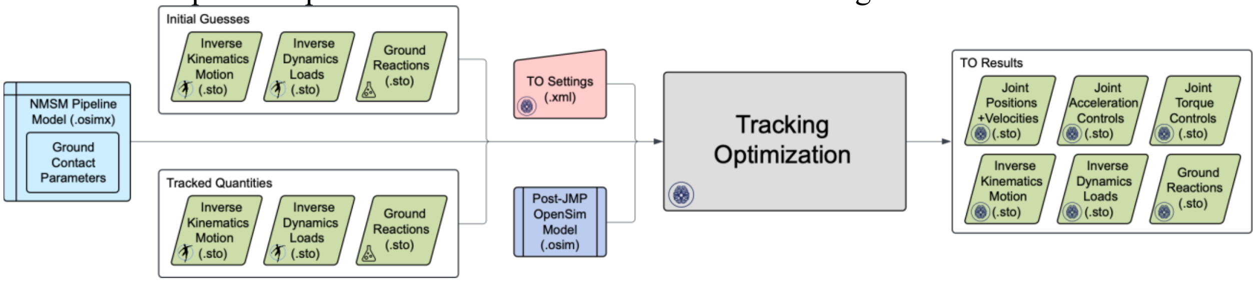

Step 1: Organize your input model and data files in preparation for your TO run

- Copy your OpenSim model file Full_Body_Walking_Model-Post_JMP.osim and your NMSM Pipeline model Full_Body_Walking_Model-Post_GCP.osimx file to your TO data folder.
- Create a folder called inputData within your TO data folder.
- Copy the three cropped gait data files created from your Inverse Dynamics folder to your inputData folder.
- Make the following changes within your inputData folder:
  - Move Trial12_Gait_IK_results_filtered_cropped.sto to the IKData folder and rename it Trial12_Gait.sto.
  - Move Trial12_Gait_ID_results_filtered_cropped.sto to the IDData folder and rename it Trial12_Gait.sto.
  - Move Trial12_Gait_force_filtered_updated_cropped.sto to the GRFData folder and rename it Trial12_Gait.sto.

  All three of these gait data files have the same file name by design. The folder in which each file is located tells the NMSM Pipeline what each data file is.

Step 2: Create your Tracking Optimization settings file

- Load the post-JMP model Full_Body_Walking_Model-Post_JMP.osim into the OpenSim GUI. NMSM Pipeline tools will not be accessible in the OpenSim GUI **Tools** menu unless a model to simulate is loaded first.
- Select **Tools** ⇨ **User Plugins** ⇨ **rcnlPlugin.dll** to load the NMSM Pipeline tools into the OpenSim GUI **Tools** menu.
- Select the **Tracking Optimization** tool to set up a TO run that reproduces the inverse kinematics, inverse dynamics, and ground reaction data for the one-cycle gait motion as closely as possible while making the simulated motion dynamically consistent.
- On the **Settings** tab, make the following choices in the **Input** section:
  - Leave the pre-selected model name and path unchanged.
  - For **Osimx File:**, choose Full_Body_Walking_Model-Post_GCP.osimx.
  - For **Initial Guess Dir:**, choose your inputData directory.
  - For **Tracked Quantities Dir:**, also chose your inputData directory since the same data will be used for both an initial guess for the solution and the tracked data for the cost function and constraints.
  - For **Trial Prefix:**, input Trial12_Gait.
- Still on the **Settings** tab, make the following choice in the **Output** section:
  - For **Results Dir:**, create a new folder in your TO directory called toResults.
- Still on the **Settings** tab, make the following choice in the **Optimal Control Solver Settings File** section:
  - Click the folder icon on the **Browse..** line and select the file gpopsSettings.xml in your TO directory. This file contains default GPOPS-II optimal control solver settings that tend to work well for any **Treatment Optimization** problem.
- Still on the **Settings** tab, for the **States Coordinates List**:, select all unlocked (i.e., changeable) generalized coordinates in your OpenSim model Full_Body_Walking_Model-Post_JMP.osim. DO NOT select any locked coordinates for your states.
- On the **RCNL Controllers** tab, scroll down to the **RCNL Torque Controller** section, click on **Edit…**, and select the following coordinates to be controlled by torque controllers: hip_flexion_r, hip_adduction_r, hip_rotation_r, knee_angle_r, ankle_angle_r, subtalar_angle_r, hip_flexion_l, hip_adduction_l, hip_rotation_l, knee_angle_l, ankle_angle_l, subtalar_angle_l.
  Torque controllers are placed on only those joints that would be controlled by muscles if muscles were present in the model.
- On the **Cost/Constraints** tab, in the **Cost Terms** section at the top, click on **Add…** and add the cost terms and associated maximum allowable errors as outlined in the table below:

<type>	Entities	<max_allowable_error>
generalized_coordinate_tracking	pelvis_tx pelvis_tz	0.02
generalized_coordinate_tracking	pelvis_tilt pelvis_list pelvis_rotation hip_flexion_r hip_adduction_r hip_rotation_r knee_angle_r ankle_angle_r hip_flexion_l	0.1745

	hip_adduction_l<br>hip_rotation_l<br>knee_angle_l<br>ankle_angle_l	
generalized_coordinate_tracking	subtalar_angle_r<br>mtp_angle_r<br>subtalar_angle_l<br>mtp_angle_l	0.0873
generalized_coordinate_tracking	lumbar_extension	0.6981
generalized_coordinate_tracking	lumbar_bending<br>lumbar_rotation<br>arm_flex_r arm_add_r<br>arm_rot_r elbow_flex_r<br>arm_flex_l arm_add_l<br>arm_rot_l elbow_flex_l	0.3491
generalized_speed_tracking	pelvis_tx pelvis_tz	0.2
generalized_speed_tracking	pelvis_tilt pelvis_list<br>pelvis_rotation<br>hip_flexion_r<br>hip_adduction_r<br>hip_rotation_r<br>knee_angle_r<br>ankle_angle_r<br>hip_flexion_l<br>hip_adduction_l<br>hip_rotation_l<br>knee_angle_l<br>ankle_angle_l	1.7453
generalized_speed_tracking	subtalar_angle_r<br>mtp_angle_r<br>subtalar_angle_l<br>mtp_angle_l	0.8727
generalized_speed_tracking	lumbar_extension<br>lumbar_bending<br>lumbar_rotation<br>arm_flex_r arm_add_r<br>arm_rot_r elbow_flex_r<br>arm_flex_l arm_add_l<br>arm_rot_l elbow_flex_l	3.4907
inverse_dynamics_load_tracking	hip_flexion_r_moment<br>hip_adduction_r_moment<br>hip_rotation_r_moment<br>knee_angle_r_moment<br>ankle_angle_r_moment<br>subtalar_angle_r_moment<br>hip_flexion_l_moment	10

	hip_adduction_l_moment<br>hip_rotation_l_moment<br>knee_angle_l_moment<br>ankle_angle_l_moment<br>subtalar_angle_l_moment	
external_force_tracking	ground_force_2_vx<br>ground_force_2_vy<br>ground_force_2_vz<br>ground_force_1_vx<br>ground_force_1_vy<br>ground_force_1_vz	40
external_moment_tracking	ground_moment_2_mx<br>ground_moment_2_my<br>ground_moment_2_mz<br>ground_moment_1_mx<br>ground_moment_1_my<br>ground_moment_1_mz	10

The pelvis_tx and pelvis_tz coordinates should be tracked lightly, since you need only a small amount of error for them to keep the pelvis over a unique location on the treadmill. You should never track the pelvis_ty coordinate since the pelvis height needs to adjust to allow the foot-ground contact models to equilibrate properly. All maximum allowable errors above were scaled up by a factor of two from their original values to speed up convergence. Maximum allowable errors for generalized coordinate tracking were selected so that lower body joint angles are tracked well to facilitate reproducing the correct inverse dynamics joint moments, ankle, subtalar, and toes angles are tracked very well to facilitate reproducing the correct ground reaction forces and moments, and upper body joint angles are tracked less well to balance out the dynamics. Maximum allowable errors for generalized speed tracking were generally chosen to be 10x greater than corresponding errors for generalized coordinate tracking. Inverse dynamic load tracking is applied to all joints for which an **RCNL Torque Controller** is present.

- Still on the **Cost/Constraints** tab, in the **Constraint Terms** section at the bottom, click on **Add…** and add the constraint terms outlined in the table below:

<type>	Entities	<max_error>
generalized_coordinate_periodicity	pelvis_tx pelvis_ty<br>pelvis_tz	0.01
generalized_coordinate_periodicity	pelvis_tilt pelvis_list<br>pelvis_rotation<br>hip_flexion_r<br>hip_adduction_r<br>hip_rotation_r<br>knee_angle_r<br>ankle_angle_r<br>subtalar_angle_r<br>mtp_angle_r<br>hip_flexion_l<br>hip_adduction_l	0.0175

	hip_rotation_l knee_angle_l ankle_angle_l subtalar_angle_l mtp_angle_l lumbar_extension lumbar_bending lumbar_rotation arm_flex_r arm_add_r arm_rot_r elbow_flex_r arm_flex_l arm_add_l arm_rot_l elbow_flex_l	
generalized_speed_periodicity	pelvis_tx pelvis_ty pelvis_tz	0.1
generalized_speed_periodicity	pelvis_tilt pelvis_list pelvis_rotation hip_flexion_r hip_adduction_r hip_rotation_r knee_angle_r ankle_angle_r subtalar_angle_r mtp_angle_r hip_flexion_l hip_adduction_l hip_rotation_l knee_angle_l ankle_angle_l subtalar_angle_l mtp_angle_l lumbar_extension lumbar_bending lumbar_rotation arm_flex_r arm_add_r arm_rot_r elbow_flex_r arm_flex_l arm_add_l arm_rot_l elbow_flex_l	0.0873
kinetic_consistency	hip_flexion_r_moment hip_adduction_r_moment hip_rotation_r_moment knee_angle_r_moment ankle_angle_r_moment subtalar_angle_r_moment hip_flexion_l_moment hip_adduction_l_moment hip_rotation_l_moment	0.1

	knee_angle_l_moment<br>ankle_angle_l_moment<br>subtalar_angle_l_moment	
root_segment_residual_load	pelvis_tx_force<br>pelvis_ty_force<br>pelvis_tz_force	1
root_segment_residual_load	pelvis_tilt_moment<br>pelvis_list_moment<br>pelvis_rotation_moment	0.1
external_force_periodicity	ground_force_2_vx<br>ground_force_2_vy<br>ground_force_2_vz<br>ground_force_1_vx<br>ground_force_1_vy<br>ground_force_1_vz	5
external_moment_periodicity	ground_moment_2_mx<br>ground_moment_2_my<br>ground_moment_2_mz<br>ground_moment_1_mx<br>ground_moment_1_my<br>ground_moment_1_mz	1

For this problem, <min_error> should always be chosen to be the negative of <max_error>, though it need not be. Adding periodicity to the constraints makes the simulated gait cycle near-periodic, which is a required condition for performing predictive simulations of a single walking cycle in subsequent **Design Optimization** runs. The closer the specified constraints are to perfectly periodic, the longer the TO run will take to converge. A kinetic consistency constraint is required for all joints with an **RCNL Torque Controller**.

- Once you have finished configuring your TO settings file in the OpenSim GUI, **Save** your settings file in your `TO` folder and name it `TO_Settings.xml`. Remember that if you want to read your TO settings file back into the OpenSim GUI to modify it (as opposed to simply modifying it in a text editor), you will need to use a different file name when you re-save the settings file due to a bug in the GUI implementation.

Step 3: Run your Tracking Optimization settings file and plot your results
Once you have created your TO settings file, run the file and generate results by following the instructions below:

- Open Matlab, load the NMSM Pipeline project if necessary, and change directories to where your TO settings file is located.
- Run the TO tool in Matlab using the settings file you just saved by inputting the following commands into Matlab:

```
>> tic
>> TrackingOptimizationTool('TO_Settings.xml')
>> toc
```

  Since none of the Treatment Optimization tools are parallelized through Matlab, you do not need to start a Matlab parallel pool to avoid having parallel processing startup impact your total wall clock time.

- Plot your TO results using Matlab function `plotTreatmentOptimizationResultsFromSettingsFile.m` as shown below:
  `>> plotTreatmentOptimizationResultsFromSettingsFile('TO_Settings.xml')`
  This function will output plots of experimental and simulated generalized coordinates, generalized speeds, inverse dynamics loads, and ground reaction forces and moments, along with plots of simulated torque controls. At the top of each subplot is a root-mean-square error showing the difference between the experimental and simulated quantity.

Optional Exploration: The relative weighting of the cost function and constraint terms significantly affects the speed of **Tracking Optimization** convergence. Explore changing your TO settings file in the ways shown below and note how it changes both the solution and the number of iterations required for convergence:

- Multiply the `<max_allowable_error>` by 2 for each of your cost function terms.
- Divide the `<max_allowable_error>` by 2 for each of your cost function terms.
- Multiply the `<max_error>` and `<min_error>` by 2 for each of your constraint terms.
- Divide the `<max_error>` and `<min_error>` by 2 for each of your constraint terms.

Deliverables

1. Wall clock time (min) and number of iterations for your TO run.
   Wall clock time: _____________ min
   Number of iterations: _____________
2. RMS errors for both feet in matching experimental generalized coordinates (m or deg), generalized speeds (m/s or rad/sec), inverse dynamics loads (Nm), ground reaction forces (N), and ground reaction moments (Nm) (complete the table below):

	Generalized Coordinate (m or deg)	Generalized Speed (m/s or rad/s)	Inverse Dynamics Moment (Nm)
pelvis_tx_force			
pelvis_ty_force			
pelvis_tz_force			
pelvis_tilt			
pelvis_list			
pelvis_rotation			
hip_flexion_r			
hip_adduction_r			
hip_rotation_r			
knee_angle_r			
ankle_angle_r			
subtalar_angle_r			
mtp_angle_r			
hip_flexion_l			
hip_adduction_l			
hip_rotation_l			
knee_angle_l			
ankle_angle_l			

subtalar_angle_l			
mtp_angle_l			
lumbar_extension			
lumbar_bending			
lumbar_rotation			
arm_flex_r			
arm_add_r			
arm_rot_r			
elbow_flex_r			
arm_flex_l			
arm_add_l			
arm_rot_l			
elbow_flex_l			

3. RMS errors for both feet in matching experimental ground reaction forces (N) and ground reaction moments (Nm) (complete the table below):

	Ground Reaction (N or Nm)
ground_force_2_vx	
ground_force_2_vy	
ground_force_2_vz	
ground_force_1_vx	
ground_force_1_vy	
ground_force_1_vz	
ground_moment_2_mx	
ground_moment_2_my	
ground_moment_2_mz	
ground_moment_1_mx	
ground_moment_1_my	
ground_moment_1_mz	

4. Absolute values of the peak adduction moment for both knees in units of Nm and and in units of percent bodyweight x height (subject bodyweight was 714 N and height was 1.70 m).

	**Units of Nm**	**Units of %BWxHt**
knee_adduction_r_moment		
knee_adduction_l_moment		

5. Plots of your generalized coordinate, generalized speed, inverse dynamics load, and ground reaction force and moments errors, as generated by Matlab plotting function `plotTreatmentOptimizationResultsFromSettingsFile`.
6. Your TO settings file for the TO run whose results are shown in the tables above.
7. A brief paragraph discussing what you believe to be the "largest" error in your TO results (realizing that different types of errors have different units), why you believe this error is the "largest one," and one idea that could potentially improve it.

MODULE 4: VERIFICATION AND DESIGN OPTIMIZATION

In this module, you will use the NMSM Pipeline **Design Optimization** (DO) tool to design one or two personalized clinical treatments that reduce the peak adduction moment in both knees of a subject with bilateral medial compartment knee osteoarthritis. One treatment optimization problem will involve the design of personalized high tibial osteotomy surgery while the other will involve the design of personalized gait modifications. *Undergraduate students are required to complete only one of the two treatment optimization problems and may choose between the two, while graduate students are required to complete both treatment optimization problems.*

For both treatment optimization problems, you will need to perform a **Verification Optimization** (VO) followed by a **Design Optimization**. Your **Verification Optimization** will start from your **Tracking Optimization** (TO) solution to confirm that the torque controls produced by your TO run generate the correct lower body walking motion and ground reactions without tracking those quantities in the cost function. Similarly, your subsequent **Design Optimization** will start from your **Verification Optimization** solution to predict how the modeled treatment will affect the subject's walking function and knee adduction moment peaks.

The VO and DO runs for this module will be performed using the following model and data files:

- OpenSim model file: `Full_Body_Walking_Model-Post_JMP.osim`
- NMSM Pipeline model file: `Full_Body_Walking_Model-Post_GCP.osimx`
- Inverse kinematics, ground reaction, and inverse dynamics data files: found in your TO results folder `toResults`.

The general **Verification Optimization** and **Design Optimization** processes to be performed for both treatment optimization problems are described in the two module tasks below. Following this general description, specific details are provided for how these general processes should be modified to account for the unique aspects of each treatment optimization problem.

Module Task 1: Verification Optimization

For this module task, you will used the NMSM Pipeline **Verification Optimization** tool to verify that the lower body joint torque controls found by your **Tracking Optimization** produce the same walking motion and ground reactions as did your **Tracking Optimization** but without tracking those quantities in the optimization cost function. This "sanity check" optimization will seek to minimize changes in your **Tracking Optimization** lower body torque controls and upper body joint motions so that a dynamically consistent near-periodic walking motion is produced. Results generated by your **Tracking Optimization** will provide the starting point for your **Verification Optimization**. The interaction between tool settings, data, and models required to perform this module task is shown in the figure below:

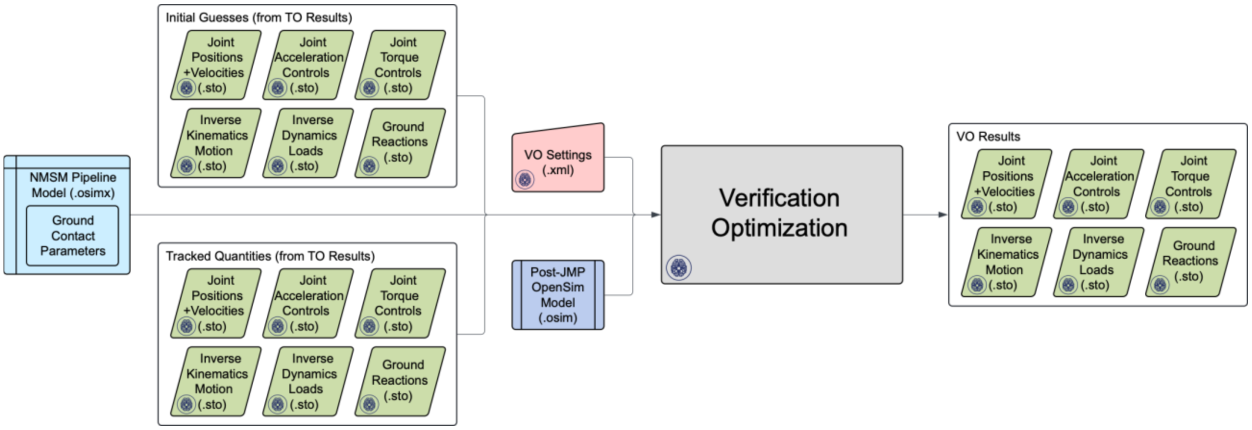

Your VO run will serve as a "dry run" for formulating the optimal control problem that you will solve in a subsequent DO run. You can think of your VO run as a DO run without the addition of the treatment design element. Normally a VO problem will be formulated primarily by removing cost and/or constraint terms from the original TO problem formulation. However, to make a VO run a "dry run" for a subsequent DO run, you will sometimes need to add one or more cost and/or constraint terms that were not present in your original TO problem formulation. When this situation arises, you will need to verify that the added cost and/or constraint terms do not affect the ability of your VO run to converge rapidly (i.e., within a few iterations) to your TO solution. If cost and/or constraint terms added to a VO problem formulation significantly increase the number of iterations required to converge, then the added cost and/or constraint terms are likely inconsistent with your base VO problem formulation containing only cost and constraint terms from your original TO run.

Step 1: Create your Verification Optimization settings file

- Copy your OpenSim model file `Full_Body_Walking_Model-Post_JMP.osim` and your NMSM Pipeline model file `Full_Body_Walking_Model-Post_GCP.osimx` to your `VO` data folder.
- Create a VO settings file that is a modified version of your previous TO settings file.
- If you do not want to follow the process outlined below to create your VO settings file using the OpenSim GUI, you can instead copy your TO settings file to a new settings file named `VO_Settings.xml`, open `VO_Settings.xml` in a text editor, change `TrackingOptimizationTool` to `VerificationOptimizationTool` at the top and bottom of the file, and make any additional changes to the VO settings file as described below.
- If you choose to use the OpenSim GUI to create your VO settings file, start by loading your post-JMP model `Full_Body_Walking_Model-Post_JMP.osim` into the OpenSim GUI. NMSM Pipeline tools will not be accessible in the OpenSim GUI **Tools** menu unless a model is loaded first.
- Select **Tools** ⇨ **User Plugins** ⇨ **rcnlPlugin.dll** to load the NMSM Pipeline tools into the OpenSim GUI **Tools** menu.
- Select the **Verification Optimization** tool to set up a VO run that tracks the lower body torque controls and upper body joint motions from your TO run while making the simulated motion dynamically consistent.
- On the **Settings** tab, make the following choices in the **Input** section:
  - Leave the pre-selected model name and path unchanged.

    - For **Osimx File:**, choose Full_Body_Walking_Model-Post_GCP.osimx.
    - For **Initial Guess Dir:**, choose your toResults directory.
    - For **Tracked Quantities Dir:**, also chose your toResults directory since the same data will be used for both an initial guess for the solution and the tracked data for the cost function and constraints.
    - For **Trial Prefix:**, input Trial12_Gait.
- Still on the **Settings** tab, make the following choice in the **Output** section:
    - For **Results Dir:**, create a new folder in your VO directory called voResults.
- Still on the **Settings** tab, make the following choice in the **Optimal Control Solver Settings File** section:
    - Click the folder icon on the **Browse..** line and select the file gpopsSettings.xml in your VO directory. This file contains default GPOPS-II optimal control solver settings that tend to work well for any **Treatment Optimization** problem.
- Still on the **Settings** tab, for the **States Coordinates List**:, select all moveable generalized coordinates in your OpenSim model Full_Body_Walking_Model-Post_JMP.osim.
- On the **RCNL Controllers** tab, scroll down to the **RCNL Torque Controller** section, click on **Edit…**, and select the following coordinates to be controlled by torque controllers: hip_flexion_r, hip_adduction_r, hip_rotation_r, knee_angle_r, ankle_angle_r, subtalar_angle_r, hip_flexion_l, hip_adduction_l, hip_rotation_l, knee_angle_l, ankle_angle_l, subtalar_angle_l.
  Torque controllers are placed on only those joints that would be controlled by muscles if muscles were present in the model.
- On the **Cost/Constraints** tab, in the **Cost Terms** section at the top, click on **Add…** and add the cost terms and associated maximum allowable errors as outlined in the table below:

<type>	Entities	<max_allowable_error>
controller_tracking (was inverse_dynamics_load_tracking in Tracking Optimization)	hip_flexion_r_moment<br>hip_adduction_r_moment<br>hip_rotation_r_moment<br>knee_angle_r_moment<br>ankle_angle_r_moment<br>subtalar_angle_r_moment<br>hip_flexion_l_moment<br>hip_adduction_l_moment<br>hip_rotation_l_moment<br>knee_angle_l_moment<br>ankle_angle_l_moment<br>subtalar_angle_l_moment	10
generalized_coordinate_tracking	mtp_angle_r<br>mtp_angle_l	0.0873
generalized_coordinate_tracking	lumbar_extension	0.6981
generalized_coordinate_tracking	lumbar_bending<br>lumbar_rotation<br>arm_flex_r arm_add_r<br>arm_rot_r elbow_flex_r<br>arm_flex_l arm_add_l<br>arm_rot_l elbow_flex_l	0.3491

These cost function terms come directly from your TO settings file except that every joint in the model is now controlled by either 1) controller tracking (lower body joints except for mtp joints), 2) generalized coordinate tracking (upper body joints plus mtp joints), or 3) no tracking (pelvis coordinates). In general, unlike for TO problem formulations, VO problem formulations generally have no joints controlled by both a cost function term and a constraint term (though this general rule can sometimes be violated to improve convergence for a subsequent DO run).

- Still on the **Cost/Constraints** tab, in the **Constraint Terms** section at the bottom, click on **Add…** and add the constraint terms outlined in the table below:

<type>	Entities	<max_error>
generalized_coordinate_periodicity	pelvis_tx pelvis_ty pelvis_tz	0.01
generalized_coordinate_periodicity	pelvis_tilt pelvis_list pelvis_rotation hip_flexion_r hip_adduction_r hip_rotation_r knee_angle_r ankle_angle_r subtalar_angle_r mtp_angle_r hip_flexion_l hip_adduction_l hip_rotation_l knee_angle_l ankle_angle_l subtalar_angle_l mtp_angle_l lumbar_extension lumbar_bending lumbar_rotation arm_flex_r arm_add_r arm_rot_r elbow_flex_r arm_flex_l arm_add_l arm_rot_l elbow_flex_l	0.0175
generalized_speed_periodicity	pelvis_tx pelvis_ty pelvis_tz	0.1
generalized_speed_periodicity	pelvis_tilt pelvis_list pelvis_rotation hip_flexion_r hip_adduction_r hip_rotation_r knee_angle_r ankle_angle_r subtalar_angle_r mtp_angle_r	0.0873

	hip_flexion_l hip_adduction_l hip_rotation_l knee_angle_l ankle_angle_l subtalar_angle_l mtp_angle_l lumbar_extension lumbar_bending lumbar_rotation arm_flex_r arm_add_r arm_rot_r elbow_flex_r arm_flex_l arm_add_l arm_rot_l elbow_flex_l	
kinetic_consistency	hip_flexion_r_moment hip_adduction_r_moment hip_rotation_r_moment knee_angle_r_moment ankle_angle_r_moment subtalar_angle_r_moment hip_flexion_l_moment hip_adduction_l_moment hip_rotation_l_moment knee_angle_l_moment ankle_angle_l_moment subtalar_angle_l_moment	0.1
root_segment_residual_load	pelvis_tx_force pelvis_ty_force pelvis_tz_force	1
root_segment_residual_load	pelvis_tilt_moment pelvis_list_moment pelvis_rotation_moment	0.1
external_force_periodicity	ground_force_2_vx ground_force_2_vy ground_force_2_vz ground_force_1_vx ground_force_1_vy ground_force_1_vz	5
external_moment_periodicity	ground_moment_2_mx ground_moment_2_my ground_moment_2_mz ground_moment_1_mx ground_moment_1_my ground_moment_1_mz	1

These constraint terms come directly from your TO settings file with no changes whatsoever.

- Once you have finished configuring your VO settings file in the OpenSim GUI, **Save** your

settings file in your `VO` folder and name it `VO_Settings.xml`. Remember that if you want to read your VO settings file back into the OpenSim GUI to modify it (as opposed to simply modifying it in a text editor), you will need to use a different file name when you re-save the settings file due to a bug in the GUI implementation.

Step 2: Run your Verification Optimization settings file and plot your results

Once you have created your VO settings file, run the file and generate results by following the instructions below:

- Open Matlab, load the NMSM Pipeline project if necessary, and change directories to where your VO settings file is located.
- Run the VO tool in Matlab using the settings file you just saved by inputting the following commands into Matlab:
  ```
  >> tic
  >> VerificationOptimizationTool('VO_Settings.xml')
  >> toc
  ```
  Since none of the Treatment Optimization tools are parallelized through Matlab, you do not need to start a Matlab parallel pool to avoid having parallel processing startup impact your total wall clock time.
- If your VO problem is formulated well, your VO run should converge in just a few iterations. If your VO run requires more than 10 iterations to converge, then something is wrong with your VO problem formulation, and you should review your VO settings file to try to diagnose the source of the problem. A common problem is to have a constraint term that is redundant with or conflicts with a cost function term. For example, you could have a periodicity constraint on a quantity that you are tracking in the cost function, but the tracked quantity is less periodic than required by the periodicity constraint.
- Plot your VO results using Matlab function `plotTreatmentOptimizationResultsFromSettingsFile.m` as shown below:
  ```
  >> plotTreatmentOptimizationResultsFromSettingsFile('VO_Settings.xml')
  ```
  This function will output plots of your TO and VO generalized coordinates, generalized speeds, inverse dynamics loads, ground reaction forces and moments, and simulated torque controls. At the top of each subplot is a root-mean-square (RMS) error showing the difference between TO and VO quantities.
- For all plotted quantities, verify that your VO results are visually identical to your TO results with extremely low RMS errors.

Module Task 2: Design Optimization

Following generation of a successful **Verification Optimization**, you will use the NMSM Pipeline **Design Optimization** tool to design a personalized treatment that reduces the subject's adduction moment peaks in both knees to a desired target value associated with an excellent long-term clinical outcome (Bryan *et al*., 1997). For either treatment optimization problem, results generated by your **Verification Optimization** will provide the starting point for your subsequent **Design Optimization**. The interaction between tool settings, data, and models required to perform this module task is shown in the figure below:

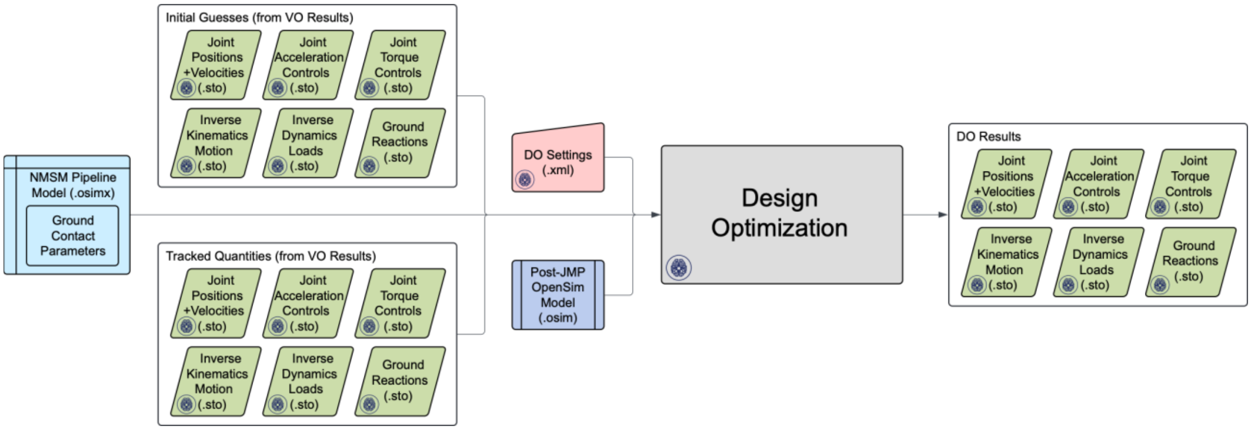

Step 1: Create your Design Optimization settings file

- Copy your OpenSim model file `Full_Body_Walking_Model-Post_JMP.osim` and your NMSM Pipeline model `Full_Body_Walking_Model-Post_GCP.osimx` file to your `DO` data folder.
- Create a DO settings file by modifying your VO settings file in a text editor (Note: You could also create this settings file using the OpenSim GUI, but creating it in a text editor is the fastest and easiest approach). Copy your VO settings file to a new settings file named `DO_Settings.xml`, open `DO_Settings.xml` in a text editor, and make the following changes:
  - Change `VerificationOptimizationTool` to `DesignOptimizationTool` at the top and bottom of the file.
  - Change the results directory to `doResults` in your `DO` folder.
  - Change the initial guess and tracked quantities directories to `voResults` within your `VO` folder.

Step 2: Run your Design Optimization settings file and plot your results

Once you have created your DO settings file, run the file and generate results by following the instructions below:

- Open Matlab, load the NMSM Pipeline project if necessary, and change directories to where your DO settings file is located.
- Run the DO tool in Matlab using the settings file you just saved by inputting the following commands into Matlab:
  ```
  >> tic
  >> DesignOptimizationTool('DO_Settings.xml')
  >> toc
  ```
  Since none of the Treatment Optimization tools are parallelized through Matlab, you do not need to start a Matlab parallel pool to avoid having parallel processing startup impact your total wall clock time.
- If your DO problem is formulated well, your DO run should converge in less than 250 iterations.
- Plot your DO results using Matlab function `plotTreatmentOptimizationResultsFromSettingsFile.m` as shown below:
  ```
  >> plotTreatmentOptimizationResultsFromSettingsFile('DO_Settings.xml')
  ```
  This function will output plots of your VO and DO generalized coordinates, generalized speeds, inverse dynamics loads, ground reaction forces and moments, and simulated torque

controls. At the top of each subplot is an RMS error showing the difference between VO and DO quantities.

- Record the predicted absolute value of the peak knee adduction moment for both knees. Recall that your treatment design goal is to achieve a peak knee adduction of 2.5 %BW*Ht in both knees. For the subject being modeled (BW = 714 N, Ht = 1.70 m), this target value is equivalent to 30.3 Nm.

Step 3: Animate your predicted motion in OpenSim

- Load your OpenSim model `Full_Body_Walking_Model-Post_JMP.osim` into the OpenSim GUI twice.
- For the first model, select **File** ⇨ **Load Motion…** and select the IK results file located in your `voResults\IKData` folder.
- For the second model, make the bones a different color, select **File** ⇨ **Load Motion…**, and select the IK results file located in your `doResults\IKData` folder.
- Sync the two motions and then animate them together at a slow **Speed** (e.g., 0.25). Note how much each knee in the predicted DO motion is medialized relative to the corresponding VO motion.

Given this general process for performing **Verification Optimization** and **Design Optimization**, problem-specific modifications required to solve each treatment optimization problem are described below:

Treatment Optimization Problem 1: Design of Personalized High Tibial Osteotomy Surgery

Step 1: Run a Verification Optimization

- Use the VO settings file described above with no modifications.
- Confirm that convergence to your TO solution occurs within just a few iterations.

Step 2: Add an HTO surgical correction to both tibias of your OpenSim model

- Copy your OpenSim model `Full_Body_Walking_Model-Post_JMP.osim` to a new model called `Full_Body_Walking_Model-Post_HTO.osim`.
- Open `Full_Body_Walking_Model-Post_HTO.osim` in a text editor and make the following changes:
  - At the top of the file, change the model name to `Full_Body_Walking_Model-Post_HTO`.
  - Search for coordinate name `knee_adduction_r` and change the default value to -0.0524 (3 deg), keeping the coordinate locked. This change sets the fixed knee adduction angle for the right tibia to 3 deg, emulating a 3 deg surgical correction in frontal plane leg alignment.
  - Repeat for coordinate name `knee_adduction_l`.
  - Save the model with these changes.

Step 3: Run a Design Optimization using your post-HTO OpenSim model

- Use the DO settings file described above except with the input model file changed to your post-HTO OpenSim model.
- Confirm that your DO run converges within roughly 250 or fewer iterations.

Step 4: Repeat using surgical corrections of 6 deg and 9 deg for both tibias

- Run a **Design Optimization** using surgical corrections of 3 deg for both tibias.
- Repeat using surgical corrections of 6 deg for both tibias.
- Repeat using surgical corrections of 9 deg for both tibias.
- Record the predicted peak adduction moment for each knee in the deliverables table below.

Step 5: Perform a quadratic fit to estimate the optimal surgical correction for each tibia

Step 6: Perform a final Design Optimization using the optimal surgical corrections

Deliverables

1. Wall clock time (min) and number of iterations for your final DO run.
   Wall clock time: ____________ min
   Number of iterations: ____________
2. Peak adduction moment for both knees using surgical corrections of 3, 6, and 9 deg for both tibias (complete the table below):

	Peak Adduction Moment (Nm)	
Surgical Correction (deg)	Right Knee	Left Knee
3		
6		
9		

3. Optimal surgical correction for each tibia based on a quadratic fit to the data in the table above:
   Optimal surgical correction for right tibia = ____________ deg
   Optimal surgical correction for left tibia = ____________ deg
4. Peak adduction moment for both knees predicted by your final DO run that used the post-HTO OpenSim model with optimal surgical correction for each tibia:
   Predicted peak adduction moment for right knee = ____________ Nm
   Predicted peak adduction moment for left knee = ____________ Nm
5. Plots of your inverse dynamics loads from your final DO run as generated by Matlab plotting function `plotTreatmentOptimizationResultsFromSettingsFile`.
6. The DO settings file for your final DO run that used the post-HTO OpenSim model with optimal surgical correction for each tibia.
7. A brief paragraph discussing how well the quadratic fit worked for estimating the optimal amount of surgical correction needed for each tibia to achieve post-HTO surgery knee adduction moment peaks of 30.3 Nm for each knee.

Treatment Optimization Problem 2: Design of Personalized Gait Modifications

Step 1: Run a modified Verification Optimization

- To match the optimization problem formulation used in (Fregly *et al.*, 2007), make the following additions to the VO settings file described above:
   - Add cost terms and associated maximum allowable errors as outlined in the table below:

<type>	Entities	<max_allowable_error>
`marker_position_tracking`<br>(x and z axes only)	`R_Heel`<br>`R_Toe`	0.0025

	L_Heel<br>L_Toe	
body_orientation_tracking<br>(x, y, and z axes using a yxz sequence)	calcn_r<br>calcn_l<br>torso	0.0175

These cost function terms make both feet follow the experimentally measured motion with respect to the lab coordinate system and make the torso follow the experimentally measured orientation with respect to the lab coordinate system. We do not track the foot marker y positions since we need to give the ground contact model freedom to equilibrate the model dynamics in the vertical direction.

- Add constraint terms and associated maximum/minimum errors as outlined in the table below:

<type>	Entities	<max_error>
marker_position_deviation<br>(x, y, and z axes)	R_Heel<br>R_Toe<br>L_Heel<br>L_Toe	0.0025
body_orientation_deviation<br>(x, y, and z axes using a yxz sequence)	calcn_r<br>calcn_l<br>torso	0.0175

These new constraint terms force the optimizer to find a solution where foot motion and torso orientation remain close to their experimental trajectories. Using a similar cost term and constraint term for marker positions and body orientations seems redundant but helps the optimizer converge more rapidly to a solution that matches the experimental foot motion and torso orientation closely.

- Confirm that convergence to your TO solution occurs within just a few iterations, despite the addition of these new cost function and constraint terms.

Step 2: Run a Design Optimization using your post-JMP OpenSim model

- Use the DO settings file described above (i.e., taken directly from your VO settings file) except with the addition of the following cost function term:

<type>	Entities	<max_allowable_error>
inverse_dynamics_load_minimization	knee_adduction_r_moment<br>knee_adduction_l_moment	10

- Confirm that your DO run converges within roughly 250 or fewer iterations.

Step 3: Repeat using max allowable errors of 15 and 20 Nm for both knee adduction moments

- Run a **Design Optimization** using maximum allowable errors of 10 Nm for both knee adduction moments.
- Repeat using maximum allowable errors of 15 Nm for both knee adduction moments.
- Repeat using maximum allowable errors of 20 Nm for both knee adduction moments.
- Record the predicted peak adduction moment for each knee in the deliverables table below.

Step 4: Perform a quadratic fit to estimate the optimal max allowable error for each knee

Step 5: Perform a final Design Optimization using the optimal max allowable error values

Deliverables

1. Wall clock time (min) and number of iterations for your final DO run.
   Wall clock time: ____________ min
   Number of iterations: ____________
2. Peak adduction moment for both knees using max allowable errors of 10, 15, and 20 Nm for both knee adduction moments (complete the table below):

Max Allowable Error (Nm)	Peak Adduction Moment (Nm) Right Knee	Left Knee
10		
15		
20		

3. Optimal max allowable error for each knee adduction moment based on a quadratic fit to the data in the table above:
   Optimal max allowable error for right knee adduction moment = ____________ Nm
   Optimal max allowable error for left knee adduction moment = ____________ Nm
4. Peak adduction moment for both knees predicted by your final DO run that used the optimal max allowable error for each knee adduction moment:
   Predicted peak adduction moment for right knee = ____________ Nm
   Predicted peak adduction moment for left knee = ____________ Nm
5. Plots of your inverse dynamics loads from your final DO run as generated by Matlab plotting function `plotTreatmentOptimizationResultsFromSettingsFile`.
6. The DO settings file for your final DO run that used the optimal max allowable error for each knee adduction moment.
7. A brief paragraph discussing how well the quadratic fit worked for estimating the optimal max allowable error for each knee adduction moment to achieve post-gait modification knee adduction moment peaks of 30.3 Nm for each knee.

References

Bryan, J.M., Hurwitz, D.E, Bach, B.R., Bittar, T., and Andriacchi, T.P. (1997) A predictive model of outcome in high tibial osteotomy surgery. *Proceedings of the 43rd Annual Meeting of the Orthopaedic Research Society*, p. 718.

Chèze, L., Fregly, B.J., and Dimnet, J. (1998) Determination of joint functional axes from noisy marker data using the finite helical axis. *Human Movement Science* **17**, 1-15

Fregly, B.J. (2021) A conceptual blueprint for making neuromusculoskeletal models clinically useful. *Applied Sciences, Special Issue on Musculoskeletal Models in a Clinical Perspective* **11**, Article 2037.

Fregly, B.J., Reinbolt, J.A., Rooney, K.L., Mitchell, K.H., and Chmielewski, T.L. (2007) Design of patient-specific gait modifications for knee osteoarthritis rehabilitation. *IEEE Transactions on Biomedical Engineering* **54**, 1687-1695.

Hammond, C.V., Williams, S.T., Vega, M.M., Ao, D., Li, G., Salati, R.M., Pariser, K.M., Shourijeh, M.S.* Habib, A.W., Patten, C., and Fregly, B.J. (2025) The Neuromusculoskeletal Modeling Pipeline: model personalization and treatment optimization functionality for OpenSim. *Journal of NeuroEngineering and Rehabilitation* **22**, Article 112.

Lai, A.K.M., Arnold, A.S., and Wakeling, J.M. (2017) Why are antagonist muscles co-activated in my simulation? A musculoskeletal model for analysing human locomotor tasks. *Annals of Biomedical Engineering* 45, 2762-2774.

Prodromos, C.C., Andriacchi, T.P., and Galante, J.O. (1985) A relationship between gait and clinical changes following high tibial osteotomy. *Journal of Bone and Joint Surgery American* **67**, 1188-1194.

Rajagopal, A., Dembia, C.L., DeMers, M.S., Delp, D.D., Hicks, J.L, and Delp, S.L. (2016) Full-body musculoskeletal model for muscle-driven simulation of human gait. *IEEE Transactions on Biomedical Engineering* **63**, 2068-2079.

Reinbolt, J.A., Schutte, J.F., Fregly, B.J., Haftka, R.T., George, A.D., and Mitchell, K.H. (2005) Determination of patient-specific multi-joint kinematic models through two-level optimization. *Journal of Biomechanics* **38**, 621-626.

Uhlrich, S.D., Jackson, R.W., Seth, A., Kolesar, J.A., and Delp, S.L. (2022) Muscle coordination retraining inspired by musculoskeletal simulations reduces knee contact force. *Scientific Reports* **12**, Article 9842.

van den Bogert, A.J., Smith, G.D., and Nigg, B.M. (1994) In vivo determination of the anatomical axes of the ankle joint complex: an optimization approach. *Journal of Biomechanics* **27**, 1477-1488.
