# Supplementary material for: Reproducible Research: Computational Design of Personalized Clinical Treatments for Walking Impairments Using the Neuromusculoskeletal Modeling Pipeline
Source: bioRxiv. 2026 Mar 4:2026.03.02.709099. Preprint. [Version 1] doi: 10.64898/2026.03.02.709099 (PMC12991138; doi:10.64898/2026.03.02.709099)
Supplement: Supplement 2 [file media-2.pdf]

SYNERGY-DRIVEN NEUROMUSCULOSKELETAL MODEL TREATMENT OPTIMIZATION
NMSM Pipeline Advanced Tutorial 2

Tutorial Developers: Robert Salati, Geng Li, and B.J. Fregly, Rice Computational Neuromechanics Lab, Rice University

Simulation Project Materials
The materials for this tutorial can be downloaded on SimTK at https://simtk.org/projects/nmsm under the "NMSM Advanced Tutorials" download.

Simulation Project Overview

The goal of this simulation project is to teach you the Neuromusculoskeletal Modeling (NMSM) Pipeline's computational treatment design process for clinical applications (Fregly 2021; Hammond et al. 2025) where muscles and neural control models are needed. This project will develop a treatment for a high-functioning stroke survivor using computationally design functional electrical stimulation (FES) patterns. Stroke survivors often have damaged neural pathways which impacts their ability to walk. As such, for this project, we will calibrate muscle-tendon parameters for the subject to accurately model their forces and then personalize a neural control model for the subject. Information from this neural control model will then be used to inform the treatment design process to design an optimal FES pattern (Cheung et al. 2019; Levine et al. 2025). This project will seek to design an FES pattern that compensates for a weakened synergy in the subject's affected limb and consequently improving their propulsive and braking ground reaction impulses to be more symmetric between limbs.

The five tools that you will use within the NMSM Pipeline are indicated in the table below, along with the abbreviations used to reference each tool and required supporting OpenSim tools:

NMSM Pipeline Toolset	NMSM Pipeline Tool	Used
Model Personalization	Joint Model Personalization (JMP)	Provided
	Muscle-tendon Model Personalization (MTP) (with OpenSim Muscle Analysis tool)	✓
	Neural Control Model Personalization (NCP)	✓
	Ground Contact Model Personalization (GCP)	Provided
Treatment Optimization	Tracking Optimization (TO)	✓
	Verification Optimization (VO)	✓
	Design Optimization (DO)	✓

For the **Model Personalization** toolset, you will only be using **Muscle-tendon Personalization (MTP)** and **Neural Control Personalization (NCP).** While a synergy-driven treatment optimization does require **Joint Model Personalization (JMP)** and **Ground Contact Personalization (GCP)**, these steps have already been done for you. All 3 Treatment Optimization

tools will be used in this project, but the model will now be controlled by synergy-driven muscle-tendon actuators.

This simulation project is broken down into four modules – Muscle-tendon Personalization, Neural Control Personalization, Tracking Optimization, and Verification/Design Optimization. For each module, instructions are provided to walk you through all the necessary steps. To ensure that poor results for one module do not affect your ability to complete subsequent modules, final results will be provided for each module to use as a starting point for subsequent modules. At the end of each module, you will be asked questions aimed at guiding you through interpreting your results.

To run each required OpenSim or NMSM Pipeline tool, you will generate an initial xml settings file using the appropriate tool selection within the OpenSim GUI Tools menu. Once you have generated an initial tool settings file in the OpenSim GUI, you can edit the settings file for subsequent tool runs either within the OpenSim GUI or using a text editor. Runs for OpenSim tools will be performed through the OpenSim GUI, while runs for NMSM Pipeline tools will be performed in Matlab.

This project will use treadmill gait data from a single high-functioning hemiparetic stroke survivor. For this individual, their right side is the paretic/affected side, and their left side is the less-affected side. All experimental joint angle, joint load, ground reaction, and electromyography (EMG) data have been provided to you inside the `InputData` folder but will need to be processed before being used for this project. The initial data provided to you is summarized below:

- `Trial10_IKResults.mot` – Joint angles from inverse kinematics (IK)
- `Trial10_forces_ec_reordered_filtered.mot` – Ground reaction forces (GRF) and moments with a shifted electrical center from GCP
- `Trial10_IDResults.sto` – Joint loads from inverse dynamics (ID)
- `Trial10_emg_processed.sto` – Processed EMG data

IK joint angles were solved using video-based marker motion capture (Vicon Corporation, Oxford, United Kingdom) with a post-JMP full-body OpenSim model. Six degree-of-freedom (DOF) ground reaction data were collected from a Bertec split-belt instrumented treadmill (Bertec Corporation, Columbus, OH, United States) with belts tied to the same speed. The experimental data from each trial above have already been converted so that all length data are in units of meters, all moment data are in units of Newton-meters, and all data are reported using coordinate axes consistent with OpenSim model conventions (i.e., +X is directed anteriorly, +Y is directed superiorly, and +Z is directed out to the right).

A scaled, post-JMP OpenSim model file is provided to you as well. The file name is `UF_Subject_4_Scaled_JMP.osim`.

MODULE 1: MUSCLE-TENDON PERSONALIZATION

In this module, you will do data processing and use the NMSM Pipeline Muscle-tendon Personalization (MTP) tool. MTP is classified as "EMG-driven" modeling. In other words, EMG data are an input to the model, rather than an unknown quantity. This is different from models based around Static Optimization, where muscle activity is not known. MTP's primary inputs are EMG data, kinematic data, and joint moment data. MTP will use EMG data in combination with muscle kinematics to generate individual muscle forces that apply moments about the model joints. These muscle joint moments need to match the experimental joint moment data. The main optimization in MTP calibrates Hill-type muscle model parameters so that the moments produced by muscles closely match experimental joint moments.

The Hill-type muscle model used in this project calculates muscle force according to the equation:

$$F = F_o^M \cdot \left[a\big(e(t-d)\big) \cdot f_l\left(\tilde{l}^M(t)\right) \cdot f_v\big(\tilde{v}^M(t)\big) + f_p\left(\tilde{l}^M(t)\right)\right] \cos(\alpha)$$

Where F_o^M is the maximum isometric force of the muscle, a is the muscle's activation which is a function of processed EMG data e, t is time, d is an electromechanical time delay, $\tilde{l}^M$ and $\tilde{v}^M$ are the normalized muscle fiber length and velocity respectively, and α is the muscle-tendon pennation angle. Tendons are assumed to be rigid, so $\tilde{l}^M$ and $\tilde{v}^M$ are calculated using the following equations:

$$\tilde{l}^M = \frac{l^{MT} - l_s^T}{l_o^M \cos(\alpha)}$$

$$\tilde{v}^M = \frac{v^{MT}}{10 \cdot l_o^M}$$

where l^{MT} is the muscle-tendon length, l_s^T is the tendon slack length, and l_o^M is the optimal muscle fiber length.

Of these variables, MTP calibrates l_o^M, l_s^T, and d. Additionally, MTP calibrates an activation time constant τ_{act} and activation non-linearity factor c_3. Finally, MTP calibrates muscle excitation scale factors to change the maximum amplitude of each EMG signal. For more details on these parameters, refer to (Meyer et al. 2017) and (Zajac 1989).

The MTP design variables are:

- **Max isometric force** – The maximum force a muscle can produce under isometric conditions.
- **Optimal muscle fiber length** – The fiber length at which a muscle can produce the maximum force. At the optimal fiber length, the normalized fiber length is 1.
- **Tendon slack length** – The "resting spring length" of the tendon attaching to the muscle. We are using rigid tendons, so the tendon slack length is equal to the length of the tendon.
- **Electromechanical delay** – A time delay between the measurement of the muscle excitation using an EMG sensor and when the excitation truly occurred.
- **Activation time constant** – A time constant governing the dynamics between a muscle excitation and a muscle activation.

- **Activation non-linearity constant** – A constant governing how non-linear the relationship between muscle excitation and muscle activation is.

The forces produced by a Hill-type muscle model have two components – active force and passive force. Active force is the force produced by contracting a muscle, and is a function of muscle activation a, normalized muscle fiber length $\tilde{l}^M(t)$, and normalized muscle fiber velocity $\tilde{v}^M(t)$. Passive force is the force produced by a muscle's intrinsic structure when the muscle is stretched too much. Passive force is only a function of normalized muscle fiber length $\tilde{l}^M(t)$, and is only present when a muscle's normalized fiber length exceeds 1. More intuitively, if you are doing stretches before going on a run, the tension you feel in the muscles is due to passive force – you are physically stretching the muscles, and the muscle structure resists that stretching.

MTP has 2 additional sub-tools that work along with the main body of the optimization. The first subtool is Muscle-tendon Length Initialization (MTLI). MTLI's purpose is to calibrate muscle-tendon length parameters and max isometric force values to make a better initial guess for MTP, and it always runs before MTP. To isolate muscle-tendon length parameters in the Hill-type force equation, MTLI only uses passive muscle forces. While passive force generated by individual muscles is difficult to measure, we can measure joint moments caused by these passive forces. This was done in a study that used a dynamometer to measure joint moments while moving joints through their range of motion with no muscle activation (Silder et al. 2007). MTLI calibrates muscle-tendon length parameters such that these passive joint moments are closely matched.

The second sub-tool is synergy extrapolation (SynX), which runs in parallel to MTP (Ao et al. 2022). SynX uses muscle synergies from the measured muscle excitations to estimate the excitations for muscles that weren't measured. This is important because even if a muscle isn't measured, we need to know how much force the muscle is output. If we chose to only include muscles that were measured, but could only measure 8 muscles per leg, the force from each of those muscles will be vastly overestimated. With SynX, we can simultaneously estimate the excitations for missing muscles and calibrate muscle parameters. The minimum number of EMG signals needed to get a good calibration with SynX is 8 EMG signals per leg (Ao and Fregly 2024)

The personalization will be performed using IK joint angles, ID joint loads, and processed EMG data. The interaction between tool settings, data, and models required to perform this module is shown in the figure below:

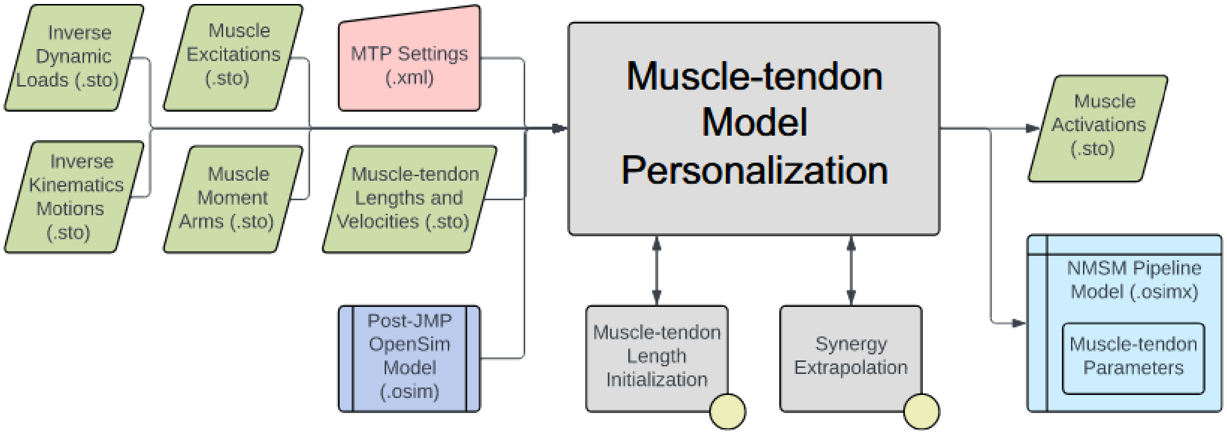

Data for IK joint motions, ID joint loads, and processed muscle excitations have already been provided. A post-JMP OpenSim model has also been provided. Muscle moment arms, and muscle-tendon lengths and velocities will be calculated in the first task for this module. In the next task, data will be processed to be in the correct format to use the MTP tool and the remainder of the NMSM Pipeline. Finally, you will conclude this module by running MTP.

Module Task 1: Muscle Analysis

A key step towards using MTP is to calculate muscular kinematic quantities such as moment arms, and muscle-tendon lengths. These quantities can be calculated using OpenSim's Muscle Analysis (MA) tool. The MA tool is a subtool of OpenSim's Analysis tool. A short guide to the Analysis tool can be found here.

The MA tool uses joint motions to calculate muscle-tendon lengths and muscle-moment arms about every joint using geometry calculations. Every modeled muscle has two attachment points on separate bodies such that they cross one or more joints and can apply moments about those joints. Anatomical muscles also have complex interactions with the bodies they're attached to. The OpenSim model used in this project represents these interactions using wrapping surfaces that define how muscles wrap around bones. These wrapping surfaces can be visualized in the OpenSim GUI by expanding the *bodies* tab under the model and then expanding individual bodies. The MA tool provides a quick and easy way to perform geometry calculations with these wrapping surfaces.

Step 1: Muscle analysis for gait

In this step, you will conduct MA on gait motion to calculate moment arms and muscle-tendon lengths throughout the gait cycle. These quantities will then be used in the main body of MTP to calculate muscle forces and corresponding joint moments. The input data files needed for this task step are in the `inputData` directory.

1. Load the model `UF_Subject_4_Scaled_JMP.osim` into the OpenSim GUI
2. Open the *Analyze tool*
3. Load the motion file `Trial10_IKResults.mot`

4. Change the prefix to **Trial10**
    a. This changes the prefix added at the beginning of every file created by MA. This is important for file organization
5. Change the output directory to `MuscleAnalysis\MAData`
6. Under the *Analyses tab*, add a MuscleAnalysis task.
7. Edit the MuscleAnalysis task and ensure that the tool will compute moment arms for all muscles and all lower body coordinates.
8. Run the MA tool.
9. Verify that the tool creates files inside `MuscleAnalysis\MAData` that all have the prefix "Trial10".
10. **Note:** There is a bug with MA in which the gastrocnemius muscles have moment arms about the hip. To work around this, copy and paste the MA files inside input data to your new MA folder. These files should replace some of the files you just calculated.

Step 2: Muscle analysis for passive moment data

The next MA run will be done on passive joint moment data. MTP uses passive moment data inside Muscle-tendon Length Initialization (MTLI) to generate a good initial guess for the main MTP optimization.

MTLI uses published passive joint moment data. As described earlier, joints are moved through their full range of motion with other joints fixed at constant values. Joint moments are then measured using a dynamometer as the joint moves through its range of motion. These data are described below:

- `Thelen_AnklePassive_01` – Ankle passive moments with the fixed at knee at 0°
- `Thelen_AnklePassive_02` – Ankle passive moments with the fixed at knee at 15°
- `Thelen_AnklePassive_03` – Ankle passive moments with the fixed at knee at 60°
- `Thelen_AnklePassive_04` – Ankle passive moments with the fixed at knee at 110°
- `Thelen_HipPassive_01` – Hip passive moments with the knee fixed at 15°
- `Thelen_HipPassive_02` – Hip passive moments with the knee fixed at 60°
- `Thelen_HipPassive_03` – Hip passive moments with the knee fixed at 90°
- `Thelen_HipPassive_04` – Hip passive moments with the knee fixed at 110°
- `Thelen_KneePassive_01` – Knee passive moments with the ankle fixed at 20°
- `Thelen_KneePassive_02` – Knee passive moments with the ankle fixed at -15°
- `Thelen_KneePassive_03` – Knee passive moments with the hip fixed at 45°
- `Thelen_KneePassive_04` – Knee passive moments with the ankle fixed at 20° and the hip fixed at -15°

Inside the `MuscleAnalysis` folder, there are two folders `thelen_r` and `thelen_l` that contain passive joint moment data for then right and left legs respectively. They contain folders `IKData` and `IDData`, where `IDData` contains the passive joint moments, and `IKData` contains the corresponding joint angles. You will run MA for all 12 motion trials in `IKData`, for both legs.

1. Open the Matlab file `passiveTrialMuscleAnalysis.m`
2. Edit the variable `thelenFolder` to say either "thelen_r" or "thelen_l"
3. Edit the variable `modelFileName` to be the full path to your `.osim` model file.
4. Run the Matlab script.

5. Repeat for the other leg.

These steps should create a folder called `MAData` inside each of *thelen_r* or *thelen_l*, and subfolders for each trial with muscle analysis files inside each folder. If these are not there, verify that all your file paths and names are correct.

Module Task 2: Data preprocessing

The next step in this module will be to process your data to get it into the correct format for the remainder of the NMSM Pipeline. This process will contain several parts, all of which are contained in the NMSM Pipeline's preprocessing tool:

1. **Process EMG data**: Starting from the raw EMG file, the script high pass filters, demeans, rectifies, and low pass filters the EMG signals. Next, any remaining negative EMG values are set to zero. EMG signals are then offset so that the minimum value of each signal is 0. Finally, EMG signals are normalized so that the max value of each signal is 1.
2. **Create muscle-tendon velocities**: The script low pass filters the muscle-tendon lengths created by Muscle Analysis, splines the filtered data using GCV splines, and then differentiates the filtered data.
3. **Crop and resample data**: A user input to preprocessing is a set of time pairs for which the data should be cropped to. One file is created for each time pair specified by the variable `trialTimePairs`. The data are then splined and resampled to have 101 time points per trial.
4. **Lowpass filter data**: All data are low pass filtered using the cutoff frequency specified by `inputSettings.cutoffFrequency`.

Data preprocessing is contained entirely in the script `Preprocessing\preprocessing.m`. The EMG data has already been processed, so those corresponding lines of code are commented out.

1. Open `Preprocessing\preprocessing.m`.
2. Edit `lines 12-17` with the paths to your data files.
3. We want to crop the data to one gait cycle (right heelstrike to right heelstrike). You must find the start time and the end time of the right heel strikes. To do this, open the file `Preprocessing\plotInputGroundReactions.m` and find the time of the first and second right heel strike (rounded <u>down</u> to the nearest 0.01s. ie if there is force starting at 0.98 seconds, round down to 0.97s).
4. Edit `startTime` and `endTime` to be the times you found above.
   a. This variable specifies the time ranges we want to crop the data into. This time pair isolates one single gait cycle that we will use for the rest of our modeling.
5. Explore the `preprocessed` directory that was created in your `Preprocessing` folder. This folder contains 5 folders for EMG data, IK data, ID data, GRF data, and MA data.
   a. <u>Keep note of where this folder is. You will be using it for the rest of the project.</u>
   b. <u>Do not change the name of any folders or files inside `preprocessed`. This tool outputs data in a format that the NMSM Pipeline expects from here on out. Changing any of these file or folder names will result in errors.</u>
6. Visualize your preprocessing results with the function `plotPreprocessing.m`
7. Verify that all data look good (ie minimal noise, no sharp discontinuities, other data quality issues)

Module Task 3: Muscle-tendon Personalization

Now that you have preprocessed data, you can move onto using the NMSM Pipeline's **Muscle-tendon Personalization (MTP)** tool. This tool will make use of the preprocessed data you just created, so there is no more work to be done for input data.

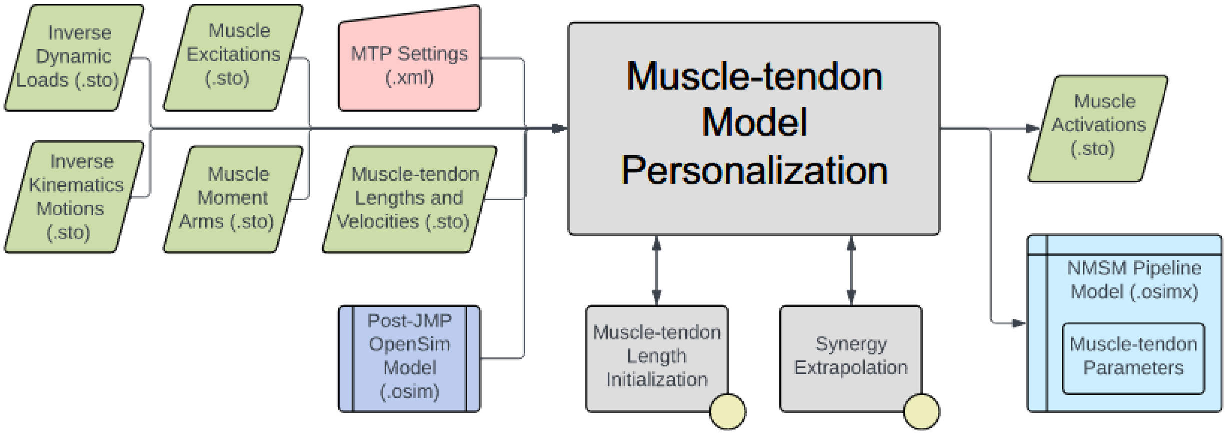

MTP will track this processed experimental data to fit Hill-type muscle model parameters for our model such that the joint moments generated by muscle forces match ID joint moments.

For this subproject, you will iterate through MTP solutions, changing cost term parameters until you arrive to a physiologically realistic solution.

Step 1) Explore muscle groups

1. Open the OpenSim model `UF_Subject_4_Scaled_JMP.osim` in the OpenSim GUI.
2. Under the *Forces* tab on the model, explore the muscles available.
3. Take note of the extra groups added.
   a. These are added for organization so that MTP/NCP knows which model muscles to group together in the optimization.
   b. The four important groups are:
      i. **Activation Muscle Groups** – Muscles that we would expect to have similar activation profiles (ie lateral hamstrings; BFSH and BFLH will have similar activations to each other). These groups all have ActivationGroup in their name.
      ii. **Normalized Fiber Length Muscle Groups** – Muscles that we would expect to have similar normalized fiber lengths. These groups all have NormalizedFiberLengthGroup in their name.
      iii. **Collected EMG Muscle Groups** – Muscle groups that we **do have** experimental EMG data for. Unlike the other groups, these must have the same name as the respective EMG channel name your EMG data file. They cannot be named [muscle]_collectedEmgMuscleGroup unless you change the corresponding names in your EMG data file `Preprocessing\preprocessed\EMGData\gait_1.sto`
      iv. **Missing EMG Muscle Groups** – Muscle groups that we **do not have** experimental EMG data for. These groups all have MissingEMGChannelGroup in their name.

The activation muscle groups and normalized fiber length muscle groups are organized based on how muscles are grouped anatomically. For example, we assume that your vastus muscles (three muscles that are a part of your quadriceps) will all have similar normalized fiber lengths and activation profiles because they serve similar functions. The collected and missing EMG muscle groups vary based on the dataset you are using, and which muscles have EMG data available.

The muscle groups for this project are premade for you but it is important to study them and understand why certain muscles are grouped together.

Step 2) Create your base settings files

You will need to create different settings files for the right and left leg. The reason for separating the legs is because SynX does not support multiple synergy sets. For this project, we are using 2 synergy sets – right leg and left leg. Therefore, MTP will need to be split up between the left and right leg. We will start with the right leg.

1. Load your premade post-JMP model `UF_Subject_4_Scaled_JMP.osim` into the OpenSim GUI
2. Open the Muscle-tendon Personalization Tool GUI
3. Set the *Input Osimx File* to be `GroundContactPersonalization\gcpResults\UF_Subject_4_Scaled_JMP_gcp.osimx`
4. Set the *Input Data Directory* to be `Preprocessing\preprocessed`
5. Set the *Output Results Directory* to be `MuscleTendonPersonalization\MTPResultsRightV1`
6. Set your *coordinate list* to be (**hip_flexion_r, hip_adduction_r, hip_rotation_r, knee_angle_r, ankle_angle_r, subtalar_angle_r**)
7. Set your *Activation Muscle Groups* to be all right leg activation groups
    a. Tip: You can use the *Filter by* box at the top of the selection window to search for only right leg activation groups
8. Set your *Normalized Fiber Length Muscle Groups* to be all right leg fiber length groups
9. Set your *Missing EMG Muscle Groups* to be all right leg missing EMG groups
10. Set your *Collected EMG Muscle Groups* to be all right leg EMG channels (Look at `Preprocessing\preprocessed\EMGData\gait_1.sto`)
11. Enable Muscle Tendon Length Initialization
12. Set the *Passive Data Input Directory* to be `MuscleAnalysis\thelen_r`
13. Enable Muscle Tendon Synergy Extrapolation with **6 synergies**
14. Save this settings file.
15. **Repeat the above steps for the left side of the body, changing all necessary fields.**
    a. You will need to change the Input Osimx File to be the file that is output from the right side MTP run. This file does not exist yet, so just copy and paste the following text into the field: `MTPResultsRightV1\ UF_Subject_4_Scaled_JMP_gcp_mtp.osimx`
16. Open `MuscleTendonPersonalization\runMTP.m`, and edit the settings files name and run the script.
    a. It's important that you run the right leg first because we use the output osimx file from the right leg as the input osimx file for the left leg.
    b. <u>MTP does not use any information from the input osimx file in the optimization.</u> The purpose of the input osimx file is so that MTP can concatenate left leg results to the right leg osimx file, keeping everything in the same file.

Now that your first MTP run is complete, the next step is to iterate through settings files to fine tune a solution. To fine tune an MTP solution, there are generally 4 parameters you should change:

1. **Minimum and maximum allowable normalized fiber lengths** – These terms dictate the range that we allow the normalized fiber length to fall in, and how heavily we punish normalized fiber lengths outside that range.

2. **Experimental quantity tracking terms** – These terms affect how closely the optimization tracks joint moments.
    a. **passive_joint_moment** in MTLI
        i. How closely passive joint moments should be tracked in MTLI
    b. **measured_inverse_dynamics_joint_moment** in SynX
        i. How closely all joint moments in the model should track experimental joint moments
        ii. Reduce max allowable error to prioritize SynX more.
    c. **inverse_dynamics_joint_moment** in MTP
        i. How closely joint moments produced by muscles with experimental EMG data should track experimental joint moments.
        ii. Reduce max allowable error to use experimentally measured muscles more.
3. **Max allowable errors and error centers for muscle group-based cost terms** – These terms punish deviations between muscles in a group.
    a. **grouped_normalized_muscle_fiber_length**
    b. **grouped_emg_scale_factor**
    c. **grouped_electromechanical_delay**
4. **Regularization terms** – These terms punish deviations in muscle-tendon parameters away from the error center and ensure the solution is unique.
    a. **optimal_muscle_fiber_length**
    b. **tendon_slack_length**
    c. **passive_muscle_force**
    d. **activation_time_constant**
    e. **activation_nonlinearity_constant**
    f. **emg_scale_factor**
    g. **muscle_excitation_penalty**

Some common problems in an MTP solution and corresponding solutions are:

1. **Poor joint moment tracking quality** (RMSE > 10Nm)
    a. Decrease moment tracking max allowable error
2. **Large muscle activations** (Max activation > 0.7 during gait)
    a. Decrease max allowable error/error center for **emg_scale_factor** or **muscle_excitation_penalty**.
3. **Large passive muscle forces** (Max passive force > 100)
    a. Decrease max allowable error on **maximum_normalized_muscle_fiber_length**, or **passive_muscle_force**
4. **Large difference between muscle activations and muscle excitations**
    h. **Decrease max allowable error on** activation_nonlinearity_constant**, or** activation_time_constant
5. **Large difference in activations or normalized fiber lengths between muscles in a group**
    a. Decrease max allowable error on **grouped_normalized_muscle_fiber_length**, **grouped_emg_scale_factor**, or **grouped_electromechanical_delay**
6. **Under/overuse of unmeasured muscles**

a. Change max allowable error on **`extrapolated_muscle_activation`** and **`residual_muscle_activation`**

Step 3) Iterate through MTP solutions

Iteration 1:

The first problem we will address is the large passive forces, especially in the calf and quad muscles. We will address this problem in a few ways. First, we will punish normalized fiber lengths falling outside of the optimal range of 0.8-1.0. Next, we will choose to optimize absolute length changes for optimal fiber length and tendon slack length (units of meters) instead of scale factors.

1. Create copies of your settings files and rename them.
2. For your new settings files, remember to update your `<results_directory>` fields, and your `<input_osimx_file>` field for the left settings file.
3. Passive forces are caused by normalized fiber lengths exceeding a value of 1, so we can first reduce the max allowable error on the maximum normalized fiber lengths inside MTLI to further punish normalized fiber lengths above 1.
    a. Inside `<MuscleTendonLengthInitialization>`, lower `<max_allowable_error>` of **`maximum_normalized_muscle_fiber_length`** to **`0.01`**
4. We will also increase the minimum normalized fiber length to avoid the normalized fiber length dropping too low.
    a. Inside `<MuscleTendonLengthInitialization>`:
        i. Increase `<min_normalized_muscle_fiber_length>` to **`0.8`**
        ii. Lower **`minimum_normalized_muscle_fiber_length`** `<max_allowable_error>` to **`0.01`**
5. To avoid over restricting the optimization, we can reduce the moment tracking value for MTLI as well.
    a. Inside `<MuscleTendonLengthInitialization>`, increase passive_joint_moment `<max_allowable_error>` to **`15`**
6. Next, we can change the optimization to optimize length parameters using absolute lengths instead of relative lengths. The previous optimization changed the scale factors of the optimal fiber length and tendon slack length. We will change this to optimize absolute changes in length, instead of scale factors
    a. Inside `<MuscleTendonLengthInitialization>`:
        i. Copy and paste the following: `<optimize_absolute_length_changes>`**`true`**`</optimize_absolute_length_changes>`
        ii. Change **`optimal_muscle_fiber_length`** max allowable error to **`0.005`** and the error center to **`0`**
        iii. Change **`tendon_slack_length`** max allowable error to **`0.005`** and the error center to **`0`**
7. Finally, inside the main optimization, we can reduce the max allowable error on the passive force cost term.
    a. Inside `<MTPTaskList>`, lower `<max_allowable_error>` of **`passive_muscle_force`** to **`10`**.

After running iteration 1, all passive forces should be below 50N. All normalized fiber lengths should be mostly between the range of 0.6-1.0. Some normalized fiber lengths may be a little bit above 1, but there should be a noticeable improvement over the initial MTP run.

Iteration 2:

The next observation is that certain muscles' activations are significantly different from their excitations, beyond a simple time shift (psoas & recfem for example). Also, some muscles have very large muscle activations which is not realistic during gait (Left leg psoas and iliacus). We will first address these problems by lowering the max allowable error on the activation non-linearity constant. This will punish high non-linearities that are causing muscle activations to have much higher amplitude than the corresponding muscle excitations. Next, we will tighten max allowable errors on muscle-excitations to punish the large muscle excitations.

1. Create copies of your settings files and rename them
2. For your new settings files, remember to update your <results_directory> fields, and your <input_osimx_file> field for the left settings file.
3. Change the following cost terms in both settings files:

   i. **`Muscle_excitation_penalty`**

      i. Max allowable error: **`0.3`**
      ii. Error center: **`0.3`**
      iii. This change will punish excitations from getting too high

   j. **`Emg_scale_factor`**

      i. Max allowable error: **`0.3`**
      ii. Error center: **`0.3`**
      iii. This change will also punish excitations from getting too high, but works with EMG scale factors rather than excitation magnitude.

   k. **`Activation_nonlinearity_constant`**

      i. Max allowable error: **`0.005`**
      ii. This change will move muscle activations closer to their excitations to fix muscles like the psoas.

Iteration 3:

This last iteration will address issues with SynX activations. Specifically, the right side SynX muscles (sartorius, piriformis, and quadratus femoris) are overactivated on the right leg and underactivated on the left leg. We will change this by tightening the max allowable errors on SynX. This iteration will also use different max allowable errors for the left and right sides (Why might this be?)

1. Create copies of your settings files and rename them
2. For your new settings files, remember to update your <results_directory> fields, and your <input_osimx_file> field for the left settings file.
3. Right side:
    a. Inside `<MTPSynergyExtrapolation>`:
        i. Change **`measured_inverse_dynamics_joint_moment`** max allowable error to **`2`**
        ii. Change **`extrapolated_muscle_activation`** max allowable error to **`0.1`**
    b. Inside `<MTPTask>`:
        i. Change **`inverse_dynamics_joint_moment`** max allowable error to **`3`**
4. Left side:
    a. Inside `<MTPSynergyExtrapolation>`:
        i. Change **`measured_inverse_dynamics_joint_moment`** max allowable error to **`1`**
        ii. Change **`extrapolated_muscle_activation`** max allowable error to **`1`**
    b. Inside `<MTPTask>`:
        i. Change **`inverse_dynamics_joint_moment`** max allowable error to **`3`**

Deliverables:

Inside one PDF file:

1. Submit your final plots of joint moments, normalized fiber lengths, muscle activations, and muscle passive forces for both legs.
2. In 1-2 sentences, explain how the MTP optimization works.
3. In 2-3 sentences each, explain how MTLI and SynX work, and why MTP needs them.
4. Summarize each MTP iteration you did. Information to include is:
    a. What was the goal of the iteration?
    b. What muscles were you aiming to fix?
    c. Did the iteration achieve its goal?
5. Briefly explain why the final settings files were different between the legs (Hint: what problem were we trying to solve in iteration 3? Was it the same problem for both legs?)
6. Compare the muscle activations between the left and right sides. Are there any muscles that have significantly different activations on the left and right sides?
7. Do you think that the final solution you got for MTP is good enough to move forwards? Why or why not?
8. If you were to do one more MTP iteration, what would your goal be? What muscles are you trying to fix, and what cost terms would you change to achieve this goal?
9. When selecting the joint moments to match during MTP, we didn't use the knee adduction moment or the mtp (toes) moment. Why didn't we use these moments?
    a. Toes: (Hint: Do we have joint moments for the toes? Why or why not? If not, how could we solve for toes joint moments?)
    b. Knee adduction: (Hint: What generates the knee adduction moment? Remember that the net moment about a joint is given by: $M^{net} = M^{contact} + M^{muscle} + M^{ligament}$)
10. In one paragraph explain why MTP is important for muscle-driven predictive simulations.

MODULE 2: NEURAL CONTROL PERSONALIZATION

In this module, you will use the NMSM Pipeline Neural Control Personalization (NCP) tool. NCP fits a set of muscle synergies to your data that best matches experimental muscle activations and reproduces experimental joint moments. Like MTP, NCP also calculates muscle joint moments and tries to track experimental joint moments. The design variables in NCP however, are the activations themselves. Specifically, NCP calculates a set of synergy activations and synergy vectors that create muscle activations that produce the correct muscle forces. It does this with a cost function that tracks muscle activations from MTP, and experimental joint moments. Because NCP is calculating muscle forces, it is best to use the muscle model calibrated in MTP. The desired output of NCP is a set of muscle synergies that are "functional", meaning when the muscle synergies are given to the model, they reproduce joint moments and can make the model walk.

It is important that synergies are functional for Treatment Optimization. In the next module, the forces generated by synergy-driven muscles will drive the motion of our gait model. We will then be able to change the muscle synergies to achieve our desired functional outcome for this stroke subject.

This calibration will be performed using ID joint moments, muscle moment arms, muscle-tendon lengths and velocities, and muscle activations (not excitations) generated by MTP. The interaction between tool settings, data, and models required to perform this module is shown in the figure below:

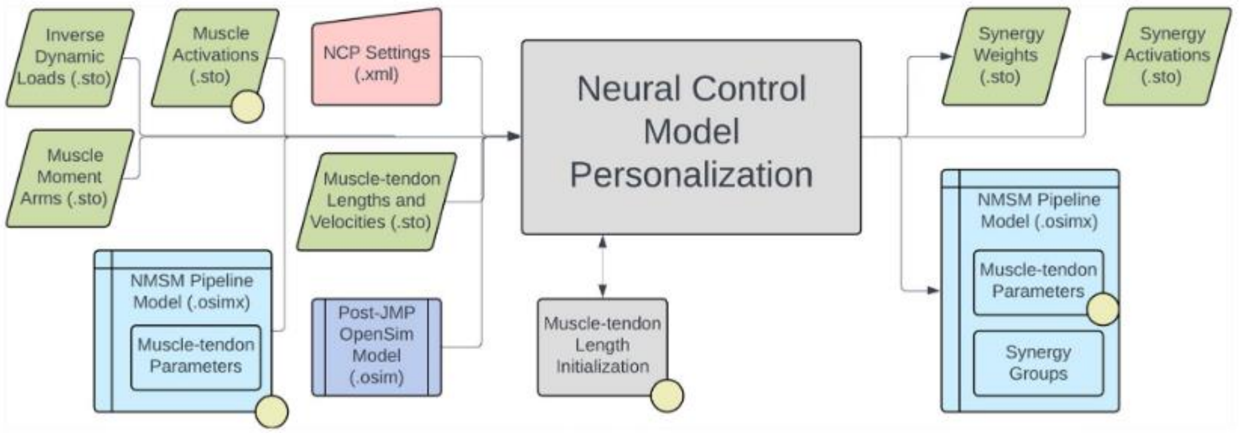

Because we ran MTP first, NCP will use the muscle model and muscle activations calibrated by MTP. The first task of this subproject will be to format your MTP results in the correct form for NCP. The second task will be to estimate how many synergies NCP should use. The third task will be to create an NCP settings file and fit your muscle synergies. The last task is to thoroughly analyze NCP results to diagnose this subject's impairment.

Module Task 1) Format NCP inputs directory

Unlike MTP, NCP does support multiple synergy sets, so we can combine our MTP results into a single NCP run. Therefore, the first task in this subproject is to combine your left and right MTP results.

There is a premade MATLAB script `NeuralControlPersonalization\CombineMTPResults.m` that combines results for you. Open this script and input the paths to your right and left MTP results directories.

The script will concatenate the output joint moments and muscle activations from both runs, and write the concatenated file to a new directory `NeuralControlPersonalization\mtpResultsCombined`. It will also copy the osimx file from your left leg MTP results directory to `mtpResultsCombined`.

Module Task 2) Estimate how many synergies to use in NCP.

At their core, muscle synergies are a mathematical decomposition of muscle activations. The more synergies you have, the better that decomposition will represent the original data. There are diminishing returns as more synergies are added, however. For a dataset with 16 EMG signals per leg, 5-6 synergies can typically represent more than 95% of the original data. Adding more synergies beyond that runs the risk of overfitting your experimental data which can make predicting new motions more difficult. To avoid overfitting our data, it is important to use a minimum set of synergies that represent the experimental data well.

The measure we use to quantify how well muscle synergies reproduce the original activations is called *percentage of variance accounted for* (%VAF), represented by the equation

$$\%VAF = \left(1 - \frac{\sum(x - x')^2}{\sum x}\right) \cdot 100$$

Where x and x' are the experimental EMG data and the reconstructed EMG data, respectively. For neuromusculoskeletal modeling, we aim for our synergies to have 90-95% VAF.

For this task, we will conduct a VAF analysis on our EMG data to estimate how many synergies we should use in our NCP run. Open the script `SynergyVafAnalysis.m` and fill out the required variables. This function will conduct a synergy analysis for k=4, 5, 6 synergies, plot the reconstructed muscle activations for visualization, and output %VAF values for each number of synergies.

Choose the minimum number of synergies that produces 95% VAF for the left and right side.
You will use this number of synergies for NCP.

Module Task 3) Run NCP

Now that you have an initial guess for how many synergies to use, we can create an NCP settings file.

Step 1) Initial NCP Settings file

1. Load your post-JMP model `UF_Subject_4_Scaled_JMP.osim` into the OpenSim GUI
2. Open the Neural Control Personalization Tool GUI
3. Set the *Input Osimx File* to be the Osimx file generated by your final left MTP run.
4. Set the *Input Data Directory* to `Preprocessing\preprocessed`
5. Set the *Output Results Directory* to be `NeuralControlPersonalization\NCPResultsNoBilateral`
6. Set your *coordinate list* to be all lower limb coordinates used in your previous MTP runs (left and right)
7. Set your *Activation Muscle Groups* to be every activation muscle group.
8. Set your *Normalized Fiber Length Groups* to be every normalized fiber length group.
9. Keep MTLI disabled.
10. Set your *MTP Results Directory* to be your combined MTP results directory.
11. Set your *Synergy Set* to be **right_leg** and **left_leg** with the number of synergies you decided on. Make sure both legs have the same number of synergies.
    a. Is it a good assumption for a stroke subject that both legs have the same number of synergies?
12. Save this settings file.
13. Open your settings file in a text editor.
14. Open your settings file in a text editor and edit the following cost terms inside of your `<RCNLCostTermSet>:`
    a. **moment_tracking** – max allowable error = 2
    b. **activation_tracking** – max allowable error = 0.015
15. Copy and paste the following into your `<RCNLCostTermSet>`:

```
<RCNLCostTerm>
   <type>grouped_activations</type>
   <is_enabled>true</is_enabled>
   <max_allowable_error>0.05</max_allowable_error>
</RCNLCostTerm>
```

16. Open the Matlab script `NeuralControlPersonalization\runNCP.`
17. This first NCP run may take ~30 minutes to run on the Duncan Hall computers.

Step 2) Experiment with bilateral symmetry

Your initial NCP run treated each leg as independent from the other. This might not be a realistic assumption, however. During a coordinated motion such as gait, your entire body is working as a system, so it is very likely that the synergies in your left and right leg are not acting independently from one another. This can be changed by including another cost term in NCP that adds coordination between the synergy sets in the body. This is called "bilateral symmetry".

When decomposing a set of muscle activations into muscle synergies, we are representing muscle activations by a set of time varying commands that control activation timing, and time-invariant weights that control coordination between muscles. There is literature suggesting that the time varying commands are mostly based inside the brain, while the time invariant synergy vector weights are based inside the spinal cord. When using bilateral symmetry, you are imposing that the synergy vectors between each leg must be the same. We are assuming that because the stroke only affects the brain, the synergy vector weights in the spinal cord are unaffected. The synergy activations can still be different from each other. In other words, the timing in activations between the legs can be different, but the way in which muscles are coordinated with each other should stay the same between legs.

1. Create a copy of your NCP settings file
2. Change your *Output Results Directory* to `NCPResultsBilateral5Synergies`.
3. Open your settings file in a text editor
4. Set `<enforce_bilateral_symmetry>` to **true**
5. This settings file will take longer to run than the previous settings file. As such, you are **not** required to run this settings file. Some premade results are inside `NCPResultsBilateral`.
6. Comment out the line to run NCP, plot the premade results, and answer the following questions.

Deliverables:

Inside one PDF file, submit all plots outputted by both NCP runs, and answer the following questions:

1. Fill out the following table with your %VAF values from task 2:

**Num Synergies**	**%VAF Right**	**%VAF Left**
**4**		
**5**		
**6**		

2. In 1-2 sentences, explain how the NCP optimization works.
3. Briefly explain what muscle synergies are, and why we might want to use them in neuromusculoskeletal simulations instead of individual muscle activations.
4. Why do we not want to use more synergies than required to reach 95% VAF?
5. Answer the following questions about bilateral symmetry:
   a. In 1-2 sentences, explain what bilateral symmetry is and why it's important.
   b. What %VAF did you get when running NCP with and without bilateral symmetry? Is this lower or higher than without bilateral symmetry? Does this result make sense?
   c. How did using bilateral symmetry affect your tracking quality of joint moments or muscle activations?
   d. Do you think that using bilateral symmetry is reasonable for a stroke subject?

The next group of questions will focus on analyzing the NCP results. You should use the NCP results with no bilateral symmetry that you created, and the premade results with bilateral symmetry.

First, we will analyze the solution without bilateral symmetry. To identify an impairment between the legs, it is important that we can compare synergies between both legs. This requires matching right leg and left leg synergies to each other. For the solution without bilateral symmetry, we can match synergies by identifying which synergies contain similar muscles on the left and right leg. Mathematically, this can be done by taking dot products between the left and right leg synergy vectors.

Inside `NeuralControlPersonalization`, open `CompareSynergyVectors.m`, fill out your NCP results directory for the run without bilateral symmetry, and click run. This script does two tasks:

1) Normalize synergy vectors – In order to ensure NCP's solution is unique, synergy vectors are normalized such that each vector has a maximum weight of 1. Because we want to take dot products to compare similarity between synergy vectors, we will renormalize all synergy vectors to have a magnitude of 1. In other words, we are turning the synergy vectors into unit vectors.
2) Make a grid of dot products between all combinations of left and right synergy vectors – We dot right_leg synergy vector 1 with left_leg synergy vectors 1, 2, 3, and so on, for all right leg synergies.

This script outputs a table with the dot products between all synergy vectors. A higher dot product corresponds to a more similar synergy activation.

6. Using the table created in `CompareSynergyVectors.m`, fill out the following table with the matching left leg synergy for each right leg synergy. Type N/A for boxes which you didn't use that many synergies.

Right Leg Synergy	Left Leg Synergy	Dot Product Value
**1**		
**2**		
**3**		
**4**		
**5**		
**6**		

7. How similar would you say your left and right leg synergies are? Do you think it is reasonable to compare these matched synergies to each other? Why or why not?

8. For the solution **with** bilateral symmetry, fill out the following table comparing the left and right synergy activations to each other. The magnitude of a synergy activation is its maximum value. The number of peaks is the number of prominent maximums in the activation. The location of the peaks should be reported as a percentage of the gait cycle, and a comma separated list if there are multiple peaks.

	**Right**			**Left**			
**Synergy Number**	**Magnitude**	**Number of peaks**	**Location of peaks**	**Magnitude**	**Number of peaks**	**Location of peaks**	**Similar shape? (yes/no)**
1							
2							
3							
4							
5							

A note on similar shapes: To decide if two synergies have a similar shape, you should be comparing the number of peaks and the locations of the peaks between the legs. Keep in mind that the left side will have a 50% gait cycle time shift from the right side. For example, if right leg synergy 1 has two peaks at 10% and 40% of the gait cycle, a "similar" left leg synergy 1 would have two peaks at 60% and 90% of the gait cycle. If both the right and left leg synergies have peaks at the same percentage in gait cycle, they are **not** similar to each other.

9. For the solution **with** bilateral symmetry, fill out the following table listing the primary muscles in each synergy. If a muscle has a synergy vector greater than 0.03 for a given synergy, write it in the table below.

Synergy Number	Primary Muscles in Synergy Vector
1	
2	
3	
4	

5	

10. Using the given table detailing muscle groups, fill out the below table with what muscle groups are most represented in each synergy.

**Synergy Number**	**Primary Muscle Groups in Synergy Vector**
1	
2	
3	
4	
5	

Primary muscle groups and their corresponding muscles are listed below:

**Muscle Group**	**Muscles in Group**
Hip Adductors	Addbrev, Addlong, Addmag
Hip Abductors	Glmax, Glmed, Glmin,
Hip Rotators	Piri, Tfl, Gem, Quadfem
Hip Flexors	Iliacus, Psoas, Recfem, Sart
Hip Extensors	Bflh, Glmax, Semimem, Semiten
Knee Flexors	Bflh, Bfsh, Semimem, Semiten, Gaslat, Gasmed
Knee Extensors	Recfem, Vasint, Vaslat, Vasmed
Ankle Plantarflexors	Gaslat, Gasmed, Soleus
Ankle Dorsiflexors	Tibant, Edl, Ehl
Ankle Inverters/Everters	Edl, Ehl, Perbrev, Perlong, Tibpost, Fdl, Fhl

11. Muscle synergies are often assigned a biomechanical function based on when in the gait cycle they are most active (Ting et al. 2015). Some common tasks assigned to synergies are:
    a. Weight acceptance
    b. Propulsion
    c. Flexion
    d. Leg swing

    The figure below shows the approximate shape of these types of synergies.

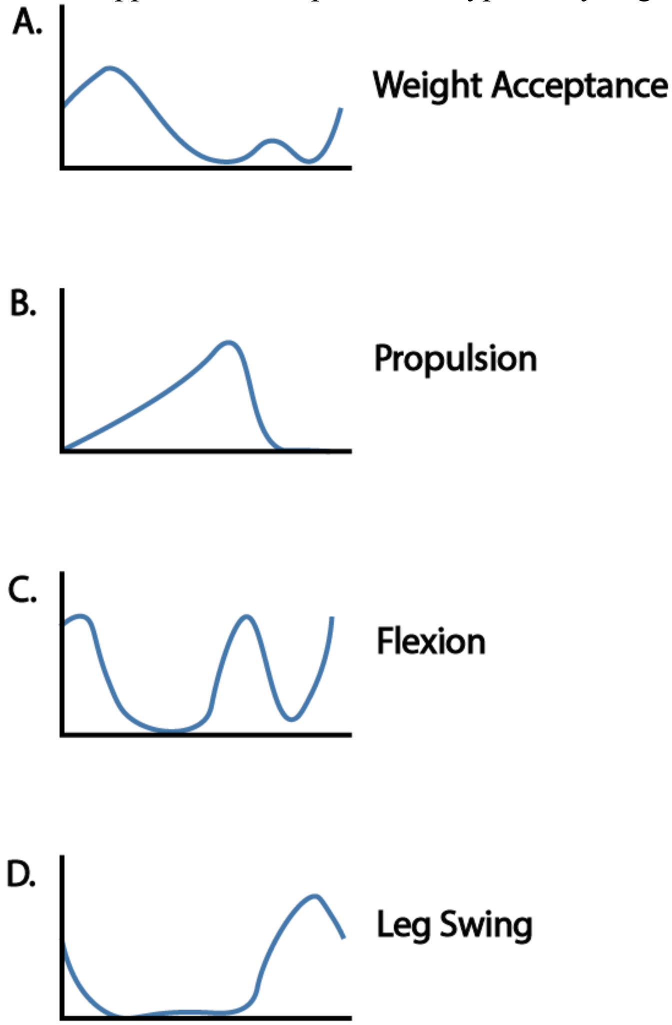

For the synergies with bilateral symmetry, fill out the following table with the function of each synergy. Reference the shapes of the synergy commands in the above figure. It is possible that multiple synergies serve the same function, or perhaps a mix of multiple functions.

**Synergy Number**	**Synergy Function**
1	
2	
3	
4	
5	

12. With this subject, we observe *impaired* synergies, and *compensatory* synergies. Impaired synergies are on the affected side (right side in this case) and have a significantly lower activation than the matching synergy on the non-affected side. Compensatory synergies can be on either side, and are characterized by a synergy activation that is moderately larger than the matching synergy on the other leg.
    a. Which right leg synergy (or synergies) are impaired? What function do these synergies serve? What muscle groups are in these synergies?
    b. Which right or left leg synergies are compensatory? What functions do these synergies serve?

MODULE 3: SYNERGY-DRIVEN TRACKING OPTIMIZATION

In this module, you will use the NMSM Pipeline Tracking Optimization tool with your personalized skeletal geometry and neural control model to create a dynamically consistent synergy driven walking simulation that closely reproduces experimental joint motion, joint moment, ground reaction, and EMG data. **Tracking Optimization** results always provide the starting point for the NMSM Pipeline **Treatment Optimization** Process. In the subsequent module, you will perform a **Verification Optimization** (VO) starting from your TO results to confirm that your TO results are reliable, followed by a **Design Optimization** (DO) to design a synergy-based FES treatment for our stroke subject.

Similar to module 2, synergy-driven TO problems can take a long time to run, and so this project will focus more on analysis of synergy-driven TO results rather than on running multiple iterations of TO. You will only do one synergy-driven TO run, where the focus is primarily on getting the optimization to converge, not on getting an optimal result. A results directory with final results will be provided as a part of the assignment for analysis.

Synergy-driven TO problems are formulated similarly to torque-driven TO problems, but are generally much harder for the optimal control solver to find a solution. This difficulty arises because actuating a model with muscles is more complicated than discrete torque actuators on muscle joints. The additional problem complexity means that Synergy-driven TO is very sensitive to the input data it is given. If the prerequisite model personalization was not high quality, synergy-driven TO will often struggle to converge on a good solution. However, if effort is put out to get good quality and anatomically correct model personalization results, synergy-driven TO can converge quickly.

Synergy-driven TO makes use of input data from a variety of sources throughout the NMSM Pipeline. Firstly, you should always use a post-JMP OpenSim model. If your problem includes ground reactions, TO expects an osimx file containing a GCP contact surface set. Because synergy-driven TO uses muscle actuators, it requires both MTP results and NCP results. TO uses the calibrated muscle models from NCP and uses the muscle synergies calculated by NCP as an initial guess to calculate more dynamically consistent synergies. Finally, synergy-driven TO expects that your input data is formatted as prescribed by the preprocessing sub-tool. Therefore, you will be using the same preprocessing directory that you have used for the previous subprojects. The interaction between tool settings, data, and models required to perform this module is shown in the figure below:

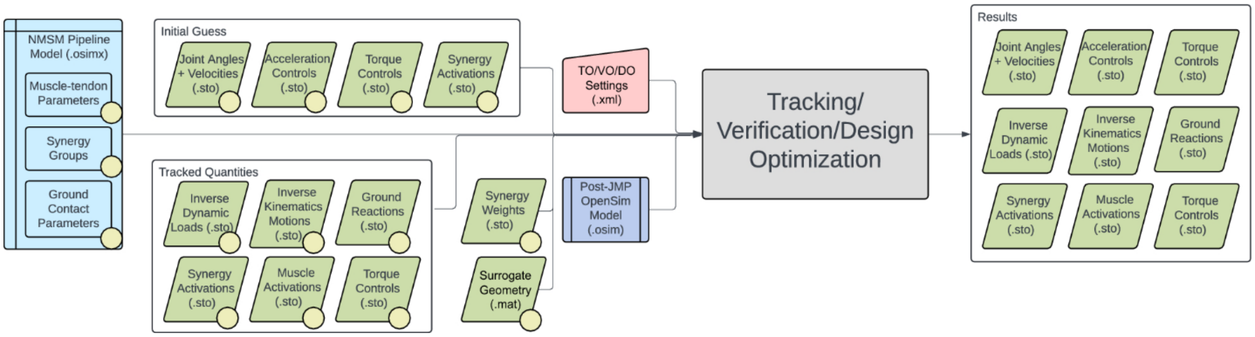

Module Task 1: Surrogate Muscle Geometry

An important step before all synergy-driven Tracking Optimization runs is to create a surrogate muscle geometry model. As we explored briefly in module 1, muscle geometry calculations are computationally expensive. Running the muscle analysis tool with all 86 muscles in the RCNL2025 model can take minutes for just one gait cycle. This was not problematic for MTP and NCP because the motion was not being changed by those tools, and so muscle analysis only needed to be run once. For Treatment Optimization however, the motion changes every iteration, and so muscle geometry needs to be recalculated repeatedly. This quickly becomes a problem for generating walking simulations.

The solution to this problem is to create a surrogate muscle geometry model that allows for muscle geometry calculations to be done much quicker. Rather than running a full muscle analysis calculation multiple times, we can fit a 6th order polynomial to the muscle geometry as a function of modeled joint angles. In short, we can run muscle analysis on a gait cycle and fit the muscle-tendon lengths and muscle-moment arms to the modeled joint angles. This polynomial model allows us to quickly and easily calculate muscle moment arms for a given model orientation.

The next problem that arises is selecting the proper motions to fit a surrogate model with. If we only use gait data to fit a surrogate model, then any new motions that deviate from the original motion will incur errors in muscle moment arms. On the other hand, we don't want to exhaustively sample every combination of model coordinates, because that will take far too much computation time to fit the surrogate model. The solution is to meet in the middle, and sample combinations of coordinates in the neighborhood around the original gait data. As such, the surrogate geometry uses a Latin hypercube sampling (LHS) algorithm to sample combinations of coordinates close to the original motion. LHS randomly searches combinations of coordinates using a minimal set of combinations that effectively covers the entire search space. We define the search space here to be the joint angles during gait motion ±20°.

Step 1) Create your surrogate kinematics

1. Open the Matlab script `surrogateKinematicsScript.m`
2. Fill out the variables for `modelFileName`, and `referenceKinematicsFile`.
    a. `referenceKinematicsFile` should be your IK data in `preprocessing`.
3. Set `angularPadding` value to **`20°`**
4. Set `linearPadding` to **`0.1m`**.
5. Click Run.
6. Start OpenSim and load `UF_Subject_4_Scaled_JMP.osim`
7. Load the motion `surrogateData\IKData\gait_1.sto`
    a. Analyze what is happening in this motion. You will be asked about it at the end of the assignment.

This surrogate kinematics script samples joint angles in the area around the given gait data. Ideally, these sampled joint angles will encompass any new predicted motions and allow their moment arms to be accurate and quickly calculated.

Step 2) Run muscle analysis

1. Open the Analyze tool in OpenSim.
2. Set your input motion to `surrogateData\IKData\gait_1.sto`
    a. Do **not** filter this motion.

   b. The motion looks very noisy but that is by design. The surrogate kinematics script adds "noise" to the base motion so that the sampling area for moment arms is larger.
3. Set your Prefix to **gait_1**
4. Set your output directory to `surrogateData\MAData\gait_1`
5. Add a muscle analysis set
6. Click run. This muscle analysis will take about 20 minutes.

You now have a set of muscle-tendon lengths and moment arms for every combination of joint angles produced in step 1. TO will automatically fit a polynomial to these values at the start of your run.

Module Task 2: Initial Tracking Optimization

This module task will focus on getting an initial guess synergy-driven Tracking Optimization. Because synergy-driven TO is such a complicated problem, one of the primary challenges in getting a good result is simply getting the optimal control problem to converge in the first place. A good method to get an initial TO to converge is to start the problem with very loose cost terms, and tight constraint terms. Once this loosely defined optimization converges, the next step is to progressively and systematically tighten cost terms and constraint terms until a satisfactory solution is obtained. This process allows us to diagnose if the constraint terms being used are compatible with each other, and systematically tightening the problem allows us to diagnose where the primary difficulties in convergence may come from.

Step 1) Create TO Settings File

1. Load your post-JMP model `UF_Subject_4_Scaled_JMP.osim` into the OpenSim GUI
2. Open the Tracking Optimization GUI
3. Set the *Input Osimx File* to be the Osimx file generated by your final NCP run
4. Set the *Tracked Quantities Directory* to `Preprocessing\preprocessed`
5. Set the *Initial Guess Directory* to your final NCP results directory
6. Set the *Trial Prefix* to gait_1
7. Set the *Results Directory* to `TrackingOptimization\SynergyTOResultsV1`
8. Set the *Optimal Control Settings File* to `TrackingOptimization\gpopsSettings.xml`
9. Add all unlocked coordinates to States Coordinates List.
10. Go to the *RCNL Controllers* tab
11. Keep *Optimize Synergy Vectors* disabled
12. For the *Coordinates List*, select all unlocked lower limb coordinates
13. Set the *Surrogate Model Data Directory* to `TrackingOptimization\surrogateData`
14. Go to the *Cost/Constraints* tab
15. Add cost and constraint terms according to the following tables:

Cost Terms

**Name**	**Type**	**Components**	**Max Allowable Error**
lower limb coordinate tracking loose	`generalized_coordinate_tracking`	`hip_flexion_r`<br>`knee_angle_r`<br>`ankle_angle_r`<br>`hip_flexion_l`<br>`knee_angle_l`<br>`ankle_angle_l`	0.3
Lower limb coordinate tracking tight	`generalized_coordinate_tracking`	`hip_adduction_r`<br>`hip_rotation_r`<br>`subtalar_angle_r`<br>`mtp_angle_r`<br>`hip_adduction_l`<br>`hip_rotation_l`<br>`subtalar_angle_l`<br>`mtp_angle_l`	0.15

Upper limb coordinate tracking loose	generalized_coordinate_tracking	lumbar_extension lumbar_bending lumbar_rotation arm_flex_r arm_add_r arm_rot_r elbow_flex_r arm_flex_l arm_add_l arm_rot_l elbow_flex_l	0.15
Pelvis translation tracking	generalized_coordinate_tracking	pelvis_tx pelvis_tz	0.05
Pelvis rotation tracking	generalized_coordinate_tracking	pelvis_tilt pelvis_list pelvis_rotation	0.15
ID load tracking	inverse_dynamics_load_tracking	hip_flexion_r_moment hip_adduction_r_moment hip_rotation_r_moment knee_angle_r_moment ankle_angle_r_moment subtalar_angle_r_moment hip_flexion_l_moment hip_adduction_l_moment hip_rotation_l_moment knee_angle_l_moment ankle_angle_l_moment subtalar_angle_l_moment	25
Speed tracking	generalized_speed_tracking	All unlocked coordinates	5
Vertical external force tracking	external_force_tracking	ground_force_1_vy ground_force_2_vy	100
Horizontal external force tracking	external_force_tracking	ground_force_1_vx ground_force_1_vz ground_force_2_vx ground_force_2_vz	25
External moment tracking y z	external_moment_tracking	ground_moment_1_my ground_moment_1_mz ground_moment_2_my ground_moment_2_mz	20
External moment tracking x	external_moment_tracking	ground_moment_1_mx ground_moment_2_mx	20
Muscle activation tracking	muscle_activation_tracking	*All model muscles*	0.25
Synergy activation tracking	controller_tracking	right_leg_1 right_leg_2 right_leg_3 right_leg_4 right_leg_5 left_leg_1 left_leg_2 left_leg_3 left_leg_4 left_leg_5	0.5

Constraint Terms

Name	Type	Components	Max/Min Error
Kinetic consistency	kinetic_consistency	hip_flexion_r_moment hip_adduction_r_moment hip_rotation_r_moment knee_angle_r_moment ankle_angle_r_moment subtalar_angle_r_moment hip_flexion_l_moment hip_adduction_l_moment hip_rotation_l_moment knee_angle_l_moment ankle_angle_l_moment subtalar_angle_l_moment	0.1/-0.1
Residual force reduction	root_segment_residual_load	pelvis_tx_force pelvis_ty_force pelvis_tz_force	25/-25
Residual moment reduction	root_segment_residual_load	pelvis_tilt_moment pelvis_list_moment pelvis_rotation_moment	10/-10
Rotational coordinate periodicity	generalized_coordinate_periodicity	pelvis_tilt pelvis_list pelvis_rotation hip_flexion_r hip_adduction_r hip_rotation_r knee_angle_r ankle_angle_r subtalar_angle_r mtp_angle_r hip_flexion_l hip_adduction_l hip_rotation_l knee_angle_l ankle_angle_l subtalar_angle_l mtp_angle_l lumbar_extension lumbar_bending lumbar_rotation arm_flex_r arm_add_r arm_rot_r elbow_flex_r arm_flex_l arm_add_l arm_rot_l	0.01/-0.01

		elbow_flex_l	
Translational coordinate periodicity	generalized_coordinate_periodicity	pelvis_tx<br>pelvis_ty<br>pelvis_tz	0.01/-0.01
External force periodicity	external_force_periodicity	ground_force_2_vx<br>ground_force_2_vy<br>ground_force_2_vz<br>ground_force_1_vx<br>ground_force_1_vy<br>ground_force_1_vz	5/-5
External moment periodicity	external_moment_periodicity	ground_moment_2_mx<br>ground_moment_2_my<br>ground_moment_2_mz<br>ground_moment_1_mx<br>ground_moment_1_my<br>ground_moment_1_mz	1/-1
Rotational speed periodicity	generalized_speed_periodicity	pelvis_tilt pelvis_list<br>pelvis_rotation<br>hip_flexion_r<br>hip_adduction_r<br>hip_rotation_r<br>knee_angle_r<br>ankle_angle_r<br>subtalar_angle_r<br>mtp_angle_r<br>hip_flexion_l<br>hip_adduction_l<br>hip_rotation_l<br>knee_angle_l<br>ankle_angle_l<br>subtalar_angle_l<br>mtp_angle_l<br>lumbar_extension<br>lumbar_bending<br>lumbar_rotation<br>arm_flex_r<br>arm_add_r<br>arm_rot_r<br>elbow_flex_r<br>arm_flex_l<br>arm_add_l<br>arm_rot_l<br>elbow_flex_l	0.1/-0.1
Translational speed periodicity	generalized_speed_periodicity	pelvis_tx<br>pelvis_ty<br>pelvis_tz	0.01/-0.01

16. Inside gpopsSettings.xml, change `<setup_nlp_max_iterations>` to 250
17. Inside your settings file, inside `<RCNLSynergyController>`, copy and paste:

```
<load_surrogate_model>true</load_surrogate_model>
```

Fitting the surrogate model can take a long time for our model, so this line loads a preexisting surrogate model into the TO run to save time.

18. Open the Matlab script `TrackingOptimization\runTO.m` and click Run
19. This TO run will take approximately an hour to run on the Duncan Hall Computers

Step 1a) Analyze Convergence

This step should be done while your TO is running.

After clicking Run on Matlab, you will see a similar output to the following appear after about 10 minutes:

iter	objective	inf_pr	inf_du	lg(mu)	||d||	lg(rg)	alpha_du	alpha_pr	ls
0	9.1398349e-02	2.10e+03	6.14e-03	0.0	0.00e+00	-	0.00e+00	0.00e+00	0
1	9.1034505e-02	2.07e+03	4.22e+00	-2.3	1.35e+00	-	9.98e-03	2.11e-02f	1
2	9.1321351e-02	1.99e+03	2.52e+01	-2.3	1.11e+00	-	1.21e-02	3.54e-02f	1
3	9.3515725e-02	1.82e+03	1.19e+02	-2.3	1.94e+00	-	2.23e-02	6.91e-02h	1

The main important columns in this output are iter, objective, inf_pr, and inf_du. The objective column is the value for the cost function, where a lower value is a better adherence to the cost function. Inf_pr and inf_du are the primal and dual infeasibility, respectively, and correspond to constraint satisfaction. Specifically, inf_pr corresponds to how well constraints are satisfied, and inf_pr corresponds to how well the cost function and constraint function agree with each other. If inf_pr is low (constraints are satisfied), but inf_du is high, that roughly means that the cost function could be lower than it currently is. In this case, the constraints might be satisfied, but the solution is not "optimal" because the cost function is still too high. The optimization is configured to converge when both inf_pr<9.99e-5 and inf_du<9.99e-1.

As mentioned earlier in the assignment, a key challenge in synergy-driven TO is simply getting the optimization to converge. The optimization you are running should take around 200 iterations to converge. During this time, if inf_pr>1.00e4 or inf_du>1e6 for more than 10 iterations, it is unlikely that your optimization will converge. To save time, you should stop the optimization and troubleshoot your settings file. Another sign that your optimization will likely not converge is if the letter "r" appears next to your iteration counter. This sign means that the optimizer entered "restoration" mode. Put simply, the optimizer found that the current search space was not feasible (meaning the constraints cannot be satisfied). While entering restoration mode does not always mean the optimization will not converge, it is typically good practice to cancel the optimization if it occurs because it is likely that the problem as a whole is not feasible.

Some likely problems that can cause an optimization to not converge are:

1) Data quality issues: This will likely not be a problem for this project but is important if you are doing your own research.
2) Conflicting constraint terms: If you have constraint terms with conditions that conflict with each other, the problem as a whole will be infeasible and inf_pr and inf_du will quickly increase.

3) Cost terms conflict with constraint terms: This often happens if cost terms are too tight and don't give the optimizer freedom to satisfy constraints. Alternatively, if you include inappropriate cost terms, constraints will also be difficult to match. An example of this is tracking pelvis_ty in the cost function. This cost term will not work well with residual reduction, because tracking pelvis_ty forces the model into the ground which can increase the vertical residual force.
4) Other infeasible constraints: Often constraints are simply not able to be satisfied. For example, in the kicking tutorial, we increase the toe marker velocity by using a constraint term to force the X velocity of the toe marker to be 14.3-14.4 m/s. If however, you include the Y and Z velocities in that constraint terms, the problem will not converge because those velocities will not reasonably reach 14.3-14.4 m/s

Check your settings file for these issues, and if you still are not sure why your optimization is not converging, email the instructor for help.

Step 2) Analyze Full TO Results

The previous section walked you through how to create an initial TO settings file. That TO should have converged, thereby demonstrating problem feasibility, but the settings still needed to be iterated on to get the best solution. Next, you can analyze a final TO run from the end of that iteration process.

In this run, cost and constraint terms were systematically tightened to yield an optimal solution. Another key change was that synergy vectors were optimized as well. We found that allowing small changes in synergy vectors is important for getting a good TO solution. To go along with changing the synergy vectors, we also track the NCP synergy vectors to make sure they don't change too much.

In the TO run you did in step 1, synergy vectors were not optimized, meaning we effectively assumed that the synergy vectors created by NCP were perfect. While the synergy vectors created by NCP should be very close to the true synergy vectors, giving TO some freedom to change them often helps get a better solution. One drawback to allowing synergy vectors to change is that it slows down the optimization significantly.

To view the settings file used to get these results, you may open `SynergyTOResultsFull\TOSettings.xml`. You can plot these TO results compared to your initial TO results by uncommenting the code on `line 7` inside `runTO.m`.

Deliverables:

Inside one PDF file, submit all plots outputted by your TO run, and answer the following questions.

1. In 1-2 sentences, describe the goal of Tracking Optimization
2. Why is synergy-driven tracking optimization generally harder than torque-driven tracking optimization? Give 2 reasons.
3. What is the main reason we want to use a surrogate muscle geometry model?
4. Briefly describe the process for creating a surrogate muscle geometry model.
5. Describe the motion that is outputted from `surrogateKinematicsScript.m`

Regarding the TO run you did on your own (step 1):

1. Why do you think some coordinate tracking terms have different max allowable errors than others?
2. **Briefly** describe the tracking quality for the following quantities. Mention the overall tracking quality, which components had better or worse tracking than others, and if you think the max allowable errors should be increased for the relevant cost term:
    a. Joint Angles
    b. Joint Velocities
    c. Joint Loads
    d. External Loads
    e. Muscle Activations
    f. Synergy Activations
3. If you were to iterate on this TO solution, what cost/constraint terms would you prioritize changing.
    a. **Exploration opportunities**: Feel free to change the max allowable errors and re-run TO. How did the results change? Were there any unexpected changes?

Regarding the full TO results given with the project (step 2):

1. Which constraint terms were changed between your initial TO run and the final TO run? Why were the other constraint terms kept the same?
2. Why do you think it is important to change synergy vectors in TO?
3. Did the synergies change very much during the TO run? Do the trends you observed in the synergies from module 2 still hold true?
4. It is important for TO results to visualize the TO results motion compared to the experimental motion. Follow the following steps to visualize your motion in OpenSim:
    a. Open up 2 instances of `UF_Subject_4_Scaled_JMP.osim` and make them different colors
        i. Right click on the *Bodies* tab under the model, and click Display>Colors
    b. Ensure both models have a zero model offset
        i. Right click on the model, and click Display>Model Offset
    c. Right click on each model, and click *Load Motion*
    d. Load `Preprocessing\preprocessed\IKData\IKDataFixedTime.sto` to one model
        i. `IKDataFixedTime.sto` is the same data as gait_1.sto, but the time column is offset to start at t=0.

e. Load `TrackingOptimization\SynergyTOResultsFull\IKData\gait_1.sto` to the other model.

f. Sync both IK motions.

   i. Under the *Motions* tab for each model, highlight the motions (called "DataType=double"), right click them, and select *Sync Motions*

   ii. To select multiple motions at once on Windows, hold down ctrl and click on each motion.

g. Play the motions at 0.1x speed.

5. Qualitatively describe how well the TO simulated tracks the experimental motion. Are there any significant differences between the motions? Why do you think that might be?

MODULE 4: SYNERGY-DRIVEN VERIFICATION AND DESIGN OPTIMIZATION

In this module, you will use the Design Optimization (DO) tool with your previously generated Tracking Optimization (TO) walking simulation results to design a synergy-based FES treatment protocol for a stroke subject. You will first use Verification Optimization (VO) to sanity check your TO solution, and then you will implement cost terms in DO to modify the subject's gait in the desired way.

Because strokes involve neural impairments, your modeled treatments will focus on modifying the synergy controls produced by TO. This contrasts with the previous project with torque-driven Treatment Optimization, where the changes in DO were primarily in the subject's kinematics. This different treatment methodology means that the results might be a little bit less intuitive and take more effort to analyze.

Module Task 1) Verification Optimization

For this module task, you will use the NMSM Pipeline **Verification Optimization (VO)** tool to verify that the muscle synergy controls found by your Tracking Optimization (TO) produce the same walking motion and ground reactions as the TO did, but without tracking those quantities. VO will seek to minimize changes in your TO muscle synergy controls so that a dynamically consistent, periodic walking motion is produced. Results generated by your TO will provide the starting point for your VO.

VO serves as a "sanity check" for your TO results before moving further into the Treatment Optimization Process. It is possible that your TO run could have converged to a good-looking solution but had problems that will make a Design Optimization (DO) difficult. VO allows you to test that the input data that you give to DO is consistent with itself. Additionally, VO may serve as a "dry run" for your DO run. In other words, your VO run will be identical to your DO run, but without any of the design elements of the DO run. For example, in this project, we will design a new walking motion that changes synergy activations and ground reactions. As such, the VO run will look identical to the DO run, with all relevant constraints that will be used in DO, but without the cost terms that change the synergy activations and ground reactions.

A synergy-driven VO should converge in around 50 iterations, and the VO solution should look close to identical to the TO solution. In the case that your VO does not converge quickly, it is likely caused by one or more of the following:

1. You added constraint terms to your VO that are inconsistent with the solution. This often leads to the optimizer going into restoration mode.
2. The cost terms used in your VO somehow conflict with each other or other constraint terms
3. Your TO solution itself has a problem that needs to be fixed.

Step 1) Create your VO settings file

18. Create a copy of `TrackingOptimization\TOSettings.xml` into `VerificationOptimization`.
19. Rename the copied file VOSettingsTemplate.xml
20. Open VOSettings.xml in a text editor and change the XML headings at the top and bottom of the file from <TrackingOptimizationTool> to <VerificationOptimizationTool>
21. Disable <optimize_synergy_vectors>
22. Load your post-JMP model UF_Subject_4_Scaled_JMP.osim into the OpenSim GUI
23. Open the Verification Optimization GUI
24. Load your settings file VOSettings.xml into the Verification Optimization GUI.
25. Set the *Input Osimx File* to NeuralControlPersonalization\NCPResultsBilateral\UF_Subject_4_Scaled_JMP_gcp_mtp_mtp_ncp.osimx
    a. If the text box is red when you load the GUI in, you must re-select this file or the GUI will not be able to find it. The GUI does some light parsing through this file to locate ground reaction force and moment names.
26. Set the *Tracked Quantities and Initial Guess Directories* to TrackingOptimization\TOResults.
27. Set the *Results Directory* to VerificationOptimization\VOResults
28. Set the *Surrogate Model Data Directory* to TrackingOptimization\surrogateData
29. Click to **Cost/Constraints**
30. Delete all cost terms except for:
    a. Upper Limb Coordinate Tracking
    b. Pelvis Translation Tracking
    c. Pelvis Rotation Tracking
31. Add the following cost terms:

**Name**	**Type**	**Components**	**Max Allowable Error**
Synergy Controller Tracking	`controller_tracking`	`right_leg_1`<br>`right_leg_2`<br>`right_leg_3`<br>`right_leg_4`<br>`right_leg_5`<br>`left_leg_1`<br>`left_leg_2`<br>`left_leg_3`<br>`left_leg_4`<br>`left_leg_5`	1
Toes Coordinate Tracking	`generalized_coordinate_tracking`	`mtp_angle_r`<br>`mtp_angle_l`	0.025

32. Add the following constraint terms:

**Name**	**Type**	**Components**	**Max/Min Error**
Initial Coordinate Deviation	`initial_generalized_coordinate_deviation`	`All coordinates in the States Coordinate List`	0.01/-0.01

Initial Speed Deviation	initial_generalized_speed_deviation	All coordinates in the States Coordinate List	0.1/-0.1
Ground Reaction Forces 1 Swing Phase	external_force_value	ground_force_1_vx<br>ground_force_1_vy<br>ground_force_1_vz	1/-1
Ground Reaction Forces 2 Swing Phase	external_force_value	ground_force_2_vx<br>ground_force_2_vy<br>ground_force_2_vz	1/-1
Ground Reaction Moments 1 Swing Phase	external_moment_value	ground_moment_1_mx<br>ground_moment_1_my<br>ground_moment_1_mz	1/-1
Ground Reaction Moments 2 Swing Phase	external_moment_value	ground_moment_2_mx<br>ground_moment_2_my<br>ground_moment_2_mz	1/-1

33. Save this settings file as VerificationOptimization\VOSettings.xml
34. Open VOSettings.xml in a text editor.
35. Inside your Ground Reaction Forces 1 Swing Phase and Ground Reaction Moments 1 Swing Phase constraint terms, copy and paste the following:
    `<time_ranges>0.17 0.48</time_ranges>`
36. Inside your Ground Reaction Forces 2 Swing Phase and Ground Reaction Moments 2 Swing Phase constraint terms, copy and paste the following:
    `<time_ranges>0.7 1</time_ranges>`
37. These constraint terms enforce that the ground reactions must be zero during swing phase. The time ranges are in units of normalized time (0 to 1), and make the constraint only active for a specified time window.
    a. We generally need these added constraints in DO but not in TO. This is because the ground reactions and joint positions/velocities are actively tracked in TO. They are not, however, tracked in DO, and so the DO solution can "cheat" by changing initial conditions or allowing ground reactions during swing phase.
38. Run the Matlab script VerificationOptimization\runVO.m
39. This VO run will take approximately 50 iterations to converge
40. If the optimization enters restoration mode as explained in the previous subproject, cancel the optimization and email the instructor.

As explained earlier, the purpose of VO is to be sanity check for your TO results and DO problem formulation. Your VO results should be closely identical to your TO results. If you get VO results that are different from your TO results, you have a problem. An example of a problematic VO run is inside VOResultsFailed. You can run the code on line 9 to explore the results. You will be asked about them in the deliverables section.

Module Task 2) Plan the Treatment

As we analyzed during NCP in the second module, the synergies in this run all have distinct functions throughout the gait cycle. These functions and the muscles in each synergy are summarized in the table below:

**Synergy Number**	**Synergy Function**	**Primary Muscle Groups in Synergy Vector**
1	Weight Acceptance	Hip Flexors, Hip Extensors
2	Propulsion	Ankle Plantarflexors
3	Propulsion/Weight Acceptance	Ankle Plantarflexors, Hip Abductors
4	Leg Swing	Hip Extensors, Hip Flexors
5	Flexion	Hip Flexors

We explain this subject's impairment by characterizing synergies as healthy, impaired, or compensatory based on their relative magnitudes between legs. The magnitudes of the synergies are listed in the table below:

Synergy #	Right Leg Activation Magnitude	Left Leg Activation Magnitude
1	0.30	0.21
2	0.18	0.22
3	0.18	0.3
4	0.12	0.17
5	0.22	0.22

The primary impairment for this subject occurs in right leg synergy 3, where the magnitude of synergy activation 3 is much higher on the non-paretic (left) side than on the paretic (right) side. In addition to the primary impairment, there are several compensatory synergies across both legs, where a synergy activation is marginally larger on one leg compared to the other. Synergy 1 is compensatory on the right leg. Synergies 2 and 4 are compensatory on the right leg. Synergy 5 is healthy.

These synergy asymmetries manifest themselves in the ground reaction forces. Specifically, the braking and propulsive impulses are highly asymmetric between the legs. The paretic side (right) has a lower propulsive and higher braking impulse compared to the non-paretic side. The specific numbers are shown in the table below.

	Propulsive Impulse	Braking Impulse
Right Leg	5.36	14.87
Left Leg	18.25	5.26

Your goal for this section is to plan a treatment to simulate for this subject. Your goal is to design a treatment that eliminates the asymmetries in synergy activations and analyze how the propulsive and braking impulses respond to that treatment. For simplicity, this treatment will only involve

scaling the synergy activation magnitudes, and as such, the shapes will not change. You may equalize the synergy activations using one of the following methods:

1. **Meet in the middle** – Calculate the average magnitude of both synergy activations, and scale the larger synergy activation down to the average, and the larger synergy activation up to the average.
2. **Increase the smaller synergy –** Scale the smaller synergy activation up to the magnitude of the larger synergy activation
3. **Decrease the larger synergy –** Scale the larger synergy activation down to the magnitude of the smaller synergy activation
4. **Any combination of the above.**

Your job for this task is to choose a method of equalizing synergy activations between the legs and calculate the scale factors required for your method. Fill out the following table with the scale factors you are applying to each synergy activation. The scale factors should be calculated with simple multiplications on the max magnitude of the synergy activations. If one synergy has a magnitude of 0.5 and you want it to increase to 0.75, then the scale factor is 0.75/0.5=1.5. <u>You will not be graded on whether you chose the "correct" method of equalizing the synergy activations. You will however be expected to justify *why* you chose your method of scaling synergy activations.</u>

The goal of our synergy FES protocol is to make the subject more symmetric by increasing or decreasing the magnitude of synergy activations. With FES treatments, it is important to consider the subject's limitations, and how FES can be used to change their synergies. Importantly, FES can only ever add to muscle activations. FES cannot reduce muscle activations. However, when equalizing synergy activations, we might want to reduce the magnitude of certain compensatory synergies. When planning a treatment of this type, we are making an implicit assumption that we are using FES to increase impaired synergies, and the subject will respond on their own by equalizing compensatory synergies without needing to directly stimulate those synergies. By extension, we are also assuming that the subject will be able to change their other synergies that are not being directly stimulated, which might not be true. This is a possible limitation that can only be truly studied by implementing our desired treatment on the original subject.

Synergy	Right Leg Scale Factor	Left Leg Scale Factor
1		
2		
3		
4		
5		

Module Task 3) Design Optimization

Now that you have a planned treatment design that equalizes the synergy activations, you can create a DO settings file that implements your treatment design. This DO will have two components – First we will scale the synergy activations as you designed in task 2, and then we will add cost terms that equalize the propulsive and braking impulses. will be done in two stages – First, we will just scale the synergy activations as prescribed in task 2. Next, we will add cost terms for the propulsive and braking impulses that track the scaled synergy activations.

1. Create a copy of VerificationOptimization\VOSettings.xml into DesignOptimization.
2. Rename this copied file to DOSettingsTemplate.xml.
3. Open DOSettingsTemplate.xml in a text editor and change the XML headings at the top and bottom of the file from <VerificationOptimizationTool> to <DesignOptimizationTool>
4. Load your post-JMP model UF_Subject_4_Scaled_JMP.osim into the OpenSim GUI
5. Open the Design Optimization GUI
6. Load your settings file DOSettingsTemplate.xml into the Design Optimization GUI.
7. Set the *Input Osimx File* to NeuralControlPersonalization\NCPResultsBilateral\UF_Subject_4_Scaled_JMP_gcp_mtp_mtp_ncp.osimx
    a. If the text box is red when you load the GUI in, you must re-select this file or the GUI will not be able to find it. The GUI does some light parsing through this file to locate ground reaction force and moment names.
8. Set the *Tracked Quantities and Initial Guess Directories* to VerificationOptimization\VOResults.
9. Set the *Results Directory* to DesignOptimization\DOResults
10. Set the *Surrogate Model Data Directory* to TrackingOptimization\surrogateData
11. Save this settings file as DOSettings.xml
12. Open DOSettings.xml in a text editor.
13. Inside <RCNLCostTermSet>, set **Synergy Controller Tracking** to **false**
14. Copy and paste the following cost terms into your <RCNLCostTermSet>

```
<RCNLCostTerm name="Synergy activation tracking right 1">
      <is_enabled>true</is_enabled>
      <type>controller_tracking</type>
      <controller_list>right_leg_1</controller_list>
      <scale_factor></scale_factor>
      <max_allowable_error>1</max_allowable_error>
</RCNLCostTerm>
<RCNLCostTerm name="Synergy activation tracking left 1">
      <is_enabled>true</is_enabled>
      <type>controller_tracking</type>
      <controller_list>left_leg_1</controller_list>
      <scale_factor></scale_factor>
      <max_allowable_error>1</max_allowable_error>
</RCNLCostTerm>
<RCNLCostTerm name="Synergy activation tracking right 2">
      <is_enabled>true</is_enabled>
      <type>controller_tracking</type>
      <controller_list>right_leg_2</controller_list>
      <scale_factor></scale_factor>
      <max_allowable_error>1</max_allowable_error>
</RCNLCostTerm>
<RCNLCostTerm name="Synergy activation tracking left 2">
      <is_enabled>true</is_enabled>
      <type>controller_tracking</type>
```

```
        <controller_list>left_leg_2</controller_list>
        <scale_factor></scale_factor>
        <max_allowable_error>1</max_allowable_error>
</RCNLCostTerm>
<RCNLCostTerm name="Synergy activation tracking right 3">
        <is_enabled>true</is_enabled>
        <type>controller_tracking</type>
        <controller_list>right_leg_3</controller_list>
        <scale_factor></scale_factor>
        <max_allowable_error>1</max_allowable_error>
</RCNLCostTerm>
<RCNLCostTerm name="Synergy activation tracking left 3">
        <is_enabled>true</is_enabled>
        <type>controller_tracking</type>
        <controller_list>left_leg_3</controller_list>
        <scale_factor></scale_factor>
        <max_allowable_error>1</max_allowable_error>
</RCNLCostTerm>
<RCNLCostTerm name="Synergy activation tracking right 4">
        <is_enabled>true</is_enabled>
        <type>controller_tracking</type>
        <controller_list>right_leg_4</controller_list>
        <scale_factor></scale_factor>
        <max_allowable_error>1</max_allowable_error>
</RCNLCostTerm>
<RCNLCostTerm name="Synergy activation tracking left 4">
        <is_enabled>true</is_enabled>
        <type>controller_tracking</type>
        <controller_list>left_leg_4</controller_list>
        <scale_factor></scale_factor>
        <max_allowable_error>1</max_allowable_error>
</RCNLCostTerm>
<RCNLCostTerm name="Synergy activation tracking right 5">
        <is_enabled>true</is_enabled>
        <type>controller_tracking</type>
        <controller_list>right_leg_5</controller_list>
        <scale_factor></scale_factor>
        <max_allowable_error>1</max_allowable_error>
</RCNLCostTerm>
<RCNLCostTerm name="Synergy activation tracking left 5">
        <is_enabled>true</is_enabled>
        <type>controller_tracking</type>
        <controller_list>left_leg_5</controller_list>
        <scale_factor></scale_factor>
        <max_allowable_error>1</max_allowable_error>
```

```
</RCNLCostTerm>
```

15. Inside each cost term, fill out `<scale_factor>` with the scale factors you calculated in task 2.
16. Inside of your `<RCNLCostTermSet>`, copy and paste the following cost terms.

```
<RCNLCostTerm name="Propulsive impulse">
      <is_enabled>true</is_enabled>
      <type>propulsive_impulse_goal</type>
      <hindfoot_body_list>calcn_r calcn_l</hindfoot_body_list>
      <error_center>10.7</error_center>
      <max_allowable_error>4.3</max_allowable_error>
</RCNLCostTerm>
<RCNLCostTerm name="Braking impulse">
      <is_enabled>true</is_enabled>
      <type>braking_impulse_goal</type>
      <hindfoot_body_list>calcn_r calcn_l</hindfoot_body_list>
      <error_center>10.7</error_center>
      <max_allowable_error>4.3</max_allowable_error>
</RCNLCostTerm>
```

   a. This cost term sets a design goal for the optimization to equalize the propulsive and braking impulses between the feet. The cost term imposes that the braking and propulsive impulses for both the left and the right feet must be 10.1 Ns. The value of 10.1 was obtained by averaging the propulsive impulses between the legs before the treatment, and then assuming the braking impulses should also be equal to that number.

17. Disable all controller tracking cost terms added in step 14.
18. Re-enable your original controller tracking cost term that tracks all synergy activations with no scale factors. This should be the same controller tracking term that was used in VO.
19. Run `DOSettings.xml`
20. After running DO, open the file `calcPropAndBrakeImpulses.xml` and change the initial guess directory to be the results directory from your stage 1 DO run. Run line 11 in `RunDO.m` and record the calculated propulsive and braking impulses.
21. Edit `calcPropAndBrakeImpulses.xml` and change the initial guess directory to be the results directory from your stage 2 DO run. Run line 11 in `RunDO.m` and record the calculated propulsive and braking impulses.

Deliverables:

Inside one PDF file, submit all plots outputted by your VO and DO runs, and answer the following questions.

1. One purpose of VO is to diagnose potential problems with a TO solution before getting caught up in a DO run. Diagnosing problems with a TO solution is rarely straightforward, however. Inside the VO results directory named `VOResultsFailed`, you can see VO results that tracked the synergy activations nearly perfectly, but did not reproduce the same joint angles, ID loads, and ground reactions. The diagnosis for this problem was that the kinetic consistency term in TO was too loose and needed to be tightened. Changing kinetic consistency in TO from 0.1 to 0.01 fixed the problem. **Explain why too loose of a kinetic consistency in TO might lead to the result seen in `VOResultsFailed`.** Remember that a kinetic consistency constraint enforces that the joint moments produced by muscles must be equal to the ID loads to within a given tolerance.
2. Fill out the table with your synergy activation scale factors:

Synergy	Right Leg Scale Factor	Left Leg Scale Factor
1		
2		
3		
4		
5		

3. Explain and justify the method you used to decide on synergy activation scale factors.
4. Fill out the table with the propulsive and braking impulses from your TO and DO runs.

		Propulsive Impulse	Braking Impulse
TO Results	Right Leg		
	Left Leg		
DO Results	Right Leg		
	Left Leg		

5. Do you think your treatment design was a success? Why or why not? If not, what do you think you could do to make it better?
6. If you were to do another DO iteration to get a better treatment design, what would you change in your problem formulation?
7. Given your treatment design, which synergies would you stimulate with FES, and which synergies would you assume that the subject can change on their own in response to the FES treatment?
8. Do you think that your treatment design can be readily translated into a clinical synergy FES stimulation pattern? Why or why not? If not, what could you change to more accurately simulate FES for this subject?
9. What are the primary limitations of this computational treatment design?

References

Ao, Di, and Benjamin J. Fregly. 2024. “Comparison of Synergy Extrapolation and Static Optimization for Estimating Multiple Unmeasured Muscle Activations during Walking.” *Journal of NeuroEngineering and Rehabilitation* 21 (1): 194. https://doi.org/10.1186/s12984-024-01490-y.

Ao, Di, Marleny M. Vega, Mohammad S. Shourijeh, Carolynn Patten, and Benjamin J. Fregly. 2022. “EMG-Driven Musculoskeletal Model Calibration with Estimation of Unmeasured Muscle Excitations via Synergy Extrapolation.” *Frontiers in Bioengineering and Biotechnology* 10 (September): 962959. https://doi.org/10.3389/fbioe.2022.962959.

Cheung, Vincent C. K., Chuanxin M. Niu, Si Li, Qing Xie, and Ning Lan. 2019. “A Novel FES Strategy for Poststroke Rehabilitation Based on the Natural Organization of Neuromuscular Control.” *IEEE Reviews in Biomedical Engineering* 12: 154–67. https://doi.org/10.1109/RBME.2018.2874132.

Fregly, Benjamin J. 2021. “A Conceptual Blueprint for Making Neuromusculoskeletal Models Clinically Useful.” *Applied Sciences* 11 (5): 5. https://doi.org/10.3390/app11052037.

Hammond, Claire V., Spencer T. Williams, Marleny M. Vega, et al. 2025. “The Neuromusculoskeletal Modeling Pipeline: MATLAB-Based Model Personalization and Treatment Optimization Functionality for OpenSim.” *Journal of NeuroEngineering and Rehabilitation* 22 (1): 112. https://doi.org/10.1186/s12984-025-01629-5.

Levine, Jackson T., Xin S. Yu, Rebecca Muñoz, et al. 2025. “Synergy-Based Functional Electrical Stimulation for Gait Rehabilitation in Chronic Stroke: A Pilot Study.” Preprint, medRxiv, September 10. https://doi.org/10.1101/2025.05.21.25328035.

Meyer, Andrew J., Carolynn Patten, and Benjamin J. Fregly. 2017. “Lower Extremity EMG-Driven Modeling of Walking with Automated Adjustment of Musculoskeletal Geometry.” *PLOS ONE* 12 (7): e0179698. https://doi.org/10.1371/journal.pone.0179698.

Silder, Amy, Ben Whittington, Bryan Heiderscheit, and Darryl G. Thelen. 2007. “Identification of Passive Elastic Joint Moment–Angle Relationships in the Lower Extremity.” *Journal of Biomechanics* 40 (12): 2628–35. https://doi.org/10.1016/j.jbiomech.2006.12.017.

Ting, Lena H., Hillel J. Chiel, Randy D. Trumbower, et al. 2015. “Neuromechanical Principles Underlying Movement Modularity and Their Implications for Rehabilitation.” *Neuron* 86 (1): 38–54. https://doi.org/10.1016/j.neuron.2015.02.042.

Zajac, F. E. 1989. “Muscle and Tendon: Properties, Models, Scaling, and Application to Biomechanics and Motor Control.” *Critical Reviews in Biomedical Engineering* 17 (4): 359–411.
